# A spatial and temporal transcriptomic atlas of mouse intestinal regeneration in the jejunum

**DOI:** 10.1038/s41467-026-77292-8

**Published:** 2026-09-03

**Authors:** Rachel Ofer, Tyler Tran, Ping He, Xia Qiu, Xinyu Hu, Emmanuel Zachariah, Curtis Krier, Ankit Saxena, Lei Chen, Jiekun Yang, Michael P. Verzi

**Affiliations:** 1 Department of Genetics, Rutgers University, Piscataway, NJ USA; 2 Human Genetics Institute of New Jersey, Rutgers University, New Brunswick, Piscataway, NJ USA; 3 Rutgers Cancer Institute of New Jersey, Rutgers University, New Brunswick, Piscataway, NJ USA; 4 Department of Medicine, Rutgers University Robert Wood Johnson Medical School, New Brunswick, Piscataway, NJ USA; 5 Center for Immune Monitoring and Flow Cytometry, Rutgers Cancer Institute of New Jersey, New Brunswick, Piscataway, NJ USA; 6 School of Life Science and Technology, Key Laboratory of Developmental Genes and Human Disease, Southeast University, Nanjing, China; 7 Institute of Microphysiological Systems, Southeast University, Nanjing, China; 8 Rutgers Center for Lipid Research, New Jersey Institute for Food, Nutrition & Health, Rutgers University, New Brunswick, Piscataway, NJ USA; 9 NIEHS Center for Environmental Exposures and Disease (CEED), Rutgers EOHSI, Piscataway, NJ USA

**Keywords:** Intestinal stem cells, Inflammatory bowel disease, Gene expression, Transcriptomics, Immunology

## Abstract

The intestinal epithelium exhibits a remarkable capacity for regeneration following injury, yet the spatial and temporal dynamics of the damage-repair cycle remain incompletely understood. We present a spatial transcriptomics atlas of ionizing radiation-induced injury and repair in the mouse jejunum, capturing mRNA from 16,921 genes across approximately 26 million bins at 2-µm resolution in 23 biological samples spanning six days post-irradiation. Analysis reveals transcriptional patterns and niche signals undetectable by bulk or single-cell approaches, including spatially restricted interferon-target gene activation, an altered differentiation trajectory in the lower villus during early repair, two transcriptionally distinct crypt regenerative cell populations, and global transcriptional upregulation consistent with hypertranscription as a hallmark of intestinal regeneration, with evidence for hypertranscription extending to human Crohn’s disease. In this work, we provide a spatiotemporal atlas of mouse intestinal regeneration at subcellular 2-µm resolution that will inform future therapeutic strategies for inflammatory bowel diseases and gastrointestinal pathologies arising from injury or cancer therapy.

## Abstract (web summary)

The intestinal epithelium efficiently regenerates after injury, yet the dynamics underlying this process are not fully understood. Here, the authors use spatial transcriptomics to map the mouse intestine over time after radiation injury, revealing shifts in villus zonation, spatially restricted interferon signaling, altered stem cell populations and a surge in gene transcription that may help fuel recovery.

## Introduction

The intestine provides one of nature’s best examples of how the evolution of structure is associated with function. The elegant spatial composition of the repeating pattern of crypts and villi provides immense surface area to facilitate absorption of nutrients and water, while the vast surface area also, in turn, presents a large interface with microbial communities and is accompanied by an active mucosal immune system. The highly proliferative nature of the tissue also makes it susceptible to damage. During acute intestinal injury from pathogens, chemotherapies, radiation therapies, or inflammatory diseases, intestinal homeostasis is disrupted, triggering a cascade of regenerative responses. Damaged epithelial cells initially undergo repair or cell death, followed by immune cells and stromal fibroblasts providing regenerative signals, and finally, regenerative stem cells undergo hyperproliferation to recover lost epithelium. Ultimately, the damaged tissue is remodeled to restore the spatial organization of the homeostatic intestine^1–4^.

Technologies enabling in situ profiling of gene expression^5,6^ are beginning to shed light on the damage-repair cycle in the intestine. In models of mouse colitis, spatially resolved transcriptomic studies have profiled subsets of genes to make discoveries such as alterations in stromal fibroblasts^7^ or spatially restricted signaling shifts in the regenerating epithelium^8^. Gene panels have been probed in situ to uncover the role of CD8 T cells during viral infection of the small intestine^9^. Radiation therapy is a pillar of cancer treatment but causes enteropathy and reduced quality of life^10^. In mice, ionizing radiation (IR) causes acute epithelial damage and crypt loss, triggering surviving epithelial cells to enter a regenerative state^11^. Regenerating cells shift their transcriptional programs to meet the demands of rapid expansion to restore homeostasis^1,12,13^. IR-induced injury in mice is a valuable model for studying epithelial regeneration^14^. However, substantial knowledge gaps remain regarding the spatial and temporal dynamics in the transcriptomes of cells that initiate and drive this regenerative response.

Recent technological advances allow nearly the whole transcriptome to be detected at a resolution of 2-µm bins^15,16^. Here, we analyze four high-resolution spatial transcriptomics datasets across 23 biological samples spanning six days post-IR using Visium HD Spatial Gene Expression (‘Visium HD’). This approach enables simultaneous profiling of almost the full genome at near-single-cell resolution. By capturing spatial and temporal gene expression dynamics, the data reveal changes in cellular behavior, tissue architecture, and cell–cell interactions during intestinal regeneration, including shifts in the mechanics of epithelial differentiation, activation of hypertranscription by regenerating epithelium, and an altered stromal signaling environment and regenerative stem cell populations during repair. The reproducibility across independent experiments and easy data accessibility provide a robust and valuable resource (GSE303705; Supplementary Note 1) for studying epithelial repair and tissue remodeling.

## Results

### Spatial transcriptomic resource for intestinal regeneration

**Fig. 1:**
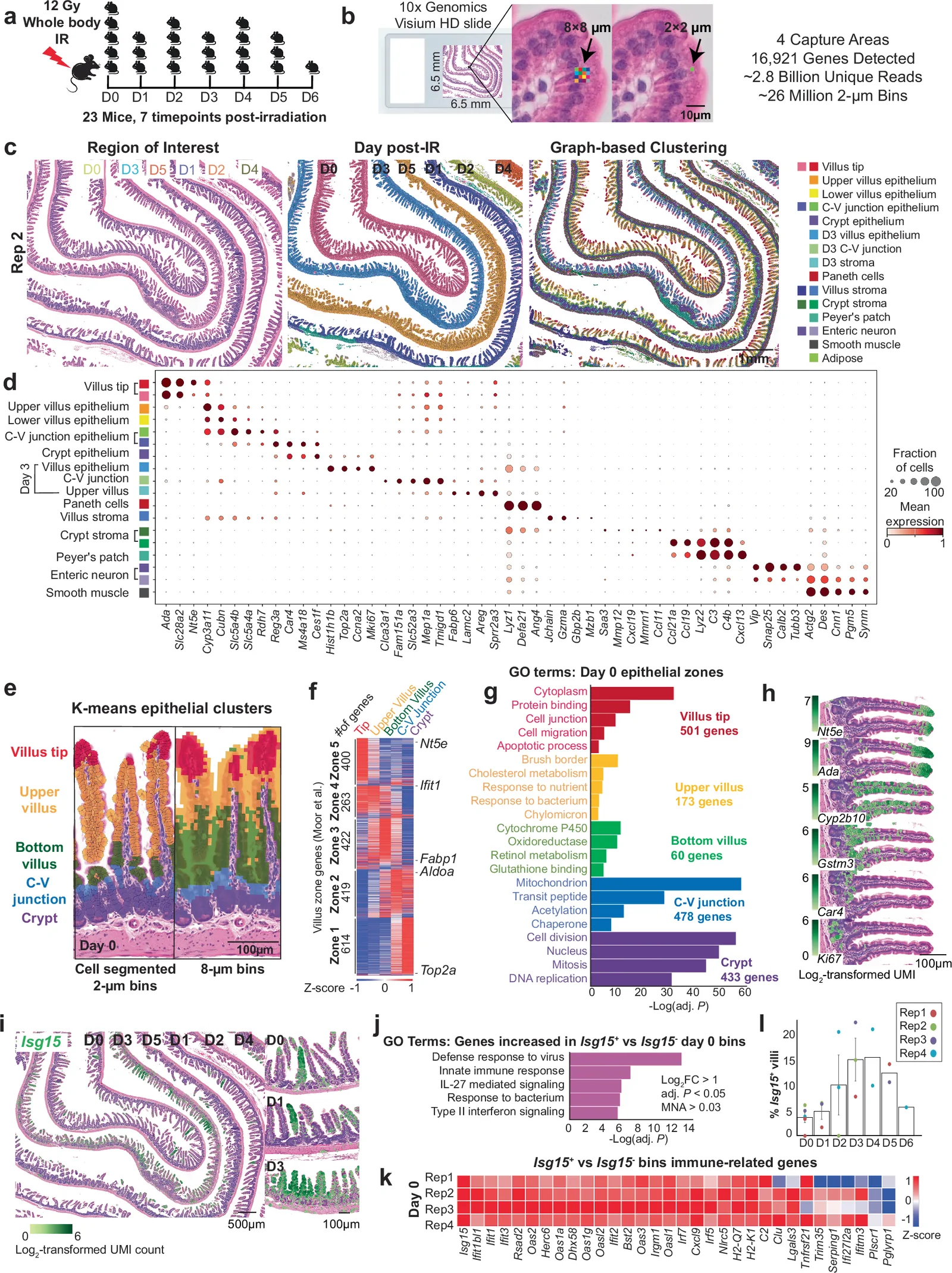
A spatial transcriptomic atlas of post-irradiation intestinal regeneration. **a** Visium HD experimental design: *n* = 4 capture areas; 23 biological samples; D0 (non-irradiated), *n *= 5; D1, *n* = 3; D2, *n* = 4; D3, *n* = 3; D4, *n* = 4; D5, *n* = 3; D6, *n* = 1). Created in BioRender. Ofer, R. (2026) https://BioRender.com/k6p4uhz. **b** ~ 2.8 billion unique reads; ~ 26 million 2-µm bins across 4 capture areas, 16,921 genes detected. **c** Representative capture area (rep2) by timepoint and graph-based clustering of cell-segmented 2-µm bins; similar clustering observed across all replicates (Supplementary Fig. 1a). **d** Mean normalized log_2_ expression of top upregulated genes per cluster. **e** Five crypt-villus epithelial clusters, non-irradiated tissue, rep2; cell-segmented 2-µm or 8-µm bins. Non-epithelial bins excluded. **f** Z-score heatmap of the villus zonation gene set^17^ (Moor et al.) across the five D0 epithelial clusters. Z-scores were calculated from log_2_FC values of differential expression between the five epithelial clusters (Loupe Browser; two-sided negative binomial-based test with Benjamini–Hochberg correction). **g** GO enrichment of the five D0 epithelial clusters; gene number per zone listed. DEGs (as in (**f**)) were filtered (log_2_FC > 1, adj. *P* < 0.05, MNA > 0.03) and analyzed by GO enrichment in DAVID^73,74^ (modified Fisher’s exact test (EASE score), Benjamini–Hochberg correction). Full GO results in Supplementary Data 1. **h** Loupe Browser spatial expression of villus zonation markers; similar pattern observed across replicates. **i** *Isg15*^*+*^ expressed as villi clusters at days 0, 1 and 3 post-IR (log_2_-transformed UMI); a similar pattern observed across replicates. **j** GO enrichment, *Isg15*^+^ vs. *Isg15*^*−*^ bins, D0; upregulation of interferon-associated pathways. Smooth muscle, enteric neuron, crypt and crypt-stroma bins excluded. Top enriched terms selected. Gene sets from GO^21^: GO:0051607, GO:0009615, GO:0002376, GO:0045087, GO:0045071. Complete GO results in Supplementary Data 2. **k** Z-score heatmap of interferon-stimulated genes in *Isg15*^+^ bins at D0 across all replicates. Z-scores calculated as in (**f**) for *Isg15*^+^ vs. *Isg15*^*−*^ bins. **l** Percentage of *Isg15*^*+*^ villus bins across the four biological replicates. *n* = 5(D0), 3(D1), 3(D2), 3(D3), 2(D4), 2(D5), 1(D6); 2 of the D4 samples included. Mean ± SEM. Scale bar: 10 µm (**b**); 1 mm (**c**); 100 µm (**e**,** h**,** i**) and 500 µm (**i**). Source data are provided as a Source Data file.

Intestinal damage from ionizing radiation (IR) is a limiting factor for radiation therapy and a widely used model to understand the intestinal damage-repair cycle^10,11^. To better appreciate the spatial and temporal response of the intestine to IR, we conducted a spatial transcriptomics time-series of the mouse small intestine after exposure to 12 Gy (gray) IR. Jejunal segments from 23 independent biological samples spanning days 0 (D0) through 6 post-IR (Fig. 1a) were embedded into four formalin-fixed paraffin-embedded blocks and processed using the Visium HD platform (Supplementary Fig. 1a). Visium HD uses a continuous array of 2-µm barcoded probe-capture grids to determine RNA expression in the tissue with high spatial resolution and near-genome scale breadth^15^. A representative bin is shown to illustrate near-cellular resolution (Fig. 1b). Across the four capture areas, we detected 16,921 genes (minimum expression threshold: detection in at least 50 cells), spanning ~ 26 million 2-µm bins at a sequencing depth of ~ 2.8 billion unique reads (Fig. 1b and Supplementary Fig. 1b–d). Unsupervised clustering of the bins revealed striking patterns of gene expression, consistent with known spatial patterns in intestinal biology (Fig. 1c, d and Supplementary Fig. 1e). For example, zonation of the villus epithelium was apparent, with villus tip, middle, and bottom regions clustering separately (Fig. 1e). Distinct bins expressing markers of smooth muscle or enteric glial cells (*Gfap*) comprised clusters localized at the periphery (Fig. 1d and Supplementary Fig. 1f). Stromal cell populations were detected in the villus core and inter-crypt regions and expressed genes associated with immune cell populations and fibroblasts (Fig. 1d and Supplementary Fig. 1e, g, h). The data were probed to detect canonical marker genes; for example, markers of fibroblasts (*Pdgfra*), B cells (*Jchain*), T cells (*Cd8a*), M cells (*Gp2*), tuft cells (*Avil*), proliferative cells (*Mki67*), and regenerative stem cells (*Clu)* were visualized in space and time (Supplementary Fig. 1g–j).

Unsupervised *k*-means clustering of bins’ transcriptomes spanning the crypt-villus axis identified five distinct epithelial zones: villus tip, upper villus, bottom villus, crypt-villus junction, and crypt epithelium (Fig. 1e). The distinct domain of the crypt-villus junction was reliably preserved in the current study, unlike in other studies using conventional cell isolation methods where most epithelial preparations are damaged at the crypt-villus junction, potentially compromising the results. Differential gene expression (DEG) analysis between the epithelial zones yielded gene sets characteristic of each region (log_2_ fold change (log_2_FC) > 1, adj. *P* < 0.05, MNA > 0.03; Supplementary Data 1), and the transcriptomes of each zone correlated strongly with previously described villus zonation signatures^17^ (Fig. 1f). Zonation patterns were consistent across independent experiments (Supplementary Fig. 1k). GO term analysis corroborated established functions of epithelial zonation^17^ (Fig. 1g and Supplementary Data 1). Examples of spatially localized expression of zone-specific genes along the crypt-villus axis are shown in Fig. 1h.

Finally, unique features of intestinal biology that might be missed by single-cell approaches could be captured in the spatial datasets. For example, a baseline level of interferon signaling appeared in control animals in an intriguing spatial pattern: roughly 5% of villi exhibited elevated expression of interferon pathway markers, such as *Isg15*, sporadically along the tissue of non-IR samples (Fig. 1i). The patchy homeostatic *Isg15*^*+*^ villus expression pattern is morphologically and transcriptionally consistent with previously observed sporadic localization of epithelial interferon stimulation by microbiota under homeostatic conditions^18–20^. To better appreciate this pattern, we compared gene expression between *Isg15*^+^ and *Isg15*^*-*^ bins (Fig. 1j and Supplementary Data 2). This analysis revealed a broad panel of interferon-stimulated genes^21^ elevated in *Isg15*-hi villi (Fig. 1k), including *Ifit1* (Supplementary Fig. 2a), previously shown to mark epithelial pockets with high interferon-stimulated gene (ISG) expression^19^. We identified differentially expressed genes between *Ifit1*^*+*^ and *Ifit1*^*−*^ day 0 homeostatic villus epithelial bins (188 genes; log_2_FC > 2, adj. *P* < 0.05, MNA > 0.05; Supplementary Data 3). GO analysis of *Ifit1*^*+*^ enriched genes revealed significant enrichment of antiviral and interferon-related pathways, consistent with the published *Ifit1*^*+*^ transcriptional signature defined by laser capture microdissection^19^ (Supplementary Data 3). Gene set enrichment analysis^22^ (GSEA) confirmed strong concordance between our spatial *Ifit1*^*+*^ bins and the laser capture-defined gene set^19^ (Supplementary Fig. 2a), demonstrating that the focal ISG expression in our data reflects a highly similar interferon-driven epithelial program to that previously described^19^ but without the need for microdissection. Co-expression analysis confirmed that most bins expressing one IFN-pathway target also co-expressed others (Supplementary Fig. 2b).

During the damage-repair cycle, increasing numbers of villi exhibited ISG expression (Fig. 1l), and these villi tended to cluster in groups (Ripley’s L function, significant at day 3; Supplementary Fig. 2c), suggesting potential intercellular communication facilitating signal spread between adjacent villi. We hypothesized that signal spread between adjacent villi could occur through the inter-villus junction epithelium and/or the crypt epithelium, so we examined the spatial distribution of *Isg15* expression for mechanistic insight. *Isg15*^*+*^ bins within villus epithelial clusters increased markedly at day 3 (D3, 70%) relative to day 0 (D0, 18%; Supplementary Fig. 2d). Strikingly, the proportional increase was even greater at the inter-villus junction domain (D3, 36% vs. D0, 5%), raising the possibility that IFN signals spread between adjacent villi via the inter-villus junctional epithelium (Supplementary Fig. 2 d). We next looked for genes unique to IFN-spreading versus non-spreading regions and identified *Ifit2* as a spatially restricted marker, present almost exclusively at inter-villus junctions of clustered *Isg15*^*+*^ villi at day 3, while absent at day 0 (Supplementary Fig. 2e, f). This expression pattern suggests that *Ifit2* marks a damage-induced, junction-specific activation state associated with signal spreading rather than a general ISG response. Differential expression analysis comparing *Ifit2*^*+*^ to *Ifit2*^*−*^ junction bins at day 3 identified 23 enriched genes (log_2_FC > 1, adj. *P* < 0.05) as candidates for functional involvement in local IFN signal spread (examples of genes in Supplementary Fig. 2g; full gene list in Supplementary Data 3). Together, these spatially informed candidates point to a functionally distinct, junction-restricted IFN-active population during regeneration, warranting future investigation.

We use these examples to present a resource for the field to explore, generate, and test hypotheses concerning the mechanisms controlling intestinal damage and repair, made as an easily accessible dataset (GSE303705; Supplementary Note 1).

### Shifts in villus epithelial patterning post-IR

**Fig. 2:**
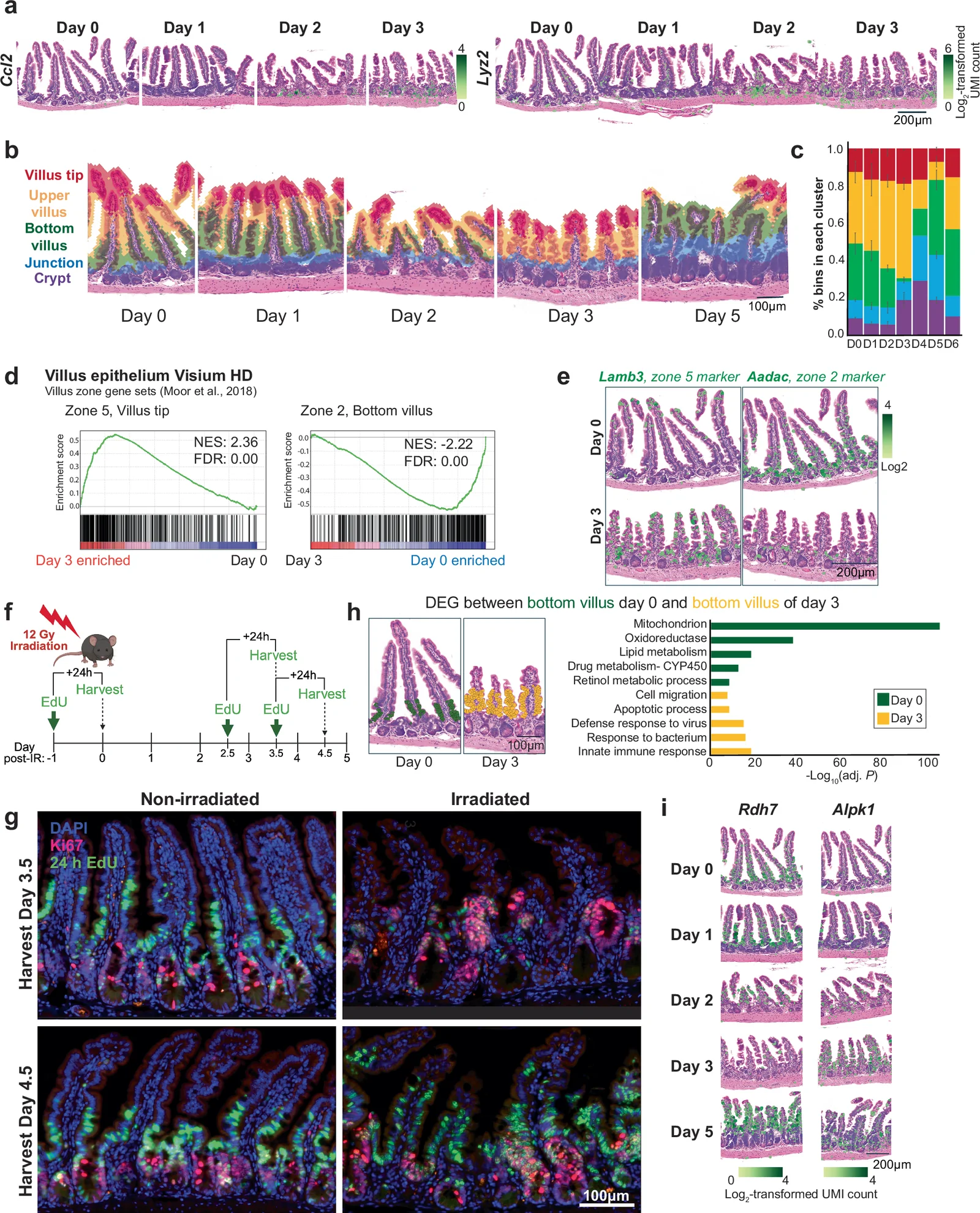
The villus bottom epithelium exhibits a transcriptional shift towards maturity and innate immunity at day 3 post-IR. **a** Log_2_ expression of monocyte markers *Ccl2* and *Lyz2*; increased expression from D0 to D3 post-IR, rep2. **b** Five epithelial clusters (villus-tip, upper villus, bottom villus, crypt-villus junction and crypt) across all timepoints, rep2; *k*-means clustering restricted to epithelial bins. The post-mitotic villus-bottom cells (green, D0) are absent at D3 and replaced by a cluster associated with the upper villus at D0 (yellow). Similar clustering observed in rep3 and rep4 (Supplementary Fig. 3a). **c** Percent distribution of epithelial clusters, all timepoints and replicates; proportion of epithelial bins per cluster normalized to total epithelial bins per sample. *n* = 5 (D0), 3(D1), 3(D2), 3(D3), 2(D4), 3(D5), 1(D6); 2 of D4 samples included. Error bars: SEM across all replicates. **d** GSEA of differential expression between D3 and D0 bottom-villus clusters (MNA > 0.05) against Moor et al.^17^ zonation gene sets. The villus-tip gene set (zone 5) is enriched in D3 villus epithelium, and the bottom-villus set (zone 2) in D0. **e** Spatiotemporal expression (log_2_-transformed UMI) of zonation markers *Lamb3* (zone 5) and *Aadac* (zone 2) in D0 and D3 post-IR. **f** Schematic of EdU-based proliferation assay. Mice received EdU at days 2.5 and 3.5 post-IR; tissues collected 24-h later. *n* = 2 per timepoint (control, D3.5, D4.5). Created in BioRender. Ofer, R. (2026) https://BioRender.com/5gyb85y. **g** 24-h EdU pulse-chase reveals retention of proliferative epithelial cells in crypts at day 3.5 post-IR followed by upward migration by day 4.5, suggesting delayed new-cell contribution to the villus base during regeneration. EdU and DAPI counterstain; *n* = 2 mice per condition. **h** DEG, bottom villus cluster, D0 vs. D3; representative GO terms shown. DEG criteria: log_2_FC > 1, adj. *P* < 0.05, MNA > 0.03; GO calculated in DAVID^73,74^, (modified Fisher’s exact test (EASE score), Benjamini–Hochberg correction. Full gene list and GO results in Supplementary Data 5. **i** Spatiotemporal expression (log_2_-transformed UMI) of *Rdh7, Alpk1* across post-IR timepoints. Scale bar: 200 µm (**a**,** e**,** i**); 100 µm (**b**,** g**,** h**). Source data are provided as a Source Data file.

Dynamic expression of cell lineage markers could be observed throughout the regenerative timeline. As one example of the use of the data for hypothesis testing, macrophage recruitment to damaged crypts at days 2–3 post-IR was visualized through the dynamic expression of monocyte markers, *Ccl2* and *Lyz2* (Fig. 2a), consistent with the role of mucosal macrophages in intestinal regeneration^23–25^. As an example of hypothesis generation from the spatial dataset, unbiased *k*-means clustering revealed that while regions such as smooth muscle and the enteric neurons remained transcriptionally stable throughout the damage-repair cycle, clusters associated with the bottom villus epithelium underwent sufficient transcriptional reprogramming to adopt new cluster identities at day 3 post-IR (Fig. 2b). Specifically, while villus bottom bins from homeostatic and other post-IR timepoints clustered together (green, Fig. 2b), day 3 post-IR bins shifted to a cluster typically associated with the upper villus epithelium (yellow, Fig. 2b), an observation that was consistent across replicates (quantified across replicates in Fig. 2c and Supplementary Fig. 3a). Thus, despite their spatial position at the villus base, day 3 epithelial cells exhibit a transcriptomic signature characteristic of more mature upper-villus epithelium.

To further characterize the transcriptional maturation of the day 3 villus bottom epithelium, we applied villus zonation gene signatures^17^ (Supplementary Data 4) to differential expression analysis between day 0 and day 3 villus bottom bins using GSEA (Fig. 2d). Day 0 lower villus bins were enriched for zone 2 (lower villus) gene signature, as expected. However, relative to lower villus bins at day 0, day 3 lower villus bins were enriched for zone 5 (villus tip) gene signature, characteristic of the most mature villus epithelium. Analysis of villus zonation gene set scores along the crypt-to-tip axis further supports this maturation shift (Supplementary Fig. 3b); in homeostasis (day 0), the lower villus zone 2 gene set (yellow line) peaks in the junction region ( ~ 100 µm distance from the smooth muscle layer), whereas at day 3 post-IR its expression is reduced by approximately half. Conversely, the upper villus gene sets (zone 4, orange; zone 5, pink) show increased expression at day 3 beginning at the crypt-villus junction. Representative villus zonation genes are shown: *Lamb3* (zone 5, villus tip) is enriched at the day 3 villus base, while *Aadac* (zone 2, bottom villus) is enriched at day 0 relative to day 3 (Fig. 2e). These results collectively suggest the villus base epithelium acquires a more mature transcriptional identity at day 3 post-IR.

A potential explanation for the more mature epithelial state observed in villus base cells at day 3 post-IR is a change in the contribution of newly differentiated cells from the crypt to the villus. To explore whether migration of cells from the crypt onto the villus is altered post-IR, we conducted EdU pulse-chase experiments (Fig. 2f). Based upon previous work^23^ proliferation resumes around 2.5 days post-IR; therefore, we conducted a 24-h pulse-chase experiment at 2.5 and 3.5 days post-IR. EdU-labeled cells from mice injected at day 2.5 post-IR (harvested at day 3.5 post-IR) remained in the crypts rather than migrating onto the villi as occurs during homeostasis (Fig. 2g; proliferation marked by KI67).

While EdU labeling alone is insufficient to demonstrate epithelial maturation, the pattern observed at day 3.5 post-IR is corroborated by previously published 24-h BrdU pulse-chase data from our lab^23^, which showed a marked reduction in villus BrdU incorporation between days 1 and 3 post-IR, followed by resumed contribution of new cells to the villus base by days 4–5, consistent with the rise of EdU-labeled cells onto the villi observed at day 4.5 (Fig. 2g). We therefore observe that a transient pause in crypt-to-villus contribution at the day 3 timepoint correlates with the ability for existing villus bottom cells to acquire a more mature transcriptional state. Direct lineage tracing or live imaging of cell migration may be needed to further validate these conclusions.

A feature of the upper villus epithelium at day 0 is upregulation of genes associated with response to bacteria (Fig. 1g). GO term analysis comparing day 0 and day 3 bottom villus epithelium revealed that day 3 bottom villus epithelial cells adopt a more innate-immune-active transcriptional state (Fig. 2h and Supplementary Data 5), exemplified by elevated expression of innate immune effectors such as *Alpk1* (Fig. 2i). Conversely, *Rdh7*, associated with homeostatic villus function, was downregulated at day 3 (Fig. 2i). These transcriptional shifts were consistent across replicates (Supplementary Fig. 3c and Supplementary Data 6). Collectively, the day 3 villus bottom epithelium acquires transcriptional features of more mature upper-villus cells while simultaneously upregulating innate immune genes, possibly reflecting the inflammatory milieu accompanying active tissue injury.

### Regenerating crypt cells split into two stem-like states

**Fig. 3:**
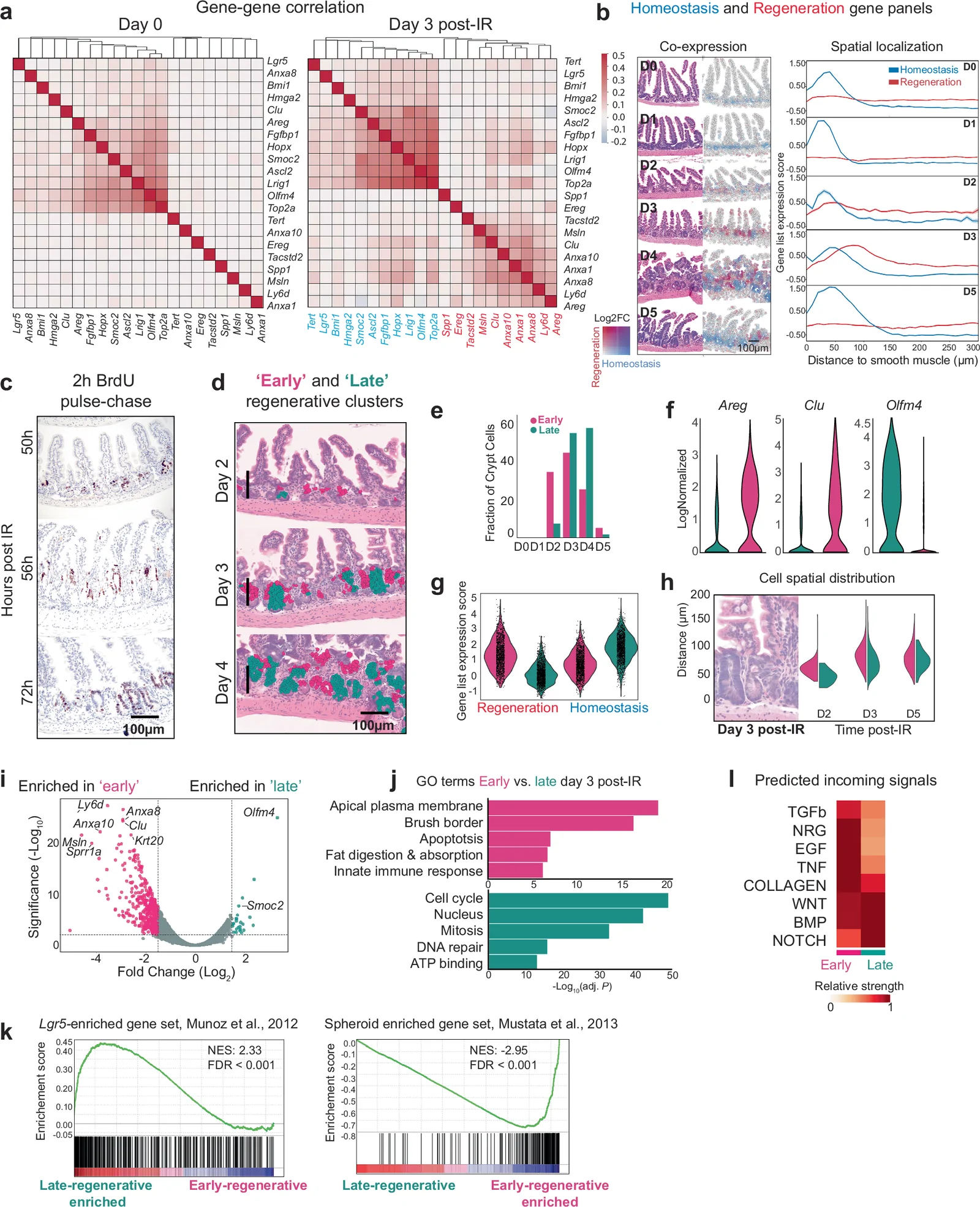
Subclusters of crypt epithelium partition into cells with early- and late-regenerative stem cell features. **a** Gene-gene co-expression of homeostatic genes (blue) and regenerative stem cell genes (red), D0 to D3 post-IR, rep2. **b** Spatial gene-set scores show non-overlapping expression; regenerative signatures elevated in upper crypt between days 2–5; quantified in 10-µm windows from smooth muscle cluster. **c** BrdU labeling post-IR shows early proliferative activity at crypt tops, coinciding with peak regenerative marker expression (representative of 3 biological replicates). **d** Crypt epithelium sub-clustering identifies two transcriptionally distinct populations, absent at D0, emerging on days 2–4 post-IR, rep2. **e** Quantification across timepoints and spatial position; one subcluster appears earlier/higher in the crypt (pink), the other later/lower (green); denoted early and late clusters, though overlapping on days 2–5 post-IR. **f** Violin plots of log-normalized expression (UMI counts normalized to the total UMI count per bin) of regenerative (*Clu, Areg*^26,27,29^) and homeostatic (*Olfm4)* markers in early (*n* = 1497 bins) and late (*n* = 1848 bins) crypt subclusters, D3 post-IR, rep2. **g** Homeostatic and regenerative gene-set scores reciprocally elevated in the early and late clusters. **h** Crypt-position quantification confirms the early cluster lies upper-crypt relative to the late cluster. Bins per timepoint (early, late): D2(108, 23); D3(1497, 1848); D4(54, 230); D5(278, 86), rep2. **i** Volcano plot of DEGs between early- and late-regenerative clusters (D3 post-IR); Loupe Browser, two-sided negative binomial-based test, Benjamini–Hochberg correction; plotted with VolcaNoseR). Early cells are enriched for fetal-like genes (*Clu, Anxa1*), and late cells for stem genes (*Smoc2*, *Olfm4*). Early, 666 genes; late, 252 genes (adj. *P* < 0.05, MNA > 0.03, log_2_FC > 1). **j** GO analysis of DEGs in (**i**); statistics as in Fig. 1g. **k** GSEA, D3 post-IR, early vs. late regenerative clusters. *Lgr5*^*+*^ gene set^31^ enriched in late-regenerative cluster; spheroid fetal gene set^30^ in early-regenerative cluster. Full gene list and results for (**i**–**k**) in Supplementary Data 7. **l** CellChat^32^ ligand-receptor predictions, early and late clusters each versus all others: pro-regenerative signals enriched in the early cluster, pro-homeostatic in late (relative scores, no statistical test). Scale bar: 100 µm. Source data are provided as a Source Data file.

Several marker genes and transgenic lines have been used to study stem and progenitor populations in the homeostatic and regenerative gut^1^. We used the spatial transcriptomics cell-segmented data to measure the co-occurrence of homeostatic and regenerative gene expression within the segmented cells. At day 0, correlation matrices indicated that genes associated with the homeostatic stem cell state, notably *Olfm4*, *Lrig1*, *Ascl2*, and *Smoc2* were frequently co-expressed in the same cells (Fig. 3a and Supplementary Fig. 4a). By day 3 post-IR, a markedly altered correlation structure emerged, with regenerative markers such as *Clu*, *Anxa10* and *Msln*^26–28^ frequently co-occurring in cells distinct from those expressing homeostatic markers (Fig. 3a and Supplementary Fig. 4a). Based upon the bifurcation of the dendrogram in the day 3 post-IR correlation plot, we partitioned genes into homeostatic and regenerative marker sets. Direct visualization of these gene sets revealed largely non-overlapping expression patterns, with homeostatic genes predominating at days 0, 1 and 5, and regenerative genes at days 2 and 3 (Fig. 3b). Spatial quantification further showed regenerative gene signatures enriched toward the upper crypt, while homeostatic signatures remained more basal (Fig. 3b), consistent with BrdU labeling of proliferative cells between days 2–3 post-IR demonstrating that proliferative regeneration after tissue damage initiates in the upper crypt (Fig. 3c).

To further dissect the dynamics of regenerative cells in the crypt, we re-clustered the crypt epithelial cell populations using graph-based clustering and found two crypt subclusters that were absent at day 0 but emerged between days 2–4 post-irradiation (Fig. 3d). Time-course quantification of cells belonging to each subcluster showed that the upper subcluster was most abundant on day 2, while the lower crypt subcluster expanded beginning days 3–4 (Fig. 3e). Both populations coexist at days 3–4 and are replaced by a distinct crypt cluster by day 5. Based on their temporal emergence and spatial positioning, we designated these subclusters as the early and late regenerative populations, respectively. Regenerative marker genes such as *Areg*^29^ and *Clu*^26,27^ were highly enriched in the early regenerative, upper subcluster. Conversely, homeostatic markers, such as *Olfm4*, were enriched in the lower, late regenerative subcluster (Fig. 3f, g and Supplementary Fig. 4b). Spatial quantification confirmed that early regenerative cells occupy a higher crypt position relative to the late regenerative population (Fig. 3h), consistent with BrdU labeling showing regeneration initiating from upper-crypt progenitors (Fig. 3c).

To better appreciate the differences between these populations, differential expression analysis was performed at day 3 post-IR, comparing the early to late regenerative clusters. Early regenerative cells were enriched for fetal-like genes (*Clu*, *Anxa1*, *Anxa8*), whereas the late regenerative population expressed homeostatic, stem-associated genes (*Smoc2,* *Olfm4*, Fig. 3i). Gene ontology enrichment revealed functional distinctions consistent with these expression differences: the early regenerative population was enriched for immune and inflammatory processes and markers of differentiating cells, while the late population showed enrichment for DNA repair, replication, and other cell cycle-associated pathways (Fig. 3j and Supplementary Data 7). GSEA independently confirmed a relative enrichment of a fetal intestinal epithelial gene set^30^ in the early regenerative population and an *Lgr5*^*+*^-enriched gene set^31^ in the late regenerative population (Fig. 3k).

Finally, CellChat^32^ ligand-receptor predictions between the epithelial and stromal clusters at day 3 post-IR demonstrated that the early and late populations favor distinct signaling networks. Early regenerative cells were more receptive to known pro-regenerative signals (TGFβ, MAPK-activating ligands, and Collagen) while late regenerative cells showed more inclination to receive homeostatic stem cell signals such as WNT and NOTCH (Fig. 3l). These analyses collectively support a model in which early regenerative cells arise from more differentiated cells and exist in a higher inflammatory state in the upper crypt, while the late regenerative cells are more proliferative, less differentiated, lower in the crypt, and closer to restoring the homeostatic state.

### Spatiotemporal evidence of epithelial hypertranscription

**Fig. 4:**
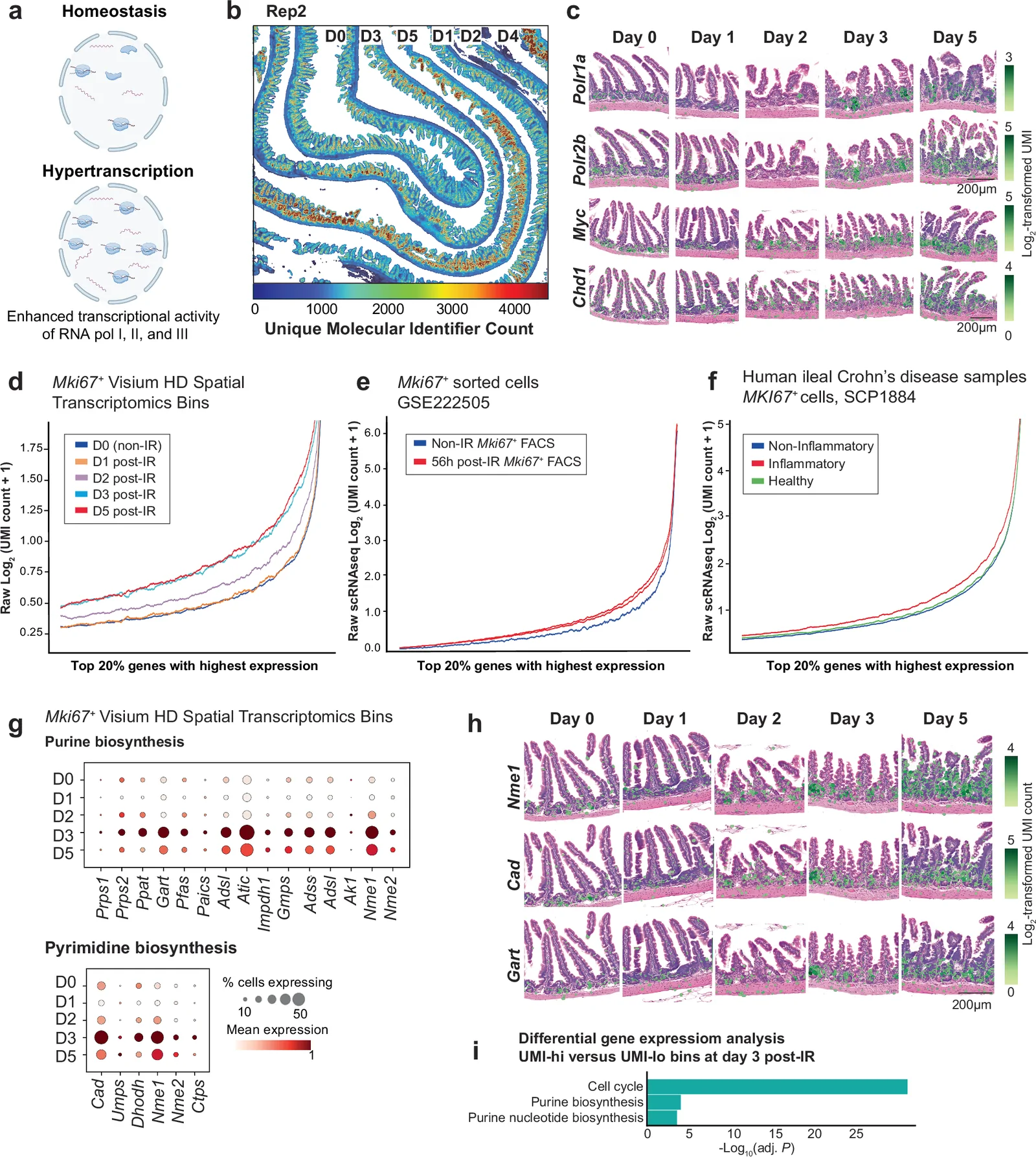
Global transcript levels are elevated in regenerating intestinal tissues, indicating hypertranscription is a feature of intestinal regeneration. **a** Enhanced activity of RNA polymerases I, II, and III marks hypertranscription in regenerating intestinal tissues. Created in BioRender. Ofer, R. (2026) https://BioRender.com/iogx188. **b** Heatmap of raw unique molecular identifier (UMI) counts demonstrates increased transcript abundance at days 3 and 5 post-IR, with elevated total UMI counts per barcode, rep2. **c** Spatial expression of RNA polymerase subunits shows upregulation of *Polr1a*, *Polr2b*, *Myc* and *Chd1* across regenerative tissues. Log_2_-transformed expression, rep2. **d**–**f** Regenerating intestinal tissues show global transcriptomic amplification in proliferative epithelial cells. Plots depict mean log_2_-transformed raw unique read counts of the top 20% most highly expressed genes. **d** Spatial transcriptomic data (rep2) filtered for proliferative epithelial bins (*Mki67*^+^ and either *Epcam*^*+*^ or *Vil1*^+^); **e** Ki67 FACS-sorted cells at 56 h post-IR compared to non-IR Ki67 single cell RNA-seq (Chen et al.^23^; GSE222505); **f** Human ileal Crohn’s disease samples (*MKI67*^*+*^; scRNA-seq; Kong et al.^42^). **g** Nucleotide metabolism genes are upregulated in proliferative epithelial bins (*Mki67*^*+*^;rep2) starting on day 3 post-IR. Normalized mean expression of key genes demonstrates increased de novo purine and pyrimidine biosynthesis to support regenerative demands. **h** Elevated expression of nucleotide synthesis genes (*Cad, Nme1, Gart*), localized to proliferative cell compartments. Visualized with cell segmentation, rep2. **i** GO enrichment analysis of genes upregulated in UMI-high bins at day 3 post-IR reveals enrichment of biosynthetic and RNA processing pathways. Bins were classified as UMI high ( ≥ 2500 UMI counts) or UMI low ( < 2500 UMI counts), differential expression was performed between the groups within each timepoint (log_2_FC > 1, MNA > 0.03, adj. *P* < 0.05). On day 3 post-IR: 177 genes enriched in UMI-high versus 427 in UMI-low bins. Full gene list and GO results in Supplementary Data 8. Scale bar: 200 µm. Source data are provided as a Source Data file.

Hypertranscription is the phenomenon in which cells elevate their transcriptional output, often associated with stem cell activity^33–36^ (Fig. 4a). Normalization methods in techniques such as RNA-seq and scRNA-seq usually equilibrate total mRNA counts between samples or cells, masking the detection of hypertranscription^34^. As part of quality control during spatial data analysis, we examined whether unique molecular identifier (UMI) count distributions were uniform across the capture area. While the UMI distributions passed quality control for an even distribution, we noticed an interesting pattern of elevated UMI counts localized to the regenerating epithelium, particularly at days 3–5 post-IR (Fig. 4b), an observation that was consistent across replicates (Supplementary Fig. 5a).

A hallmark of hypertranscription is the elevated expression of RNA polymerases^37^, which we observed in the regenerating epithelium (Fig. 4c). Other genes associated with hypertranscription, such as *Chd1*^38^ and *Myc*^38,39^, were similarly enriched in the regenerating epithelium (Fig. 4c). To determine whether the increase in transcript levels was due to the upregulation of specific genes or a genome-wide increase, we analyzed the spatial transcriptomics bins as pseudo-single-cell data. *Mki67*-expressing bins were extracted at each timepoint to allow direct comparison of proliferating cells across timepoints. Interestingly, raw UMI counts were elevated across most expressed genes in regenerative timepoints compared to non-IR controls (Fig. 4d and Supplementary Fig. 5b), consistent with hypertranscription. We sought additional evidence for hypertranscription using independent methodologies: scRNA-seq of cells sorted from Ki67-RFP knock-in mice^40^ of non-IR controls compared to IR tissues^23^ (Fig. 4e), and proliferating cells (*Mki67*^*+*^*)* that are either expressing the regenerative marker *Sca1* or not^41^ (Supplementary Fig. 5c). Both analyses showed a similar pattern: UMI count was elevated across most genes in the regenerating tissues compared to proliferating cells from homeostatic tissues. Next we investigated whether a similar phenomenon of hypertranscription could be occurring in human cells after damage. While human samples post-ionizing radiation were not available for investigation, we could interrogate data from Crohn’s patients’ epithelium^42,43^. Interestingly, in Crohn’s patient samples from ileal tissue, higher RNA counts were observed in inflamed tissue samples than in uninflamed patient samples or in healthy controls (Fig. 4f and Supplementary Fig. 5d), consistent with hypertranscription occurring following inflammatory damage in humans. Raw UMI count within the mouse spatial atlas varied across the post-IR time course: day 1 post-IR levels were reduced relative to day 0, while later post-IR timepoints showed consistently elevated counts across replicates (Supplementary Fig. 5e). Examination of UMI counts by cell type revealed that clusters such as smooth muscle, crypt stromal, Peyer’s patch, and enteric neuron populations displayed systematically lower UMI counts relative to epithelial populations (Supplementary Fig. 5f). Collectively, these findings demonstrate the utility of spatial transcriptomics for evaluating mRNA density across distinct tissue compartments.

Hypertranscription necessitates increased nucleic acid production for mRNA synthesis. Correspondingly, transcript levels for genes involved in de novo nucleotide metabolism, including both purine and pyrimidine synthesis pathways^44^, were upregulated in the proliferating regions of the regenerating tissues compared to non-IR controls (Fig. 4g–i and Supplementary Data 8). These observations were consistent across independent experiments (Supplementary Fig. 5g, h). Thus, as observed in various stem and progenitor populations^34^, spatial transcriptomics data revealed that the regenerating epithelium enters a state of hypertranscription, presumably to fuel the increased growth demands during restoration of the damaged tissue. Still, it cannot be discounted that the increases in nucleotide synthetic pathways may also be associated with the support for DNA replication.

### Increased signaling activity detected during regeneration

**Fig. 5:**
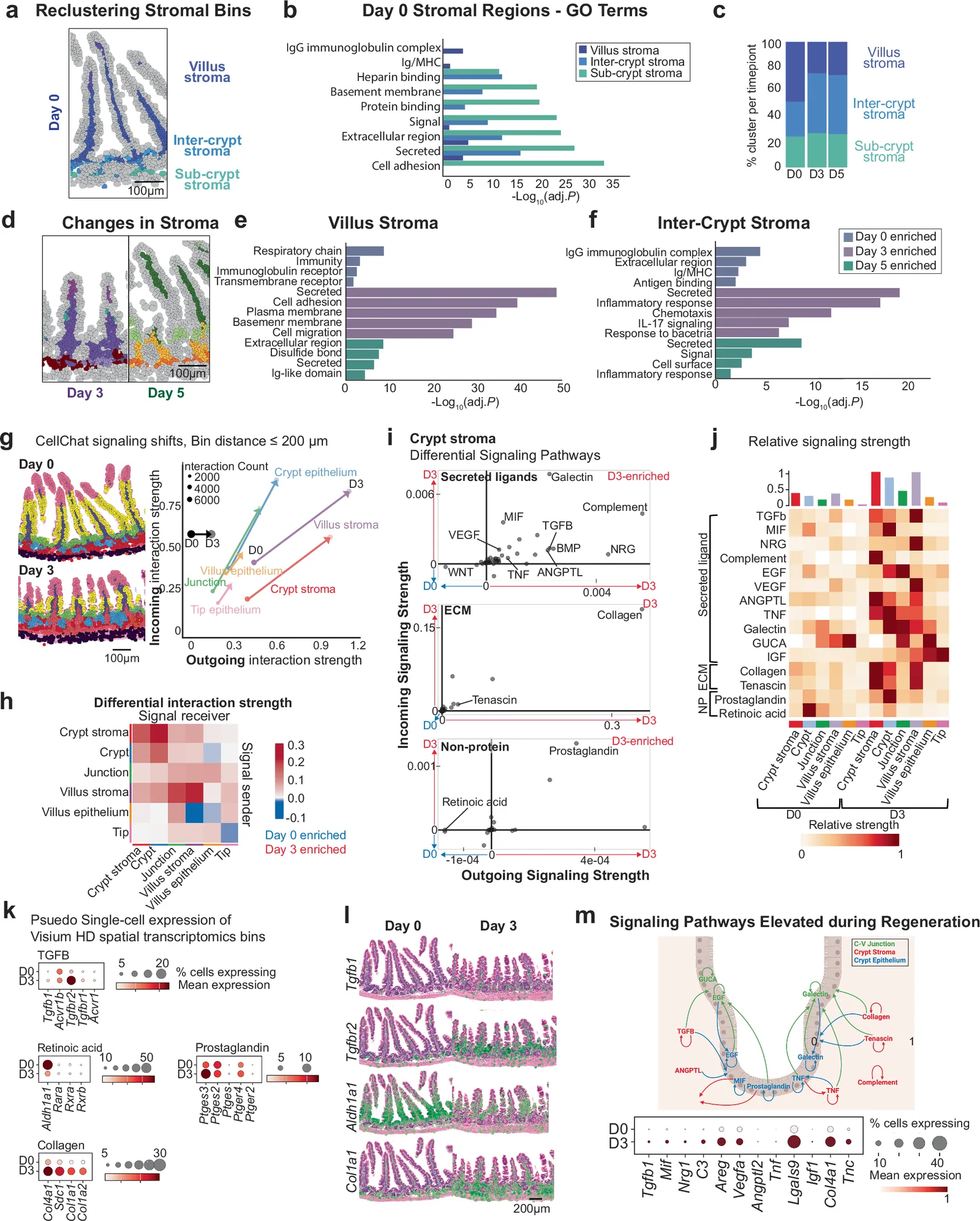
Spatial transcriptomics reveals regional signaling dynamics during regeneration and provides insight into stromal-epithelial interactions. **a** Stromal regions are defined by unsupervised graph-based clustering (rep2); villus stroma, inter-crypt stroma, and sub-crypt stroma. **b** GO enrichment in day 0 tissues reveals distinct biological functions for each stromal domain. Full gene list and GO results in Supplementary Data 9. **c** Proportional representation of the stromal regions across days 0, 3 and 5 post-IR. **d** Stromal domains identified at day 3 and day 5 post-IR by graph-based clustering used for GO term analyses in (**e**) and (**f**). Colors represent villus, inter-crypt, or sub-crypt stromal clusters. **e**, **f** GO term enrichment in villus stroma (**e**) and inter-crypt stroma (**f**) reveals upregulation of immune activation and tissue repair pathways at days 3 and 5 post-IR relative to day 0. Full gene list and GO results in Supplementary Data 10–12. **g** Spatial CellChat analysis comparing day 0 (D0) and day 3 (D3). Spatial domains defined by unsupervised clustering. Changes in outgoing and incoming signaling strength per region. Full results in Supplementary Data 13. **h**–**j** CellChat analysis; **h** Differential signaling strength map showing enhanced stromal signaling at D3 post-IR (red) versus D0 (blue), measured by incoming and outgoing activity. **i** Differential signaling activity in the crypt stroma between day 0 and day 3 post-IR, assessed across the Secreted Signaling, Non-protein and ECM-Receptor interaction databases of CellChatDB. Pathways enriched at day 3 are in the upper right quadrant. **j** Total signaling strength (incoming + outgoing) across stromal regions for selected regeneration-associated pathways. **k** Dot plots of normalized log_2_ expression levels of key ligands and receptors across day 0 and day 3 post-IR. **l** Log_2_ expression of selected TGFβ, collagen, and retinoic acid signaling genes in day 0 and day 3 tissues, indicating shifts in pathway activation during regeneration. **m** Spatially restricted CellChat predictions recapitulate known regeneration-associated signaling pathways, supported by corresponding changes in ligand and receptor gene expression shown in dot plots. Created in BioRender. Ofer, R. (2026) https://BioRender.com/ivwn6ot. Scale bar: 100 µm. Source data are provided as a Source Data file.

We explored whether spatial transcriptomics could be used to detect dynamic interactions between cells in distinct spatial domains. Graph-based unsupervised clustering divided the stromal compartments into three domains: the sub-crypt, inter-crypt, and villus stroma (Fig. 5a). Within the control tissues, differential gene expression and subsequent GO term analysis suggested distinct roles for each stromal region (Supplementary Data 9). The villus stroma was enriched for adaptive immune-related functions, including MHC-associated and IgG-related gene ontology terms (Fig. 5b), consistent with its role in antigen presentation and local antibody-mediated immune responses at the mucosal interface. In contrast, the crypt and sub-crypt stromal regions were enriched for structural and supportive components, such as collagen production and extracellular matrix organization, highlighting roles in providing mechanical support and modulating epithelial-stromal signaling (Fig. 5b). Quantification of bins across the time course revealed an expansion of the inter-crypt region post-IR, with nearly twice as much area associated with the inter-crypt stromal transcriptome compared to the non-IR state (Fig. 5c). GO analysis between control and post-IR timepoints demonstrated a shift in the villus stroma transcriptome (Fig. 5d, e and Supplementary Data 10). Genes elevated during the homeostatic state were associated with functions such as immunoglobulin complex and retinol metabolism signatures, while post-IR there was an increase in genes associated with an active inflammatory response, extracellular signaling, matrix remodeling, and cell adhesion pathways (Fig. 5e), indicative of immune activation and tissue repair processes. Similarly, the inter-crypt stroma transitioned from an adaptive immune signature at day 0, marked by antigen binding and MHC-related terms, to an activated state enriched for complement activation, chemotaxis, cytokine signaling, and innate immune pathways (Fig. 5f), consistent with a transition toward innate immune activation, inflammatory response, and tissue remodeling following injury (Supplementary Data 11, 12).

To more specifically interrogate signaling interactions within and between tissue compartments, we utilized CellChat^32^, an algorithm that leverages annotated datasets of ligand-receptor pairs to predict signaling interactions between cells. We used the spatial information in the data to limit potential interactions detected by CellChat^32^ to bins within 200 µm of each other. A summary of signaling interaction strength for each region at day 0 and day 3 post-IR revealed a general increase in signaling activity during regeneration, with the crypt and junctional epithelium and the crypt and villus stromal compartments being particularly more active during day 3 (Fig. 5g). By comparison, signaling activity at the villus tip and villus epithelium was similar between the IR and non-IR samples, a trend observed across the independent experiments (Supplementary Fig. 6a). Turning our focus to specific cluster-cluster interactions, it was apparent that the strongest gain in interactions at day 3 post-IR was occurring between the crypt stroma and crypt epithelium clusters (Fig. 5h). Of all the predicted active signaling pathways that are dynamic between day 0 and day 3 (Fig. 5i and Supplementary Fig. 6b), we decided to investigate those that have documented roles in regeneration after tissue damage, including transforming growth factor beta (TGFβ)^23,45^, macrophage migration Inhibitory factor (MIF)^46^, neuregulin-1 (NRG)^25,47^, the complement system^48,49^, guanylate cyclase C (GUCA)^50,51^, EGF^29,52,53^, vascular endothelial growth factor (VEGF)^54^, angiopoietin-like proteins (ANGPTL)^55^, insulin-like growth factor (IGF)^56^, tumor necrosis factor (TNF)^57^, galectin^58^, collagen^13^, tenascin^59^, prostaglandin E2^60,61^, and retinoic acid metabolism^62^ (Fig. 5j and Supplementary Fig. 6c). We had previously observed a role for TGFβ signaling during regeneration^23^ and RNAscope pinpointed *Tgfbr2* and *Tgfb1* expression: *Tgfbr2* was found to be elevated in the crypt and lower villus epithelium and throughout the stroma in day 3, whereas *Tgfb1* was elevated in the stroma at day 3. The spatial analysis confirmed our previous studies, showing similar changes in both *Tgfb* receptor and ligand expression (Fig. 5k and l and Supplementary Fig. 6d). CellChat also predicted signaling from *Tgfb1* produced in the villus and crypt stroma to be received in the crypt and junction epithelium (Fig. 5i, j and Supplementary Fig. 6b). Other signaling pathways upregulated during regeneration and their predicted source and target regions are summarized, such as MIF predominantly produced in the crypt epithelium signaling to the surrounding stroma (Fig. 5m). The results were reproducible across different replicates (Supplementary Fig. 6e, f), giving us confidence to explore more novel results of the CellChat analysis and highlight the utility of this spatial transcriptomics data as a resource for investigating the dynamic expression of genes across the time course of the intestinal damage-repair cycle. The easily accessible dataset should help support research across the field of intestinal regeneration (GSE303705; Supplementary Note 1).

## Discussion

Single-cell transcriptomics has transformed our ability to classify cell types and states, including when studying intestinal regeneration. However, scRNA-seq lacks spatial context, limiting insights into tissue organization and cell-cell interactions. The process of harvesting single cells may also compromise their biology. Traditional in situ hybridization techniques are required to validate single-cell findings and obtain spatial information; however, they have low throughput. The datasets generated in this study overcome these challenges by providing spatially resolved mRNA expression data for 16,921 genes. They replicate prior RNAscope results^23^ (Fig. 5l), and recapitulate known biology, including zonation of villus genes at homeostasis^17^ and upregulation of regenerative markers during repair^12,41^. We also confirm that signaling pathways previously implicated in regeneration exhibit expression of key ligands and receptors in relevant tissues^56,57^, further validating the approach as an alternative for RNA FISH.

While Visium HD does not achieve true single-cell resolution, the use of 2-µm cell-segmented bins combined with epithelial gene filtering minimizes stromal contamination, and we are confident the reported transcriptional differences largely reflect genuine epithelial changes. Despite these caveats, our dataset can help validate future studies and serve as a resource for new hypothesis testing. For example, we highlight distinct patterns in interferon-signaling pathway target genes (*Isg15*, Fig. 1i) and the capability for intra-tissue differential expression analysis (Fig. 1j). While we confirm known regeneration-associated pathways, we also identify several novel candidates using the spatial component of CellChat (Supplementary Data 13) that merit further investigation. These spatial transcriptomic datasets offer a time- and cost-efficient strategy to prioritize targets for future studies focused on regeneration, inflammation, and therapeutic discovery.

Spatial assays are rapidly gaining popularity due to their ability to preserve tissue context while enabling deep molecular insights^63,64^. Spatial proteomics allows mapping of protein expression, with resolutions ranging from single-protein detection to 20–50 µm resolution in mass spectrometry-based approaches^65^. Spatial metabolomics is also emerging as a powerful tool for mapping metabolite distribution at similar resolution (5–20 µm), providing complementary insights into cellular function and disease states. Recent advances, such as deep visual proteomics, integrate imaging, AI, and mass spectrometry to profile disease-relevant cells with high precision^66^. Multiscale approaches that combine targeted and exploratory methods are expanding both the depth and breadth of spatial data^65,67^. Future advances to integrate spatial-omics layers in the gut will help uncover disease mechanisms, inform therapeutic discovery, and advance precision medicine^64,68^.

## Methods

### Animals

All mouse protocols and experiments were approved by the Rutgers Institutional Animal Care and Use Committee (protocol #999900335) and complied with all relevant ethical regulations and ARRIVE guidelines, including efforts to reduce the total number of animals used. Mice were housed under a 12-h light/12-h dark cycle at 18–25 °C and 30–70% relative humidity. C57BL/6 J (JAX #000664) male littermates (8–12 weeks old) were used for spatial transcriptomics after whole-body ionizing radiation (IR) at 12 Gy using a caesium-137 irradiator. Following irradiation, mice were monitored daily for signs of radiation-induced morbidity, including body-weight loss, reduced activity, hunched posture and poor coat condition. The humane endpoint was defined as ≥20% loss of body weight, at which point animals would be euthanized; no animals reached this endpoint before their scheduled collection timepoint. Mice were euthanized by cervical dislocation immediately prior to tissue collection, which was approved and scientifically justified under IACUC protocol. Tissues were collected between 10:00 and 14:00.

### Tissue processing for Visium HD Spatial Transcriptomics

Jejunal tissues were washed with cold PBS and opened longitudinally. Tissues from different treatments were arranged together for co-embedding and fixed overnight with 4% paraformaldehyde at 4 °C. Tissues were dehydrated in ascending alcohols, processed with xylene, and embedded, generating a total of four formalin-fixed paraffin-embedded (FFPE) tissue blocks which were cut at 5-µm thickness. H&E staining and imaging were performed according to the Visium HD FFPE Tissue Preparation Handbook (CG000684) and guides (CG000518 revision C and CG000688). All tissue samples passed a DV200 quality check for RNA integrity. Imaging was done in the Olympus VS120 format.

### Visium HD spatial transcriptomics sequencing

Samples were processed using the Visium CytAssist platform and sequenced following the Visium HD Spatial Gene Expression Reagents Kits User Guide (CG000685). Libraries were prepared using the 10x Genomics Visium HD FFPE protocol, and sequencing was performed on an Illumina NovaSeq X Plus platform using paired-end 150-bp reads with dual indexing (10-bp index length). Libraries were sequenced on partial lanes with a 5% PhiX control spike-in for run quality calibration. Sequencing data were demultiplexed by the provider. Total sequencing output per replicate and bin count per timepoint can be found in Supplementary Fig. 1b–d.

### Visium HD spatial gene expression analysis

Data were processed using 10x Genomics Space Ranger (v3.1.3 and v4.0), outputting data binned at 2-µm and 8-µm resolution. Unless otherwise noted, all analyses were performed using the 2-µm bin-based cell-segmented data from Replicate 2 (rep2), selected as the representative high-depth replicate to enable cell-level resolution. Exceptions include analysis of reps 1, 3 and 4, and visualization of *k*-means clustering of epithelial zonation, which shows 8-µm bin spatial segmentation (Fig. 2b). In the cell-segmented data, each barcode represents a single cell. In the 8-µm binned data, each barcode represents an 8 × 8 µm square composed of sixteen 2-µm bins. Replicates 1, 3, and 4 were acquired at standard sequencing depth and analyzed using 8 µm bin spatial bins; spatial patterns and cluster identities across these replicates were highly concordant with those from the cell-segmented rep2, confirming that findings are robust across both segmentation strategies and sequencing depths.

### Cell segmentation

Nuclear and cellular segmentation was conducted using 10x Genomics Space Ranger (v4.0) and visualized on 10x Genomics Loupe Browser (v9.0). This method partitions barcodes into bins based on automated nuclei detection in the corresponding image, matching H&E-stained microscopy images to associate spatial transcriptomic data with the corresponding cells.

### Clustering and differential gene expression

Graph-based and *k*-means clustering were performed within Loupe Browser (v8.0). In some cases, sub-clustering of specific tissue regions was performed using the “reanalyze” feature (Figs. 3, 5). Differential gene expression and GO term enrichment were performed as described in “Statistics and reproducibility” (Methods); filtering criteria are given per analysis in the relevant figure legends. For epithelial-specific analysis (Fig. 2), unsupervised *k*-means clustering identified five distinct clusters. Stromal bins, identified through graph-based clustering, were excluded prior to downstream analysis. Differential gene expression (DEG) analysis was conducted within the day 0 dataset to compare the five epithelial clusters or compare between day 0 and day 3 post-IR. Heatmaps were generated using Morpheus to visualize normalized transcript levels of genes of interest (https://software.broadinstitute.org/morpheus/).

For crypt-epithelium analysis (Fig. 3), the “reanalyze” feature was used to generate subclusters from the crypt epithelium and Paneth cell graph-based clusters. VolcaNoseR was used to generate volcano plots and identify transcripts enriched in the ‘early’ versus ‘late’ subclusters (https://huygens.science.uva.nl/VolcaNoseR/). Differential expression analysis was restricted to genes overlapping an epithelial-enriched gene list (Supplementary Data 4) generated from bulk RNA-seq datasets of mouse intestinal epithelium, retaining genes with FPKM > 1 in all crypt or villus epithelial samples. To account for the possibility that injury induces expression of genes absent from homeostatic epithelium, we supplemented this list with genes from published intestinal injury and regeneration datasets of isolated epithelial cell populations, including irradiation-induced epithelial signatures, revival stem cell programs, and fetal reversion gene sets^12,13,17,23,30,31,69^ ( ~ 11,000 genes total). For epithelial-specific analyses, only genes present in the epithelial-enriched list were retained. For stromal-specific analyses (Fig. 5), stromal clusters were first identified using graph-based clustering. To resolve stromal heterogeneity the “reanalyze” feature was used to define three sub-stromal clusters: villus stroma, inter-crypt stroma, and sub-crypt stroma. Equivalent subclusters were identified across timepoints, and differential gene expression analysis was performed. To focus on stromal expression, epithelial-enriched genes were removed prior to analysis.

### Gene set enrichment analysis^22^ (GSEA)

Performed with GSEA v4.3.3 (https://www.gsea-msigdb.org/gsea/index.jsp); enrichment significance was assessed by the GSEA permutation test and reported as the normalized enrichment score (NES) and false discovery rate (FDR). To assess villus epithelial zonation, differential gene expression between day 0 and day 3 post-IR epithelial clusters was used as a ranked gene list (MNA > 0.05) and run against villus zone gene sets derived from Moor et al.^17^. To characterize interferon-responsive cells, *Ifit1*^*+*^ and *Ifit1*^*−*^ cells were contrasted to generate a ranked gene list (MNA > 0.05) and run against the *Ifit1* gene set reported in Constant et al.^19^. To validate the transcriptomic identity of fetal-like and stem-like regenerative crypt subclusters, differential expression between these populations was run against a fetal-like spheroid intestinal gene set^30^ and an *Lgr5*^*+*^ stem cell gene signature^31^. The gene signatures used in this study are shown in Supplementary Data 4.

### BrdU labeling (immunohistochemistry)

Mice (C57BL/6 J, JAX# 000664; male, 10 weeks; *n* = 3) were injected with 1 mg BrdU at the indicated timepoints, and intestinal tissues were collected 2 *h* later, fixed overnight in 4% paraformaldehyde at 4 °C, dehydrated through ascending alcohols, processed with xylene and paraffin-embedded; 5-µm sections were used. BrdU was detected with rat anti-BrdU (Bio-Rad, Cat# MCA2060, RRID:AB_323427; rat monoclonal [BU1/75], 1:500), followed by a biotinylated rabbit anti-rat IgG (H + L) secondary (Vector Laboratories, Cat# BA-4001, RRID:AB_10015300, 1:200) and the Vectastain ABC HRP kit (Vector Laboratories, Cat# PK-6105). Slides were developed with 0.05% DAB (Amresco 0430) and 0.015% hydrogen peroxide in 0.1 M Tris, counterstained with haematoxylin, and imaged by brightfield microscopy (Nikon Eclipse E800; Lumenera INFINITY3 camera, Infinity Capture v6.5.6).

### EdU labeling and Ki67 immunofluorescence

Mice (C57BL/6 J; male and female, 10 weeks; *n* = 4; 2 control and 2 irradiated) were injected intraperitoneally with 200 µg EdU; tissues were paraffin-embedded and sectioned as above. EdU was detected by click chemistry (Invitrogen Click-iT EdU Cell Proliferation Kit for Imaging, Alexa Fluor 488, C10337). For Ki67, heat-induced epitope retrieval was performed in pH 6 citrate buffer; sections were blocked in 3% BSA/PBS and incubated overnight at 4 °C with anti-Ki67 (Abcam, Cat# ab16667, RRID:AB_302459; rabbit monoclonal [SP6], 1:400), followed by goat anti-rabbit IgG (H + L) Alexa Fluor 555 (Invitrogen, Cat# A-21428, RRID:AB_2535849, 1:1000). Nuclei were counterstained with DAPI (Biotium 40043). Imaging used a Leica THUNDER microscope with LAS X.

### Gene correlation plot (Fig. 3)

Epithelial cells were subset by *Epcam* or *Vil1* expression, and a cell-by-gene matrix was then generated containing previously described homeostatic and regenerative stem cell markers. Pearson correlation coefficients were computed using pandas.DataFrame.corr(), and clustered heatmaps with corresponding dendrograms were visualized using seaborn.clustermap(). The following genes were included in the analysis: *Ascl2, Lgr5, Olfm4, Smoc2, Hmga2, Top2a, Msln, Lrig1, Tert, Hopx, Bmi1, Ly6d, Tacstd2, Anxa1, Anxa8, Anxa10, Spp1, Clu, Areg, Ereg* and *Fgfbp1*.

### Gene list scoring and distance measurement

Based on the dendrogram from the correlation analysis, genes were partitioned into homeostatic (*Tert, Hmga2, Bmi1, Lgr5, Ascl2, Olfm4, Smoc2, Lrig1, Top2a, Hopx* and *Fgfbp1)* and regenerative (*Spp1, Ly6a, Tacstd2, Anxa1, Anxa8, Msln, Clu, Ereg, Ly6d, Anxa10* and *Areg*) panels. scanpy.tl.score_genes was used to compute a per-cell expression score for each panel, calculated as the average expression of the gene list minus the average expression of a randomly sampled reference list of 150 genes across 1000 bins. To quantify each cell’s distance to the smooth muscle layer, a cKDTree was constructed from the smooth muscle cluster coordinates, and the nearest neighbor distance was queried for every cell. Epithelial cells’ gene-panel scores were averaged within 10 µm intervals up to 300 µm and plotted as a function of distance from the smooth muscle layer. Cell type scores and read counts per cell by distance were generated using the same binning strategy. The same approach was applied to quantify villus epithelial zonation gene set scores along the crypt-villus axis at days 0, 3 and 5 post-IR (Supplementary Fig. 3b), with values representing the average score within 10 µm distance windows and SEM shown as shading.

### Raw counts scatterplot analysis

To assess hypertranscription, 10x Genomics Space Ranger output bins were filtered in Scanpy^70^ for expression of *Mki67* and either *Vil1* or *Epcam*, isolating proliferative epithelial cells only. Average gene raw counts were calculated, log_2_-transformed, and sorted by mean expression across samples, shown as a line representing the rolling mean of 100 genes. To compare mRNA abundance between regenerative and homeostatic states, scRNA-seq datasets from Chen et al.^23^ (GSE222505; KI67 FACS-sorted, 56 h post-IR) and Nusse et al.^41^ (GSE108233; *Sca-1*^*+*^ and *Sca-1*^*−*^ crypt cells from *H. polygyrus*-infected mice) were analyzed. The *Sca-1* dataset was further filtered for *Mki67*^*+*^ expression. For human samples, scRNA-seq datasets of Crohn’s disease epithelial cells from ileal biopsies were analyzed. Inflamed tissues were compared with healthy and non-inflamed tissues using data from 82 patients in Li et al. (GSE266546)^43^ and 48 patients in Kong et al. (SCP1884)^42^. Dot plots of gene-specific expression were created using Scanpy^70^ with normalized counts. Spatial bins required a minimum of 40 transcripts and 10 genes; scRNA-seq cells required 1000 transcripts and 100 genes.

### Cell-cell signaling analysis

CellChat^32^ v2 (2.1.2) was used to analyze bin-to-bin communications from 2-µm Space Ranger output. Bins with ≥40 unique molecular identifiers (UMIs) were selected by treatment timepoint and cell type region, as identified by graph-based clustering, and exported to Seurat. Normalized counts and spatial coordinates were used as input to a CellChat object^71^. Analyses used the Secreted Signaling, ECM-Receptor, Cell-Cell Contact, and Non-Protein databases of CellChatDB. Communication probabilities and strengths between regions were predicted with computeCommunProb, with a truncated mean trim = 0.001, interaction range limited to 200-µm, and contact range limited to 20-µm. The statistical significance of each inferred interaction was assessed by a permutation test (random permutation of cell-group labels), with significant interactions defined at *P* < 0.05. A minimum of 10 cells per group was required to detect signaling, and computeCommunProbPathway identified the pathways involved. For differential comparisons, day 0 and day 3 workflows were analyzed separately, then merged to compare differentially enriched signaling-pathway communication strengths. Relative communication strengths were visualized with ComplexHeatmap^72^.

### Statistical analysis

*P-*values were adjusted using the Benjamini–Hochberg correction for multiple comparisons (Loupe Browser v8.0 and v9.0). Fold change was calculated as the ratio of normalized mean gene UMI counts in each cluster relative to all others. For Supplementary Fig. 2c, villi at each timepoint and replicate were scored as *Isg15*^*+*^ or *Isg15*^*−*^ and converted to binary strings, with villus units representing distance; the normalized Ripley’s L-function and 95% confidence intervals from 1000 Monte Carlo simulations were then calculated and plotted.

### Statistics & reproducibility

No statistical method was used to predetermine sample size; sample sizes were based on experimental feasibility and are consistent with standard practice in the field. Visium HD spatial transcriptomics was performed on 23 C57BL/6 J mice across four independent capture areas (biological replicates 1–4), each co-embedding tissues from multiple animals spanning four to six timepoints and including a day 0 control. By timepoint, *n* = 5 (day 0), 3 (day 1), 4 (day 2), 3 (day 3), 4 (day 4), 3 (day 5) and 1 (day 6); per-replicate per-timepoint animal numbers are provided in Supplementary Fig. 1a. Including a control timepoint within each capture area eliminated the need for batch correction across runs. Capture areas 1 and 4 (rep1, rep4) were prepared on independent days, and capture areas 2 and 3 (rep2, rep3) were prepared simultaneously on the same Visium HD run. Primary analyses were performed on capture area 2 (high sequencing depth) and subsequently repeated on the remaining capture areas to validate key findings. C57BL/6 J mice (JAX #000664) were used for all experiments. The spatial transcriptomics atlas used male mice aged 8–12 weeks. BrdU pulse-chase experiments used male mice aged 10 weeks (*n* = 3 biological replicates). EdU pulse-chase experiments used male and female mice aged 10 weeks (*n* = 2 control and *n* = 2 irradiated mice).

Sex was not considered as a variable in the primary study design. The spatial transcriptomics atlas used male mice exclusively to reduce variability, particularly given that Visium HD is a newly established assay. EdU pulse-chase experiments included both male and female mice; data were not disaggregated by sex.

No animals were excluded from the study. For Visium HD analyses requiring sufficient bin counts (for example, differential expression), rep2 and rep3 D4 samples had low bin count and were excluded from those specific analyses, whereas the two D4 timepoints in rep4 were retained and reproduced each other’s spatial patterning. A minimum expression threshold (detection in ≥ 50 cells across the dataset) was applied for gene-detection reporting.

The experiments were not randomized. Mice were allocated to irradiation or control conditions by experimental timepoint; formal randomization was not performed, as allocation was determined by the experimental design rather than by individual animal characteristics. The Investigators were not blinded to allocation during experiments and outcome assessment. Blinding during sample collection was impractical given the defined-timepoint irradiation design, and computational analyses used pre-established pipelines and objective criteria. Reproducibility of the spatial transcriptomics findings was supported by the concordance of spatial patterns and cluster identities across all four capture areas, which spanned standard and high sequencing depth and both 8 µm binned and cell-segmented data. BrdU and EdU histological experiments were independently replicated across the sample sizes indicated above.

Representative images are shown for the following experiments, each repeated with consistent results: H&E staining (Fig. 1c and Supplementary Fig. 1a), *n* = 4 capture areas; BrdU labeling (Fig. 3c), *n* = 3 mice; EdU/Ki67 labeling (Fig. 2g), *n* = 4 mice.

Human single-cell data were obtained from previously published studies (Li et al.^43^, GSE266546; Kong et al.^42^, SCP1884); no new human samples were collected for this study.

Differential expression and GO enrichment: differential expression between the indicated clusters was performed in Loupe Browser (v8.0 and v9.0) using a two-sided negative binomial-based test, with *P-*values adjusted by the Benjamini–Hochberg procedure. Resulting genes were filtered (criteria listed per analysis in the relevant figure legends) and analyzed by Gene Ontology (GO) term enrichment using DAVID Bioinformatics Resources^73,74^. GO statistical significance was assessed with a modified Fisher’s exact test (EASE score), a one-tailed test for over-representation, with Benjamini–Hochberg correction for multiple comparisons. Only GO terms with a Benjamini-adjusted *P* < 0.05 are shown; full results are provided in Supplementary Data. Bar length represents −log₁₀(Benjamini-adjusted *P-*value).

Volcano plot (Fig. 3i): Differential expression was computed in Loupe Browser using a two-sided negative binomial-based test, with *P-*values adjusted for multiple comparisons by the Benjamini–Hochberg procedure. Volcano plots were generated with VolcaNoseR (visualization only; no statistical test performed by the tool).

Fold change and spatial statistics: Fold change was calculated as the ratio of normalized mean gene UMI counts in each cluster relative to all others. For Supplementary Fig. 2c, villi at each timepoint and replicate were scored as *Isg15*⁺ or *Isg15*⁻ and converted to binary strings, with villus units representing distance; the normalized Ripley’s *L*-function and 95% confidence intervals from 1000 Monte Carlo simulations were then calculated and plotted.

### Reporting summary

Further information on research design is available in the Nature Portfolio Reporting Summary linked to this article.

## Supplementary information

Supplementary Information

Description of Additional Supplementary Files

Supplementary Data 1

Supplementary Data 2

Supplementary Data 3

Supplementary Data 4

Supplementary Data 5

Supplementary Data 6

Supplementary Data 7

Supplementary Data 8

Supplementary Data 9

Supplementary Data 10

Supplementary Data 11

Supplementary Data 12

Supplementary Data 13

Reporting Summary

Transparent Peer Review file

## Source data

Source Data

## Data Availability

The Visium HD spatial transcriptomics raw and processed data generated in this study have been deposited in the Gene Expression Omnibus (GEO) database under accession code GSE303705 [https://www.ncbi.nlm.nih.gov/geo/query/acc.cgi?acc=GSE303705]. The differential expression and gene ontology (DAVID) results generated in this study are provided as Supplementary Data. A step-by-step guide for uploading the spatial transcriptomics datasets generated in this study to the Loupe Browser, including dataset annotation, is provided as a Supplementary Note 1. The human single-cell data used in this study are available in the Single-Cell Portal (SCP) under accession code SCP1884^42^ [https://singlecell.broadinstitute.org/single_cell/study/SCP1884/human-cd-atlas-study-between-colon-and-terminal-ileum] and in the GEO database under accession code GSE266546^43^ [https://www.ncbi.nlm.nih.gov/geo/query/acc.cgi?acc=GSE266546]. Source data are provided in this paper.

## Code availability

The code used for the analysis is available at GitHub (https://github.com/tht38/intestinalspatialregen), and have been deposited at Zenodo (https://zenodo.org/records/21348331)^75^.
