# Supplementary material for: A spatial and temporal transcriptomic atlas of mouse intestinal regeneration in the jejunum
Source: Nat Commun. 2026 Sep 3;17:10484. doi: 10.1038/s41467-026-77292-8 (PMC13635233; doi:10.1038/s41467-026-77292-8)
Supplement: Supplementary file 1 [file 41467_2026_77292_MOESM16_ESM.pdf]

nature portfolio

Corresponding author(s): Michael Verzi

Last updated by author(s): Jul 10, 2026

Reporting Summary

Nature Portfolio wishes to improve the reproducibility of the work that we publish. This form provides structure for consistency and transparency in reporting. For further information on Nature Portfolio policies, see our Editorial Policies and the Editorial Policy Checklist.

Statistics

For all statistical analyses, confirm that the following items are present in the figure legend, table legend, main text, or Methods section.

n/a	Confirmed	
☐	☒	The exact sample size (*n*) for each experimental group/condition, given as a discrete number and unit of measurement
☐	☒	A statement on whether measurements were taken from distinct samples or whether the same sample was measured repeatedly
☐	☒	The statistical test(s) used AND whether they are one- or two-sided<br>*Only common tests should be described solely by name; describe more complex techniques in the Methods section.*
☒	☐	A description of all covariates tested
☐	☒	A description of any assumptions or corrections, such as tests of normality and adjustment for multiple comparisons
☐	☒	A full description of the statistical parameters including central tendency (e.g. means) or other basic estimates (e.g. regression coefficient) AND variation (e.g. standard deviation) or associated estimates of uncertainty (e.g. confidence intervals)
☐	☒	For null hypothesis testing, the test statistic (e.g. *F*, *t*, *r*) with confidence intervals, effect sizes, degrees of freedom and *P* value noted<br>*Give P values as exact values whenever suitable.*
☒	☐	For Bayesian analysis, information on the choice of priors and Markov chain Monte Carlo settings
☒	☐	For hierarchical and complex designs, identification of the appropriate level for tests and full reporting of outcomes
☐	☒	Estimates of effect sizes (e.g. Cohen's *d*, Pearson's *r*), indicating how they were calculated

Our web collection on statistics for biologists contains articles on many of the points above.

Software and code

Policy information about availability of computer code

Data collection	Software and packages include Space Ranger (v3.1.3 and v4.0), Loupe Browser (v8.0 and v9.0), R 4.4.3, Seurat 5.3.1.9999, Python 3.13.2, Scanpy 1.11.2, CellChat v2 version 2.1.2, pandas 2.2.3, numpy 2.1.3, matplotlib 3.10.1
Data analysis	Data was analyzed using 10x Genomics analysis pipelines which are all publicly available, including Space Ranger (v.3.1.3 and v4.0) and Loupe Browser (v8.0 and v9.0). Software R 4.4.3, Seurat 5.3.1.9999, Python 3.13.2, Scanpy 1.11.2, CellChat v2 version 2.1.2, pandas 2.2.3, numpy 2.1.3, and matplotlib 3.10.1 were used for downstream analysis.<br>Code usage and custom code are available here: https://github.com/tht38/intestinalspatialregen<br>For enrichment analysis we used GSEA: https://www.gsea-msigdb.org/gsea/msigdb/mouse/genesets.jsp<br>For differential expression analysis we used DAVID: https://davidbioinformatics.nih.gov/workspace.html<br>Morpheus was used to visualize normalized transcript levels of genes of interest (https://software.broadinstitute.org/morpheus/).<br>VolcaNoseR was used to generate volcano plots and identify transcripts enriched in the 'early' versus 'late' subclusters (https://huygens.science.uva.nl/VolcaNoseR/).<br>Details are outlined in the Methods section.

For manuscripts utilizing custom algorithms or software that are central to the research but not yet described in published literature, software must be made available to editors and reviewers. We strongly encourage code deposition in a community repository (e.g. GitHub). See the Nature Portfolio guidelines for submitting code & software for further information.

Data

Policy information about availability of data

All manuscripts must include a data availability statement. This statement should provide the following information, where applicable:

- Accession codes, unique identifiers, or web links for publicly available datasets
- A description of any restrictions on data availability
- For clinical datasets or third party data, please ensure that the statement adheres to our policy

The Visium HD spatial transcriptomics data (raw and processed) generated in this study have been deposited in the Gene Expression Omnibus (GEO) database under accession code GSE303705 (https://www.ncbi.nlm.nih.gov/geo/query/acc.cgi?acc=GSE303705). The differential expression and gene ontology (DAVID) analysis results generated in this study are provided in the Supplementary Information. The human single-cell data used in this study are available in the Single-Cell Portal (SCP) under accession code SCP1884 (https://singlecell.broadinstitute.org/single_cell/study/SCP1884/human-cd-atlas-study-between-colon-and-terminal-ileum) and in the GEO database under accession code GSE266546 (https://www.ncbi.nlm.nih.gov/geo/query/acc.cgi?acc=GSE266546). A step-by-step guide for downloading and opening the Loupe Browser files is provided as a Supplementary document ("Spatial_data_guide").

Research involving human participants, their data, or biological material

Policy information about studies with human participants or human data. See also policy information about sex, gender (identity/presentation), and sexual orientation and race, ethnicity and racism.

Reporting on sex and gender	*Use the terms sex (biological attribute) and gender (shaped by social and cultural circumstances) carefully in order to avoid confusing both terms. Indicate if findings apply to only one sex or gender; describe whether sex and gender were considered in study design; whether sex and/or gender was determined based on self-reporting or assigned and methods used. Provide in the source data disaggregated sex and gender data, where this information has been collected, and if consent has been obtained for sharing of individual-level data; provide overall numbers in this Reporting Summary. Please state if this information has not been collected. Report sex- and gender-based analyses where performed, justify reasons for lack of sex- and gender-based analysis.*
Reporting on race, ethnicity, or other socially relevant groupings	*Please specify the socially constructed or socially relevant categorization variable(s) used in your manuscript and explain why they were used. Please note that such variables should not be used as proxies for other socially constructed/relevant variables (for example, race or ethnicity should not be used as a proxy for socioeconomic status). Provide clear definitions of the relevant terms used, how they were provided (by the participants/respondents, the researchers, or third parties), and the method(s) used to classify people into the different categories (e.g. self-report, census or administrative data, social media data, etc.) Please provide details about how you controlled for confounding variables in your analyses.*
Population characteristics	*Describe the covariate-relevant population characteristics of the human research participants (e.g. age, genotypic information, past and current diagnosis and treatment categories). If you filled out the behavioural & social sciences study design questions and have nothing to add here, write "See above."*
Recruitment	*Describe how participants were recruited. Outline any potential self-selection bias or other biases that may be present and how these are likely to impact results.*
Ethics oversight	*Identify the organization(s) that approved the study protocol.*

Note that full information on the approval of the study protocol must also be provided in the manuscript.

Field-specific reporting

Please select the one below that is the best fit for your research. If you are not sure, read the appropriate sections before making your selection.

☒ Life sciences ☐ Behavioural & social sciences ☐ Ecological, evolutionary & environmental sciences

For a reference copy of the document with all sections, see nature.com/documents/nr-reporting-summary-flat.pdf

Life sciences study design

All studies must disclose on these points even when the disclosure is negative.

Sample size	No formal sample-size calculation was performed; sample sizes were determined based on experimental feasibility. Visium HD spatial transcriptomics was performed across four independent capture areas comprising 23 mice in total (day 0, n = 5; day 1, n = 3; day 2, n = 4; day 3, n = 3; day 4, n = 4; day 5, n = 3; day 6, n = 1). Each capture area contained tissue from different animals (Rep1, n = 5; Rep2, n = 6; Rep3, n = 6; Rep4, n = 6). BrdU pulse-chase experiments used n = 3 biological replicates (male mice). EdU pulse-chase experiments used n = 2 control and n = 2 irradiated mice (male and female).
Data exclusions	No animals were excluded from the study. For Visium HD analyses requiring sufficient bin counts (e.g. differential expression), day-4 replicate data of low bin count were excluded from those specific analyses. For gene-detection reporting, a minimum expression threshold was applied, requiring detection in at least 50 cells across the dataset.

Replication	Visium HD spatial transcriptomics was replicated across four capture areas, each containing tissue sections from 4–6 timepoints, including a day 0 control. Including a control and multiple timepoints within each capture area eliminated the need for batch correction across runs. Capture areas 1 and 4 were prepared on independent days; capture areas 2 and 3 were prepared simultaneously on the same Visium HD slide. Primary analyses were performed on capture area 2 and subsequently repeated on the remaining capture areas to validate key findings. Spatial patterns and cluster identities were highly concordant across replicates, confirming reproducibility of the primary findings. The day-4 samples in capture areas 2 and 3 had low bin counts and were excluded from analyses requiring high bin counts (e.g. differential expression), whereas the two day-4 timepoints in capture area 4 were retained and reproduced each other's spatial patterning. BrdU and EdU histological experiments were independently replicated across the indicated sample sizes (BrdU, n = 3; EdU, n = 4).
Randomization	Mice were allocated to irradiation or control groups based on experimental timepoint. Formal randomization was not performed; group allocation was determined by experimental design (timepoint) rather than by individual animal characteristics.
Blinding	Blinding was not performed. The experimental design (irradiation injury with defined timepoints) made blinding during sample collection impractical, and computational analyses were performed using pre-established pipelines and objective criteria.

Reporting for specific materials, systems and methods

We require information from authors about some types of materials, experimental systems and methods used in many studies. Here, indicate whether each material, system or method listed is relevant to your study. If you are not sure if a list item applies to your research, read the appropriate section before selecting a response.

Materials & experimental systems

n/a	Involved in the study	
☐	☒	Antibodies
☒	☐	Eukaryotic cell lines
☒	☐	Palaeontology and archaeology
☐	☒	Animals and other organisms
☒	☐	Clinical data
☒	☐	Dual use research of concern
☒	☐	Plants

Methods

n/a	Involved in the study	
☒	☐	ChIP-seq
☒	☐	Flow cytometry
☒	☐	MRI-based neuroimaging

Antibodies

Antibodies used	Antibodies and detection reagents: anti-BrdU (Bio-Rad, Cat# MCA2060, RRID:AB_323427; rat monoclonal [BU1/75], 1:500); anti-Ki67 (Abcam, Cat# ab16667, RRID:AB_302459; rabbit monoclonal [SP6], 1:400); biotinylated rabbit anti-rat IgG (H+L) (Vector Laboratories, Cat# BA-4001, RRID:AB_10015300, 1:200); goat anti-rabbit IgG (H+L) cross-adsorbed, Alexa Fluor 555 (Invitrogen, Cat# A-21428, RRID:AB_2535849, 1:1,000). BrdU was detected with the biotinylated anti-rat secondary and the Vectastain ABC HRP kit (Vector Laboratories, Cat# PK-6105), developed with 0.05% DAB and 0.015% H_2O_2 in 0.1 M Tris. EdU was detected by click chemistry (Click-iT EdU Cell Proliferation Kit for Imaging, Alexa Fluor 488; Invitrogen, C10337).
Validation	Anti-BrdU (Bio-Rad, Cat# MCA2060, RRID:AB_323427; rat monoclonal, clone BU1/75). Validated by the manufacturer for detection of BrdU incorporation by immunohistochemistry, and previously used for BrdU detection in mouse intestinal epithelium by Chen et al. (2023). In this study, signal was restricted to proliferative crypt cells in BrdU-pulsed tissue (Fig. 3c).<br>Anti-Ki67 (Abcam, Cat# ab16667, RRID:AB_302459; rabbit monoclonal, clone SP6). Validated by the manufacturer for Ki67 detection by immunohistochemistry and immunofluorescence in mouse tissue, and previously used as a proliferation marker in mouse intestinal epithelium by Chen et al. (2023). In this study, staining was restricted to the expected proliferative compartment (crypt base/transit-amplifying zone; Fig. 2g).

Animals and other research organisms

Policy information about studies involving animals; ARRIVE guidelines recommended for reporting animal research, and Sex and Gender in Research

Laboratory animals	Mus musculus, C57BL6/6J (JAX#000664), age 8-12 weeks
Wild animals	*Provide details on animals observed in or captured in the field; report species and age where possible. Describe how animals were caught and transported and what happened to captive animals after the study (if killed, explain why and describe method; if released, say where and when) OR state that the study did not involve wild animals.*
Reporting on sex	Sex was not considered in the study design. Visium HD spatial transcriptomics experiments used male mice exclusively. EdU pulse-chase experiments included both male and female mice, but data were not disaggregated by sex.
Field-collected samples	*For laboratory work with field-collected samples, describe all relevant parameters such as housing, maintenance, temperature, photoperiod and end-of-experiment protocol OR state that the study did not involve samples collected from the field.*
Ethics oversight	All animal experiments were conducted in accordance with protocols approved by the Rutgers University Institutional Animal Care and Use Committee (IACUC protocol #999900335). Mice were housed under a 12 h light/12 h dark cycle (automatic timer) at 18–25 °

C and 30–70% relative humidity, with 10–15 air changes per hour. Following irradiation, mice were monitored daily for signs of radiation-induced morbidity; the humane endpoint was defined as ≥20% loss of body weight, at which point animals would be euthanized. No animals reached this endpoint before their scheduled collection timepoint. Mice were euthanized by cervical dislocation immediately prior to tissue collection.

Note that full information on the approval of the study protocol must also be provided in the manuscript.

Plants

Seed stocks

Report on the source of all seed stocks or other plant material used. If applicable, state the seed stock centre and catalogue number. If plant specimens were collected from the field, describe the collection location, date and sampling procedures.

Novel plant genotypes

Describe the methods by which all novel plant genotypes were produced. This includes those generated by transgenic approaches, gene editing, chemical/radiation-based mutagenesis and hybridization. For transgenic lines, describe the transformation method, the number of independent lines analyzed and the generation upon which experiments were performed. For gene-edited lines, describe the editor used, the endogenous sequence targeted for editing, the targeting guide RNA sequence (if applicable) and how the editor was applied.

Authentication

Describe any authentication procedures for each seed stock used or novel genotype generated. Describe any experiments used to assess the effect of a mutation and, where applicable, how potential secondary effects (e.g. second site T-DNA insertions, mosiacism, off-target gene editing) were examined.
