# Supplementary material for: A spatial and temporal transcriptomic atlas of mouse intestinal regeneration in the jejunum
Source: Nat Commun. 2026 Sep 3;17:10484. doi: 10.1038/s41467-026-77292-8 (PMC13635233; doi:10.1038/s41467-026-77292-8)
Supplement: Supplementary file 2 [file 41467_2026_77292_MOESM17_ESM.pdf]

nature portfolio

Peer Review File

A Spatial and Temporal Transcriptomic Atlas of Mouse Intestinal Regeneration in the Jejunum

Corresponding Author: Dr Michael Verzi

Parts of this Peer Review File have been redacted as indicated to remove third-party material.

This file contains all reviewer reports in order by version, followed by all author rebuttals in order by version.

Version 0:

Reviewer comments:

Reviewer #1

(Remarks to the Author)
In this manuscript, Ofer and colleagues leverage the VisiumHD platform to profile spatial gene expression changes across multiple days post irradiation in a mouse intestinal damage model. The authors irradiate mice and collect jejunal samples from multiple days, spanning from a Day 0 no damage control to Day 6. They combine these samples such that they characterize 23 biological replicates across 4 Visium HD chips.

The authors then mine these data to draw a few high-level conclusions. First, they demonstrate that clustering on the 8-micron-aggregated bins of their VisiumHD data produce broad clusters that largely agree with both expected gut anatomical structures and, to some degree, individual cell types. This provides a base level validation of the VisiumHD data. They then note an interesting spatial pattern in the expression of a handful of interferon stimulated genes. They next note that as the damage-recovery process proceeds there is a rearrangement of the clusters they identify and the emergence of some day-specific clusters. In particular, they argue that the emergence of a new bottom villus cluster at day 3 is consistent with a maturing of the lower epithelial compartment. They then describe two structures that they associate with late and early regenerative epithelial features, argue that they are defined by unique gene expression signatures and that they are found in unique spatial niches that change throughout the recovery process. They provide evidence that the epithelial compartment takes on a hypertranscription phenotype in response to damage that is supported by a local increase in the production of enzymes associated with nucleobase production. Finally, they use spatially constrained receptor-ligand analyses to suggest potential regeneration-associated signaling pathways.

Overall, the data seem to be of fine quality, and I suspect that they will indeed be a useful resource for the field. However, I am generally unenthusiastic about this work as it is entirely descriptive, there are relatively few specific biological claims, and for these claims there is no independent testing or validation. As such, I think that the biggest contribution of this work will be the ability of others to download and investigate the data.

I have a series of additional concerns that I provide below in no specific order.
1) At multiple places the authors highlight the number of genes measured, 19k, and the sub-cellular resolution of VisiumHD. Yet, in many ways, this method does not provide practical sub-cellular resolution (as more than one bin are typically analyzed), and it is unclear if 19k genes were truly detected in a meaningful way. I think the manuscript does not benefit from making either point, and the authors may wish to remove them.
2) The observation of spatial patches of ISG expression is very interesting. A similar phenomenon has been noted by Timothy Nice and Megan Baldridge and linked to interferon-lambda signaling. Could the authors embed their observations in those previous studies?
3) The authors conclude that at Day 3 the bins assigned to the bottom villus display a more mature-like signature as compared to the bins assigned to the bottom villus region at day 0; however, they provide a relatively sparse discussion of what makes these clusters more mature-like. Can they expand their discussion on the genes associated with difference? They highlight a few pathways but do not make clear why these are associated with epithelial maturity. Critically, can they confirm that these genes are epithelial associated, as opposed to differences driven by different cell populations in the underlying lamina propria. This later point is particularly important as one would not expect true single-cell resolution with VisiumHD; thus, these gene expression signatures could be driven by changes in non-epithelial cells.

4) Similarly, I found the EdU pulse chase experiments unconvincing with respect to the maturation of the bottom villus region. These images would appear to show a dramatic, but transient, reduction in EdU uptake at Day 2.5/3. Yet this observation does not require that bottom epithelial cells will have a more mature-like expression profile. Moreover, if these cells look more mature at Day 3 and epithelial cell proliferation resumes post Day 3, then wouldn't one predict that this signature moves up the villus? Do the authors have evidence of such movement?
5) I found the argument for distinct expression of stem cell signatures (Olfm4/Smoc2 versus Clu/Msln) somewhat problematic. First, is there prior evidence of epithelial expression of mesothelin (Msln)? In the healthy gut, this is broadly believed to be confined to the mesothelial layer in the serosa. Similarly, can the authors provide evidence that these signatures are epithelial derived? Again, my concern arises from the non-single-cell practical resolution of VisiumHD. Perhaps a handful of RNAscope or other imaging experiments could provide a critical validation that these spatial gene expression signatures are truly derived from spatially distinct epithelial responses.
6) The argument of hypertranscription was a fascinating one to me. However, I found the evidence provided difficult to understand. The increased UMI density at Day 5 was clear and is consistent with this argument. However, I found the description associated with the additional supporting analysis provided in Figure 4d-f and the matching supplementary figures to be too sparse to follow. Specifically, I do not understand what is plotted on the x-axis of these plots, as such I do not understand how they provide evidence in support of hypertranscription.
7) Along these lines, are there clear tissues/situations/cell populations where one would expect hypotranscription—a suppression of transcription rates? Perhaps including the analysis of such conditions could provide additional support for the authors' ability to statistically define hypertranscription.
8) As a minor point, the authors highlight the upregulation of genes associated with purine and pyrimidine biosynthesis. However, would these signatures necessarily be associated with increased transcription? Wouldn't one expect similar signatures in cells that are rapidly dividing and, thus, in need of a large of supply of DNA nucleobases? Can the authors provide some clarity on this point?
9) What are the colors in Supplementary Figure 2a? This was not clear to me.

Reviewer #2

(Remarks to the Author)
The intestinal epithelium is constantly exposed to toxic environmental factors, undergoing a repeated damage-repair cycle. Although ionizing radiation (IR) is widely used as cancer therapy, it causes intestinal damage, leading serious side effects. However, the spatial and temporal mechanisms in response to IR remain unclear. Performing comprehensive spatial transcriptomic analyses (23 replicates), the authors provide new mechanistic insight into intestinal regeneration, which include analyses of spatial signaling dynamics and crypt cell behavior, as well as hypertranscription during early and late stages of regeneration. As their data will be a very useful resource for the future investigations of gut regeneration, this manuscript would likely be highly cited. The following suggestions may further improve the current manuscript.

The authors' analysis revealed the propagation of villus cells with IFN signaling during early stages of regeneration upon IR damage (Figure 1i-k). This data is novel and informative. Could the authors perform more analysis to further define which types of IFN signaling (Type I, II III) and immune cells become activated?

In Figure 2, the authors elegantly demonstrated crypt cell behavior, leading to a pause in contribution of new villus cells during early stages of regeneration? Could this be driven by cell cycle arrest in the upper crypt cells at earlier time points (day 2)?

In Figure 3, the authors' analysis showed normal and regenerative stem cell dynamics. Could the authors include D0 in their analysis (Figure 2d, e)? Did they observe the reduction of cell numbers associated with homeostatic, stem cells-associated gene expression in D2 compared to D0?

Hypertranscription is known to occur in adult stem cells and progenitors. Did the authors observe this in newly differentiated villus cells at later stages? If so, this would suggest that hypertranscription might play additional roles (more than simply fueling the increased growth demands during regeneration).

The authors showed that TGFB expression in crypt stroma is enriched during regeneration (Figure 5i). Which stromal cell types do express TGFB? Did % of cells expressing TGFB also increase?

Reviewer #3

(Remarks to the Author)
This manuscript presents a comprehensive spatial and temporal transcriptomic analysis of intestinal regeneration following irradiation-induced injury in the mouse jejunum. Overall, the study is carefully executed, technically sound, and provides a valuable community resource. The authors make effective use of high-definition spatial transcriptomics to preserve crypt–villus architecture and to resolve dynamic transcriptional changes across epithelial compartments that would be difficult to capture with earlier Visium-based approaches. The inclusion of multiple biological replicates across time points, transparent presentation of replicate-level data, and experimental validation strengthen the robustness of the conclusions.

While many individual observations, such as crypt–villus zonation or interferon-responsive programs, have been reported previously using alternative methods, this work represents, to my knowledge, the first systematic description of intestinal

regeneration using HD spatial transcriptomic technologies. The logical progression of the manuscript, moving from villus (Fig. 1), to crypt–villus junction (Fig. 2), to crypt (Fig. 3), followed by transcriptional amplification (Fig. 4) and cell–cell communication (Fig. 5), provides a coherent and well-structured narrative. The integration of single-cell RNA-seq datasets and extension of hypertranscriptional findings to human data (Fig. 4) is particularly compelling.

That said, there are conceptual, analytical, and presentation-related issues that should be addressed to improve clarity, coherence, and suitability for being published in Nat Comm.

Major Comments

1. Throughout the manuscript, the authors alternate between different clustering approaches (K-means vs. graph-based clustering) and spatial units (8-bin segmentation vs. cell segmentation) without clear justification. This is particularly confusing in Figure 1 (panels c–g) and Figure 5, where different strategies appear to be used within the same figure.
o The authors should clearly explain the rationale for each clustering method, specify which approach is used for downstream analyses, and ideally standardize the strategy across figures to improve consistency and interpretability.
2. In Figure 2b, the authors describe the emergence of a new cluster at the crypt–villus junction at day 3 post-irradiation. However, macrophage recruitment is emphasized in Figure 2a, while macrophage or monocyte contributions to the newly emerging cluster are not clearly demonstrated.
o The biological identity and relevance of this new cluster should be clarified. Additional marker analysis or functional interpretation would strengthen the claim that this cluster is regeneration-specific and biologically meaningful.
3. The comparison of differential gene expression between “bottom villus day 0” and “bottom villus day 3” is based on two different clusters (green vs. yellow). Since these regions are defined by distinct clusters, transcriptional differences are inherently expected, making it difficult to disentangle temporal effects from cluster identity effects.
o A more informative analysis would include comparisons of the same cluster across time points, or a direct comparison between the green and yellow clusters across all samples, followed by interpretation of their temporal replacement.
4. The study is restricted to the jejunum, yet the title refers broadly to “mouse intestinal regeneration.” Given known regional differences across the small intestine and colon, this title overgeneralizes the scope of the findings.
o The title and conclusions should be adjusted to reflect the jejunal focus, or additional regions should be included to support broader claims.
5. While the dataset is valuable, parts of the manuscript remain largely descriptive, particularly the discussion of $Isg15^{+}$ villi (Fig. 1i). The proposed villus–villus communication is intriguing but underdeveloped, lacking mechanistic hypotheses or exploratory analyses (e.g., differences in cell-type composition or signaling states).
o Some deeper analytical insight or hypothesis-driven exploration would strengthen the manuscript’s impact.

Minor Comments

1. The analysis code does not appear to be publicly available in the provided materials. While this may be addressed upon publication, explicit clarification is recommended.
2. Canonical jejunal markers should be shown to clearly confirm tissue identity.
3. In Supplementary Figure 1b, QC metrics are shown per chip rather than per sample. Given that multiple samples are processed on the same chip, per-sample QC metrics should be provided to exclude sample-specific biases.
4. The labels “Early” and “Late” are potentially misleading, as “Early” populations persist at later time points. Consider alternative terminology.
5. Cell segmentation is used in some panels (e.g., Fig. 5a–f) and 8-bin segmentation in others (Fig. 5g). A consistent approach would improve clarity.

Version 1:

Reviewer comments:

Reviewer #1

(Remarks to the Author)
In this resubmission, Verzi and colleagues have add extensive new analysis and updated and clarified their text. I found the revised manuscript to be strengthened. I particularly thank the authors for a careful response to all of comments.
They have addressed all of my concerns, and I am happy to support the publication of this work.

Reviewer #2

(Remarks to the Author)
The authors have addressed my suggestions comprehensively, having signficantly improved the manuscript. Therefore, I recommend this revised manuscript for publication.

Reviewer #3

(Remarks to the Author)
The authors have adequately addressed all of my concerns. I have no further issues to raise and recommend publication, provided that the level of novelty remains compelling after considering the concerns raised by Reviewers 1 and 3.

Open Access This Peer Review File is licensed under a Creative Commons Attribution 4.0 International License, which permits use, sharing, adaptation, distribution and reproduction in any medium or format, as long as you give appropriate credit to the original author(s) and the source, provide a link to the Creative Commons license, and indicate if changes were made.
In cases where reviewers are anonymous, credit should be given to 'Anonymous Referee' and the source.
The images or other third party material in this Peer Review File are included in the article's Creative Commons license, unless indicated otherwise in a credit line to the material. If material is not included in the article's Creative Commons license and your intended use is not permitted by statutory regulation or exceeds the permitted use, you will need to obtain permission directly from the copyright holder.
To view a copy of this license, visit https://creativecommons.org/licenses/by/4.0/

REVIEWER COMMENTS

We thank the reviewers for their time and generally positive response to our resource manuscript and the value they believe it will have for the field of intestinal regeneration. In response to their thoughtful and constructive feedback, we have added 13 new figure panels, including one new supplementary figure, and revised 25 existing panels, which together have substantially strengthened this work. We thank the editorial team and Dr. Mimmi Mononen for inviting us to provide a revised manuscript, and we thank the reviewers again for the helpful suggestions on how to improve this work! We address all the reviewers' feedback in a point-by-point response below.

Reviewer #1 (Remarks to the Author):

In this manuscript, Ofer and colleagues leverage the VisiumHD platform to profile spatial gene expression changes across multiple days post irradiation in a mouse intestinal damage model. The authors irradiate mice and collect jejunal samples from multiple days, spanning from a Day 0 no damage control to Day 6. They combine these samples such that they characterize 23 biological replicates across 4 Visium HD chips.

The authors then mine these data to draw a few high-level conclusions. First, they demonstrate that clustering on the 8-micron-aggregated bins of their VisiumHD data produce broad clusters that largely agree with both expected gut anatomical structures and, to some degree, individual cell types. This provides a base level validation of the VisiumHD data. They then note an interesting spatial pattern in the expression of a handful of interferon stimulated genes. They next note that as the damage-recovery process proceeds there is a rearrangement of the clusters they identify and the emergence of some day-specific clusters. In particular, they argue that the emergence of a new bottom villus cluster at day 3 is consistent with a maturing of the lower epithelial compartment. They then describe two structures that they associate with late and early regenerative epithelial features, argue that they are defined by unique gene expression signatures and that they are found in unique spatial niches that change throughout the recovery process. They provide evidence that the epithelial compartment takes on a hypertranscription phenotype in response to damage that is supported by a local increase in the production of enzymes associated with nucleobase production. Finally, they use spatially constrained receptor-ligand analyses to suggest potential regeneration-associated signaling pathways.

Overall, the data seem to be of fine quality, and I suspect that they will indeed be a useful resource for the field. However, I am generally unenthusiastic about this work as it is entirely descriptive, there are relatively few specific biological claims, and for these claims there is no independent testing or validation. As such, I think that the biggest contribution of this work will be the ability of others to download and investigate the data.

We thank the reviewer for acknowledging the quality of our data and the value of this work as a resource for the field. It is for this reason that we made the data immediately available upon manuscript submission. Given that we have received many requests for use of the data from other labs and that the preprint has been downloaded ~1,500 times on bioRxiv, we are hopeful that this resource will be valuable to the research community. We feel that publication in an

open-access journal such as *Nature Communications* will further amplify its impact. We also appreciate the reviewer's acknowledgment that some findings could be bolstered with additional validation, and we have worked to improve the manuscript accordingly. We thank the reviewer for their valuable feedback.

I have a series of additional concerns that I provide below in no specific order.
1) At multiple places the authors highlight the number of genes measured, 19k, and the sub-cellular resolution of VisiumHD. Yet, in many ways, this method does not provide practical sub-cellular resolution (as more than one bin are typically analyzed), and it is unclear if 19k genes were truly detected in a meaningful way. I think the manuscript does not benefit from making either point, and the authors may wish to remove them.

We agree that our original wording may have overstated the practical subcellular resolution and the ability to detect all ~19,000 genes represented by the probes used. In the revised manuscript, we have removed language implying subcellular interpretation, as our analyses are now performed mostly using 2um bins regrouped into segmented cells. To more accurately represent the genes detected in our dataset, we applied a minimum expression threshold requiring detection in at least 50 cells in the dataset, which yielded 16,921 detected genes. These results are also consistent with what has been observed in the human protein atlas study, which found that several thousand genes are not expressed in any given tissue (PMC3916642). We hope that this more precise representation of the data resolution will assist readers and future investigators who use our data, and we thank the reviewer for this opportunity to more accurately describe our data.

2) The observation of spatial patches of ISG expression is very interesting. A similar phenomenon has been noted by Timothy Nice and Megan Baldridge and linked to interferon-lambda signaling. Could the authors embed their observations in those previous studies?

We thank the reviewer for highlighting this important body of work by Nice and Baldridge and for the suggestion to embed our observations within these prior studies. Situating our findings within the Nice and Baldridge framework substantially corroborates their observations and presents new opportunities to use our data to better understand the spatial patterning of IFN target gene expression. We have revised the manuscript accordingly and have added Supplemental Figure 2 (and Supplemental Table 3) in response to these suggestions.

Van Winkle, Nice et al. (*eLife* 2022) demonstrated that IFN-λ drives focal, epithelial-restricted ISG expression in discrete single villi at homeostasis, a response dependent on commensal bacterial sensing and epithelial IFNLR1 expression. Building on this, Constant et al. (*Gut Microbes*, 2025) characterized the transcriptional identity of these homeostatic *Ifit1*$^{+}$ pockets by combining RNAscope-guided laser capture microdissection with bulk RNA sequencing of serial villus sections, generating a transcriptional signature of the IFN-λ–primed epithelial state.

[FIGURE REDACTED]

Figure R1: top panels Van Winkle et al., *elife* 2022 (PMID: 35137688); bottom panel Constant et al., *Gut microbes* 2025 (PMID: 41411655). These data show results consistent with what we observe in our spatial datasets; at homeostasis, occasional villi are positive for ISG expression.

The patchy *Isg15*⁺ villus expression we observe in the spatial transcriptomics data at day 0 (non-irradiated control; Figure R2 below and in main text Figure 1i) is morphologically and transcriptionally consistent with this paradigm, sharing a similar downstream IFN-λ effector program, albeit arising in a distinct biological context. We have embedded our findings within this framework explicitly in the revised results and discussion (main Figure 1i and Supplemental Figure 2), including citations of this prior work.

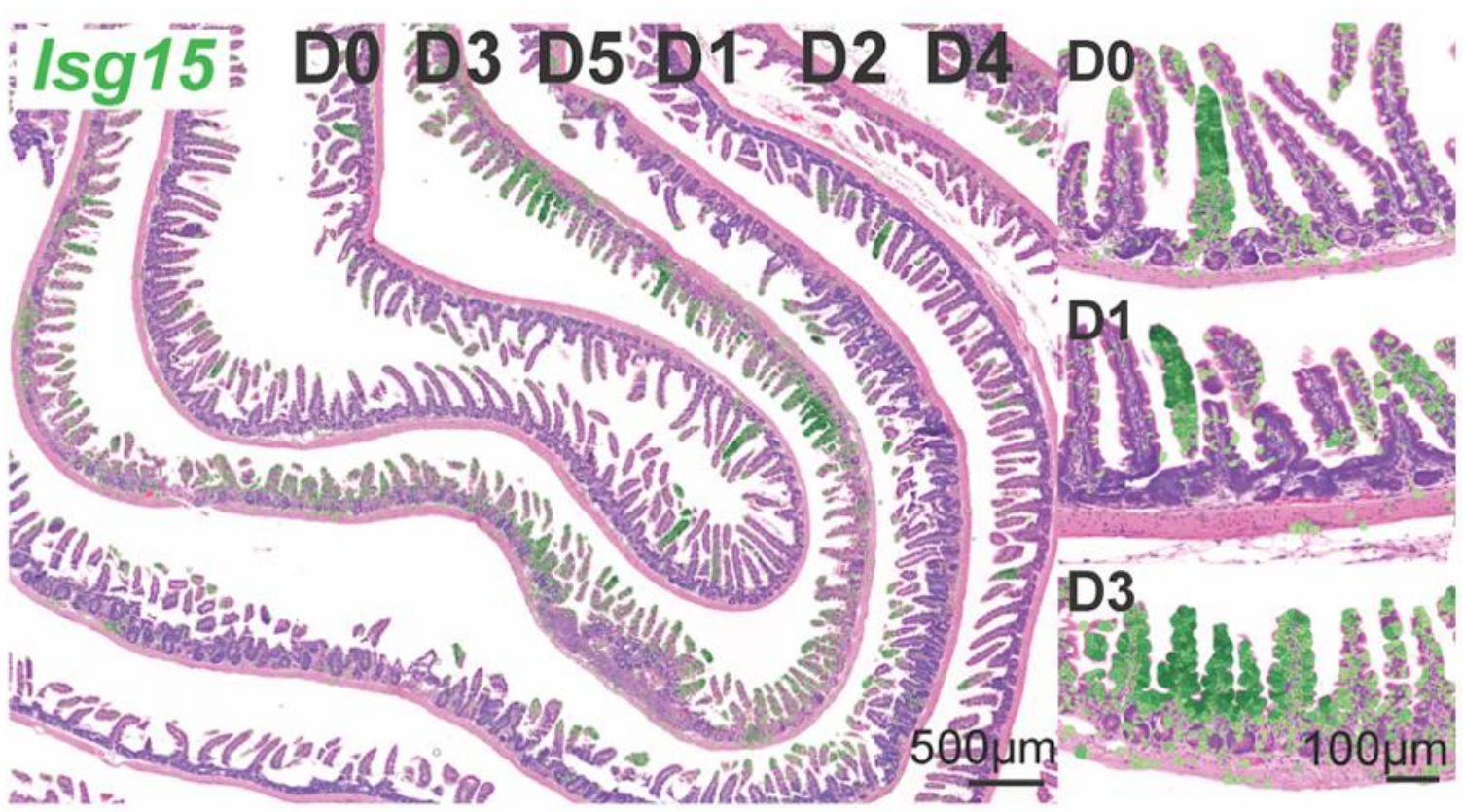

Figure R2: (main Figure 1i) Spatial transcriptomic mapping of interferon signaling genes reveals region-specific expression patterns during homeostasis and regeneration. Log2-transformed expression of *Isg15*, with cell segmentation highlighting *Isg15*$^{+}$ villi of day 0,1 and 3 post-IR.

To specifically determine whether our spatially restricted ISG expression recapitulates the IFN-λ–driven epithelial program described by Nice, Baldridge and colleagues, we mapped their published *Ifit1*$^{+}$ gene signature onto our spatial transcriptomics data. Constant et al. (2025) defined the *Ifit1*$^{+}$ gene set using the laser capture microdissection of *Ifit1*-expressing villi identified by RNAscope (top 30 genes). The spatial expression domain of these *Ifit1*-associated genes closely matched that of our previously defined *Isg15*$^{+}$ population (Figure R3), indicating that the *Ifit1*$^{+}$ signature marks localized epithelial regions with coordinated ISG upregulation.

[FIGURE REDACTED]

Figure R3: *Isg15*$^+$ bins in spatial data (left); top 30 genes in the *Ifit1*$^+$ gene signature from Constant, et al., *Gut microbes* 2025 (right) recapitulate the spatial distribution of the IFN-high cells we defined as *Isg15*$^+$.

To more comprehensively compare the *Ifit1*$^+$ gene signature described by Constant et al. (*Gut microbes*, 2025) to our interferon-active bins, we identified differentially expressed genes between *Ifit1*$^+$ vs. *Ifit1*$^-$ day 0 homeostatic villus epithelial bins, yielding 188 genes (adj.*P* <0.05, mean normalized average (MNA)>0.05, L2fc>2). Gene ontology analysis of genes enriched in *Ifit1*$^+$ bins revealed significant enrichment of antiviral and interferon-related pathways, consistent with GO terms reported for the published *Ifit1*$^+$ signature (Constant et al., *Gut microbes* 2025; left panel below).

[FIGURE REDACTED]

Figure R4: Spatial transcriptomic profiling of *Ifit1* expression across day 0 and day 3 villus epithelium identifies discrete *Ifit1*$^+$ and *Ifit1*$^-$ epithelial bins. GO term enrichment analysis of genes upregulated in *Ifit1*$^+$ versus *Ifit1*$^-$ day 0 villus bins (188 genes; *P* adj.<0.05, MNA>0.05, L2fc > 2) reveals enrichment of innate immune response, interferon

signaling, and antimicrobial defense pathways, consistent with HALLMARK pathway signatures reported for *Ifit1*$^{+}$ intestinal epithelial cells (Constant et al., *Gut Microbes*, 2025) (left panel). GSEA comparing *Ifit1*$^{+}$ vs. *Ifit1*$^{-}$ day 0 epithelial cells confirm strong enrichment of the published *Ifit1*$^{+}$ cell gene signature (201 genes, Constant et al., *Gut microbes* 2025) in spatially defined *Ifit1*$^{+}$ bins (NES = 2.47, FDR = 0.0), demonstrating that spatial binning faithfully recapitulates the transcriptional identity of *Ifit1*$^{+}$ epithelial cells (right panel).

Gene set enrichment analysis (GSEA) further confirmed strong concordance between our spatial *Ifit1*$^{+}$ bins and the *Ifit1*$^{+}$ gene signature of Constant et al. (*Gut microbes*, 2025), demonstrating that the focal ISG expression we observe in our data reflects a highly similar interferon-driven epithelial program to that described by Nice and colleagues (Figure R4;right panel).

For further analyses extending our interferon target gene findings into the irradiation response and regeneration, please see our responses to the other reviewers and Supplemental Figure. 2.

3) The authors conclude that at Day 3 the bins assigned to the bottom villus display a more mature-like signature as compared to the bins assigned to the bottom villus region at day 0; however, they provide a relatively sparse discussion of what makes these clusters more mature-like. Can they expand their discussion on the genes associated with difference? They highlight a few pathways but do not make clear why these are associated with epithelial maturity. Critically, can they confirm that these genes are epithelial associated, as opposed to differences driven by different cell populations in the underlying lamina propria. This later point is particularly important as one would not expect true single-cell resolution with VisiumHD; thus, these gene expression signatures could be driven by changes in non-epithelial cells.

We agree that this was a confusing point. The revised manuscript now provides a clearer mechanistic explanation of the "mature-like" signature, anchoring our definition of villus maturity on gene sets from an established paper that empirically defines villus maturity along the crypt-villus axis using laser-capture microdissection (Moor, et al., *Cell* 2018). We also address whether the observed gene expression differences could be driven by non-epithelial cells in the underlying lamina propria. We hope these updates will allay the reviewer's concerns and thank the reviewer for this opportunity to clarify.

Origin of the "mature-like" interpretation: Our characterization of day 3 lower villus epithelium as "mature-like" is grounded in unsupervised clustering of epithelial bins in the 8μm binned data. At day 0, epithelial bins resolved into 5 clusters (Figure R5): crypt (purple), crypt-villus junction (blue), bottom villus (green), upper villus (orange) and villus tip (red); (left panel). At day 3, the lower villus cluster (green) was entirely absent, and villus epithelial bins instead collapsed into a single cluster (orange) whose transcriptional identity corresponds to the upper villus cluster observed at homeostasis. The observed green cluster loss was reproducible across biological replicates (Figure R5; Supplemental Figure 3a). The "mature-like" designation therefore reflects two discrete observations: (1) loss of the transcriptional program characteristic of the least mature villus bottom epithelial zone under homeostasis, and (2) a shift in the remaining villus epithelium toward the upper villus gene expression program, which is associated with more differentiated epithelial cell states.

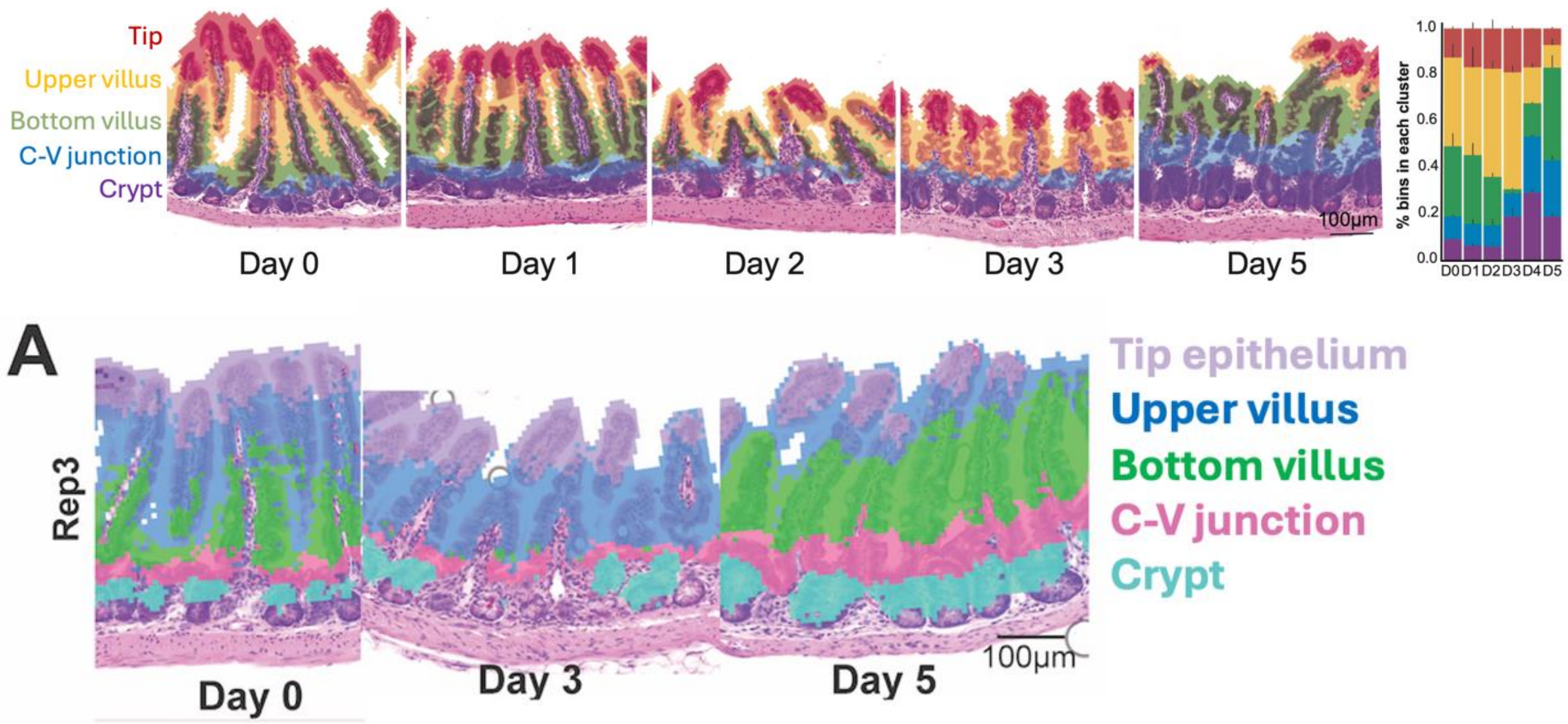

Figure R5: 8um bin data showing five epithelial clusters on days 0,1,2 and 5; On day 3 there are only 4 clusters, with loss of the green cluster which reappears on day 5 along the expansion of the blue cluster.
Quantification across all replicates is shown in the graph (right) with percent cluster per timepoint.
Bottom figure: a similar phenomenon was observed across replicates. Shown here are rep3 epithelial clusters. The green bottom villus cluster on day 0 disappears on day 3.

Genes and pathways underlying the mature-like signature: To build upon these findings as suggested by the reviewer, we now directly test whether lower villus epithelium at day 3 is transcriptionally more mature relative to day 0 lower villus epithelium. We applied the spatially resolved villus zonation gene signatures from Moor et al. (*Cell*, 2018), a canonical reference dataset generated by laser capture microdissection across five villus zones, in gene set enrichment analysis (GSEA). Comparing day 3 versus day 0 lower villus bins, we find that gene sets associated with the villus tip (zone 5, Moor et al.) are enriched in day 3 lower villus epithelium, while gene sets associated with the lower villus (zone 2, Moor et al.) are more enriched in day 0. The leading-edge genes driving zone 5 (villus tip) enrichment at day 3 include *Slc34a2*, *Aqp7*, *Gpa33*, laminin subunits *Lamb3* and *Lama3*, and *Dsp*, markers of fully differentiated villus enterocytes with established roles in nutrient transport, epithelial barrier integrity, and cell-matrix adhesion. These results provide direct, quantitative support for the mature-like interpretation and root it in an established, epithelium-specific reference framework. GSEA analysis is included in Figure 2d in the revised manuscript.

[FIGURE REDACTED]

Figure R7: villus zones 1 through 5 defined by Moor et al., *Cell*, 2018

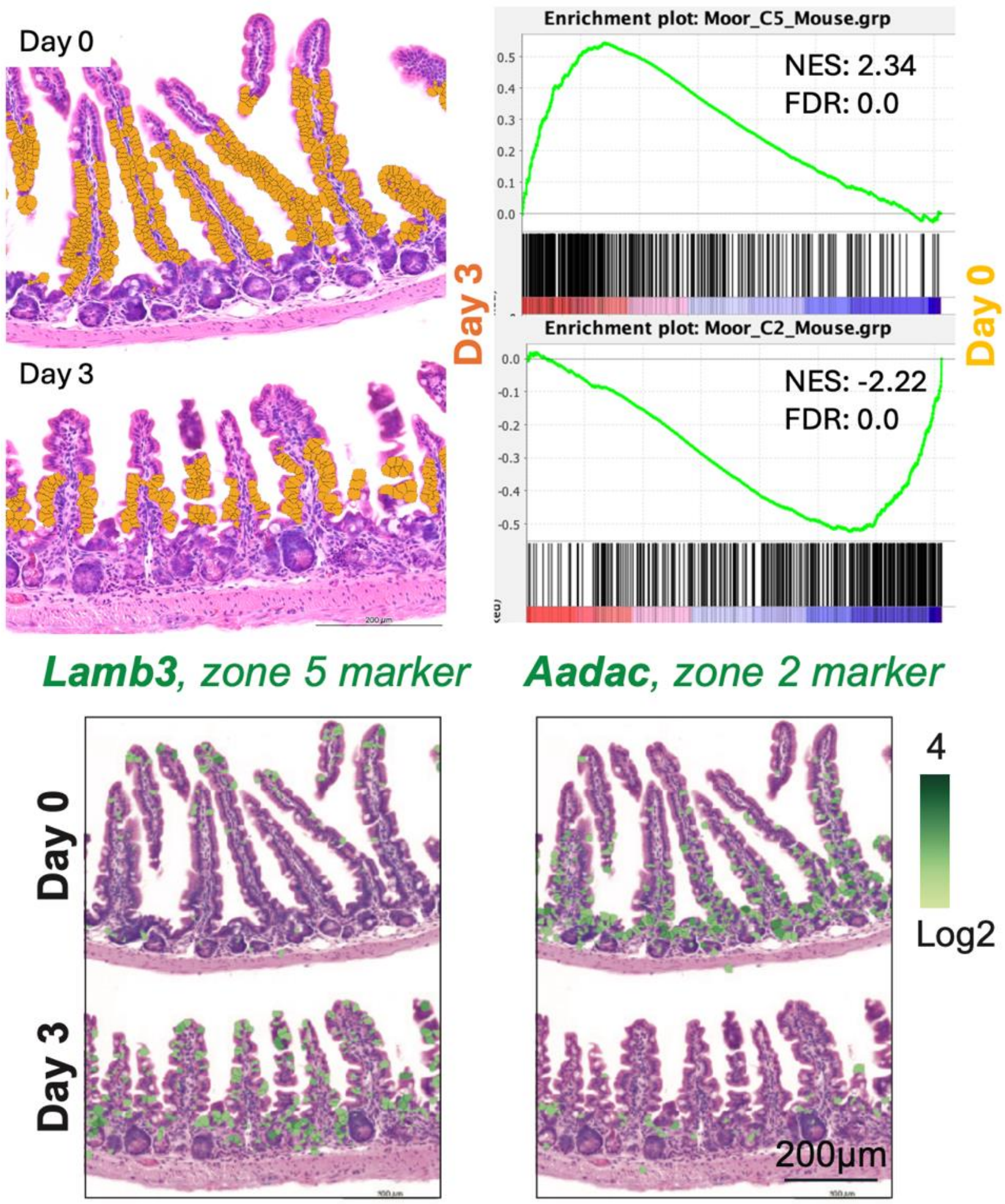

Figure R8: (top figures) shared villus epithelium cluster (k-means) shown in day 0 (top) and day 3 (bottom). GSEA analysis shows enrichment of Moor et al., 2018 zone 5 (tip epithelium) in the day 3 villus epithelium cluster, and enrichment of zone 2 (bottom villus) in day 0 villus epithelium. Zonated gene sets were derived from Moor et al., 2018. The gene expression continuum (red to blue heatmap) reflects differential expression Day 3 vs Day 0 bottom villus epithelial cluster and includes genes with MNA >0.03.
Bottom Figure: example of zonated genes expression in the spatial data. *Lamb3*, a zone 5 marker, is enriched in the day 3 post-IR timepoint. Conversely, *Aadac*, a zone 2 marker, is downregulated at the day 3 post-IR timepoint.

Finally, villus zonation gene set scoring along the crypt-to-tip axis further demonstrates the maturation shift (Figure R6; New panel, Supplemental Figure 3b). In homeostatic tissue (day 0), the lower-villus zone 2 signature peaks in the junction region (~100µm from the smooth muscle layer), but is reduced by roughly half at day 3 post-IR. Conversely, upper-villus signatures (zones 4 and 5) show increased expression at day 3, beginning at the crypt-villus junction. Together, these results indicate that the villus base epithelium acquires a more mature transcriptional identity at day 3 post-IR.

[FIGURE REDACTED]

Figure R6: Gene list expression score of villus epithelial zonation genes (Moor at al., 2018) along the crypt-villus axis on day 0, 3, and 5 post-IR. Shown as average of 10 µm windows along the crypt-villus axis as measured by distance of the cell from the smooth muscle cluster, with SEM shaded.

Evidence that these signatures are epithelial derived: We appreciate the reviewer's concern that gene expression differences between day 0 and day 3 villus bins could reflect changes in non-epithelial cells of the underlying lamina propria rather than the epithelium itself. To address this concern, we implemented a gene filtering strategy that restricts differential expression analyses to genes with confirmed epithelial expression. Differentially expressed genes were filtered against a homeostatic intestinal epithelial gene list defined by expression in bulk epithelial RNA seq at FPKM > 1 (~10,500 genes). To account for the possibility that injury induces expression of genes absent from homeostatic epithelium, we supplemented this list with genes from published intestinal injury and regeneration datasets of isolated epithelial cell populations, including irradiation-induced epithelial signatures, revival stem cell programs, and fetal reversion gene sets (Ayyaz et al., *Nature* 2019, Yui et al., *Cell Stem Cell* 2018, Chen et al., *Cell Stem Cell* 2023, Mustata et al., *Cell* 2013, Munoz et al., *EMBO J* 2012, Moor et al., *Cell* 2018, Wang et al., *Cell* 2019; Supplementary Table 4). We have also updated Figure 1g GO term analysis using the cell segmented data. Together, these filters ensure that reported transcriptional differences reflect genes with confirmed epithelial expression, minimizing the contribution of lamina propria-derived signal to our findings.

That said, we acknowledge the important caveat raised by the reviewer; VisiumHD does not achieve true single-cell resolution, and mixed epithelial-stromal signal within individual bins cannot be fully excluded. The revised manuscript prioritizes 2µm bin data segmented using the 10x Genomics machine-learning approach, and substantially reduces this concern, as cell-segmented 2 µm bins better approximate the dimensions of individual intestinal epithelial cells than the previous 8 µm bin approach. This substantially reduces- though does not eliminate- the

likelihood that lamina propria-derived signal drives the transcriptional differences we report. Combined with our epithelial gene filtering strategy and the use of the Moor et al. epithelium-specific leading-edge genes to anchor out interpretations, we are confident that the signatures described largely reflect genuine epithelial transcriptional changes. We have added explicit acknowledgment of this limitation to the revised discussion.

4) Similarly, I found the EdU pulse chase experiments unconvincing with respect to the maturation of the bottom villus region. These images would appear to show a dramatic, but transient, reduction in EdU uptake at Day 2.5/3. Yet this observation does not require that bottom epithelial cells will have a more mature-like expression profile. Moreover, if these cells look more mature at Day 3 anad epithelial cell proliferation resumes post Day 3, then wouldn't one predict that this signature moves up the villus? Do the authors have evidence of such movement?

We agree that the EdU pulse-chase experiments alone do not demonstrate epithelial maturation, but rather indicate a transient reduction in proliferation at early time points (days 2.5-3.5), consistent with a temporary pause in crypt-derived cell contribution to the villus. We have noted this caveat in the revised text, as shown below. We expand on these conclusions further:

Transcriptional Evidence for a Mature-like Epithelial State at Day 3: Our interpretation of a more differentiated ("mature-like") epithelial state at day 3 is based on a combination of observations, of which transcriptional analysis of day 3 epithelial bins is the primary evidence (as demonstrated in the response to the previous comment) rather than EdU labeling alone. The Figure below demonstrates that while the crypt-villus junction cluster in green represents a similar proportion of the epithelium on days 0 and 3 (~13% of villus epithelium), the villus cluster (in orange) is relatively smaller while the tip cluster expands on day 3. This is consistent with villus enterocytes continuing to mature toward a tip-like transcriptional state during a period when few new crypt-derived cells are being contributed to the villus. We also observe the tip cluster being proportionally larger at day 3.

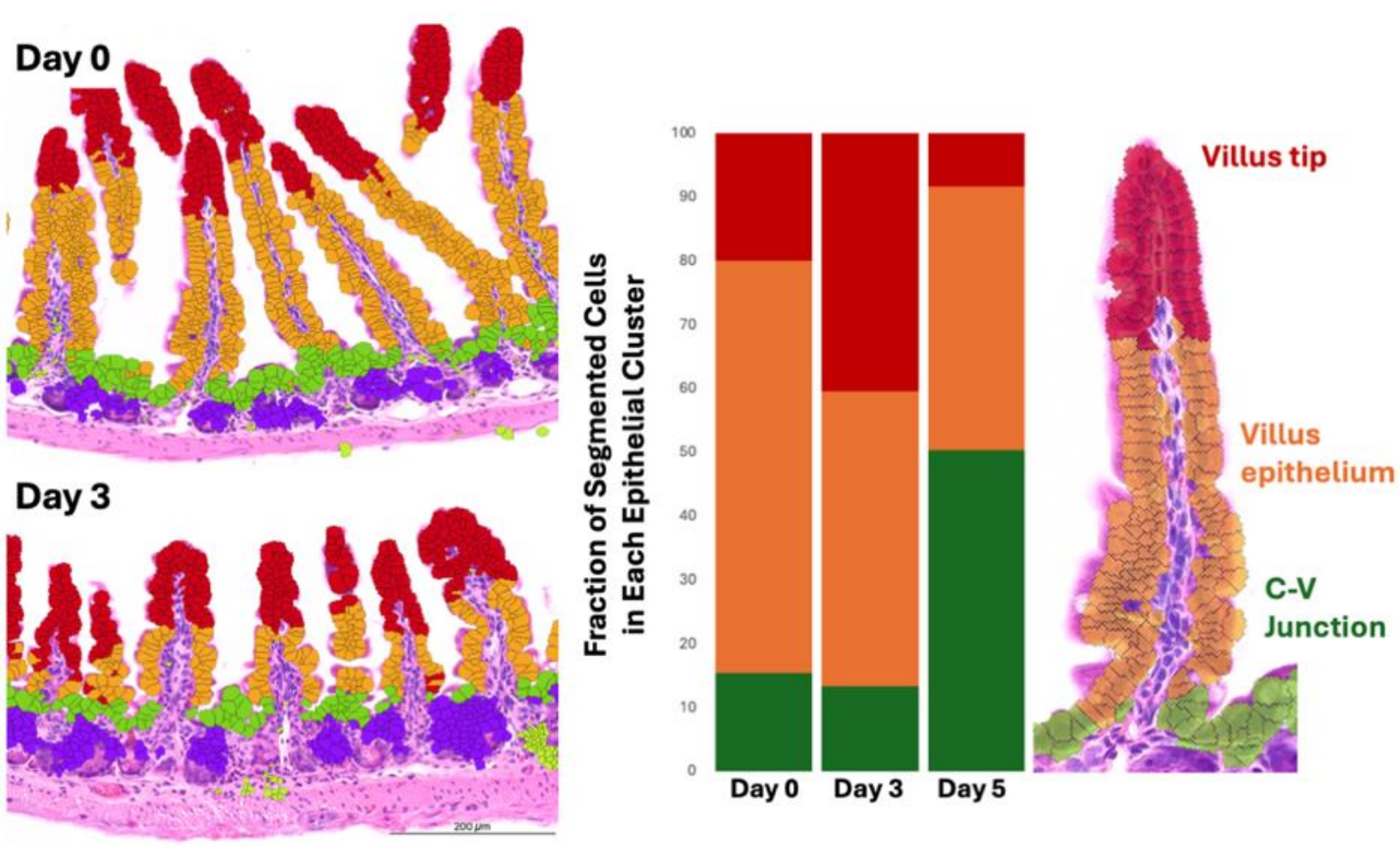

Figure R9: k means cell segmentation clusters (left); Percent villus epithelium cluster for junction, villus and tip epithelium on days 0,3 and 5 (right)

Consistent with this model of continuing maturation of villi at day 3 post-IR, previous work by our lab (Chen et al., *Cell stem cell*, 2023) showed a dramatic reduction in 24h BrdU pulse-chase labeling on the villi between days 1 and 3 post IR, which resumed at an increased rate by day 5, as predicted by the reviewer! (See Figure R10, panel A) These findings are consistent with our current study's attempt to recapitulate these findings with a pulse-chase of EdU on day 3.5 post IR for 24 hours (EdU, S phase green). While active proliferation was maintained in the crypt (KI67, pink), newly labeled cells did not contribute to the villus until day 4.5 (see Figure R10, panel I, below). This observation is consistent with previously published data from our lab and supports the idea that existing villus cells could remain at the villus base and continue to mature under these timepoints of regeneration.

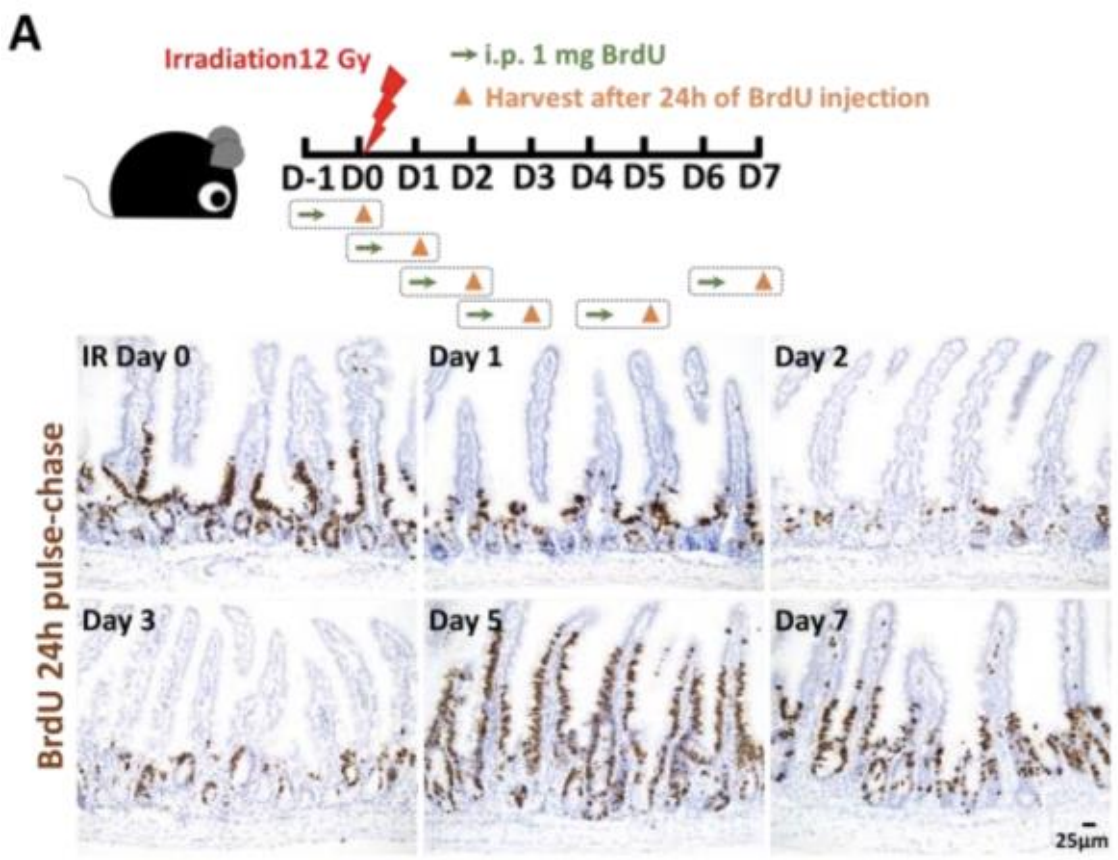

[FIGURE REDACTED]

Figure R10: Left: main Figure 2i: EdU staining reveals retention of proliferative epithelial cells in lower villus and crypt regions between days 2.5-3.5 post-IR (upper right box), followed by resumed upward migration by day 4.5 post-IR (bottom right box). This pattern suggests a delay in contribution of new cells to the villus base during regeneration. n=2.
Right Figure: 24h BrdU pulse-chase immunostaining shows loss of proliferative marker (BrdU) incorporation on the villus between days 2-3 post-IR. On day 5 post-IR proliferation resumes at a faster rate as demonstrated by an increase in BrdU incorporation relative to day 0 control. n=3. Chen et al., *Cell Stem Cell.* 2023

Indeed, the increase in migration on day 5 corresponds with evidence for upward shift of the spatial transcriptional signature: As the reviewer predicts the transcriptional signature observed in the lower villus at day 3 should shift upward once proliferation and migration resume at later timepoints. Consistent with this prediction, at day 5 the corresponding epithelial cluster (orange cluster; see Figure R5) is positioned higher along the villus axis, indicating an upward shift of this transcriptional program. In parallel, on day 5 we observe expansion of lower villus associated population (green cluster), consistent with the recovery of proliferation seen in EdU labeling at day 4.5 (Figure R10).

These findings support a three-phase model of epithelial response to irradiation. In the early phase (day 2.5-3.5), reduced proliferation limits crypt-to-villus cell transitions. In the intermediate phase (day 3), existing villus cells adopt a more differentiated transcriptional state while no new cells are coming onto the villus. In the recovery phase (day 4-5), proliferation resumes and epithelial populations re-establish upward migration along the villus axis. This leads to an expansion of cells in the lower villus domain at day 5. We note that while these data are consistent with upward cell movement, direct lineage tracing of cell migration was not

performed in this study, and it is important that we include these caveats in the revised manuscript, as suggested by this reviewer.

5) I found the argument for distinct expression of stem cell signatures (Olfm4/Smoc2 versus Clu/Msln) somewhat problematic. First, is there prior evidence of epithelial expression of mesothelin (Msln)? In the healthy gut, this is broadly believed to be confined to the mesothelial layer in the serosa. Similarly, can the authors provide evidence that these signatures are epithelial derived? Again, my concern arises from the non-single-cell practical resolution of VisiumHD. Perhaps a handful of RNAscope or other imaging experiments could provide a critical validation that these spatial gene expression signatures are truly derived from spatially distinct epithelial responses.

We thank the reviewer for this important critique, which raises two related concerns: the specific question of *Msln* epithelial expression and the broader question of interpreting VisiumHD data given its lack of true single-cell resolution. We address each in turn.

***Msln* as a marker of fetal-like epithelial reprogramming in injury contexts**: The reviewer is right to note that *Msln* is canonically a marker of the serosa. In fact, we can see this expression in serosal areas of our samples (Figure R11, Day 0 *Msln* expression). We are sorry we didn't anticipate this source of confusion.

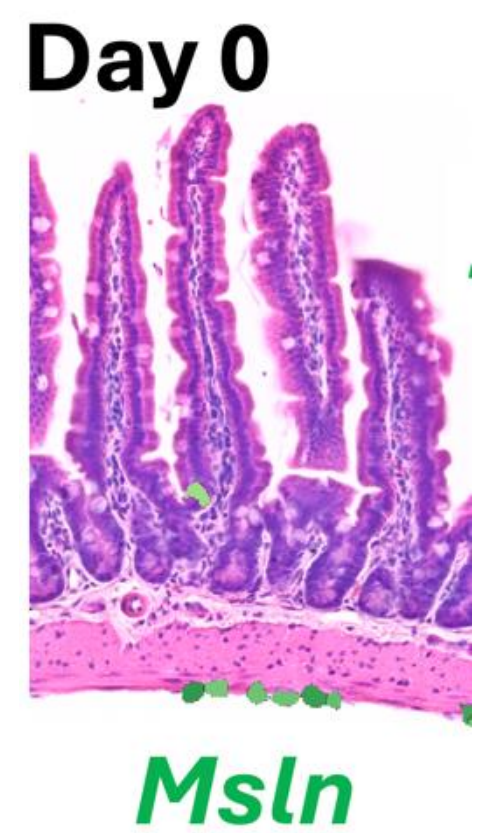

Figure R11: *Msln* expression at day 0 timepoint.

However, we can allay the concern regarding *Msln* expression in the epithelial compartment, as it has become a well-founded marker in regenerative intestinal biology. Injury-induced epithelial upregulation of *Msln* has been recently observed across multiple independent model systems as part of a fetal-like reprogramming response (Ohara, T. E., et al., *Developmental cell* 2022, Gregorieff, A., et al., *Nature* 2025, Kirino, S., et al., *Commun Biol* 2026), often co-expressed with *Clu*, *Ly6a*, and *Anxa1*- markers now broadly recognized as marking a revival stem cell/fetal reversion-like state activated during mucosal repair. The broader fetal regenerative gene signature examined in the Figure below, generated from published work provides additional context from the literature for the convenience of the reviewer. Thus, *Msln* expression in injured intestinal epithelium is not novel, but a well-documented component of the fetal reversion program and nicely recapitulated by our spatial atlas resource.

[FIGURE REDACTED]

Figure R12: Top: from Ohara et al., *Developmental cell* 2022; Bottom: from Gregorieff et al., *Nature* 2015

Spatial evidence for epithelial localization of the *Clu/Msln* signature: Several features of our data reduce the likelihood of mesothelial contamination in the epithelium. First, *Msln* expression in the stromal/epithelial regions is absent in day 0 healthy tissue and emerges specifically at day 3 post-irradiation across samples, a temporal pattern consistent with injury-induced epithelial reprogramming (Ohara, 2022; Gregorieff 2015; Kirino 2016). Second, in our cell-segmented data, *Msln*-expressing bins localize to the crypt/villus junction epithelium, anatomically distant from the serosa. Finally, triple co-localization of *Msln*$^{+}$*Clu*$^{+}$*Epcam*$^{+}$ bins confirms *Msln* expression within an epithelial context, and co-expression with *Clu* or *Ly6a* is coherent with a revival stem cell identity rather than mesothelial contamination.

[FIGURE REDACTED]

Figure R13: *Msln* expression in the spatial transcriptomics data at Day 0 and Day 3; Day 3 bins expressing co-expressing *Msln*$^+$*Clu*$^+$*Epcam*$^+$; *Msln* IHC stain from Gregorieff, *Nature*. 2015

6) The argument of hypertranscription was a fascinating one to me. However, I found the evidence provided difficult to understand. The increased UMI density at Day 5 was clear and is consistent with this argument. However, I found the description associated with the additional supporting analysis provided in Figure 4d-f and the matching supplementary Figures to be too sparse to follow. Specifically, I do not understand what is plotted on the x-axis of these plots, as such I do not understand how they provide evidence in support of hypertranscription.

We thank the reviewer for their interest in our hypertranscription analysis and for allowing us the opportunity to provide more clarity on these points, including the suggestion for additional analysis in point 7 below. The x-axis is a list of individual genes, sorted by average expression and filtered to only show the top 20% most highly expressed genes across samples for visual clarity. These plots demonstrate that regenerating cells show increased expression across their expressed transcriptome; that is, upregulation spanning both lowly and highly expressed genes, which is a hallmark of hypertranscription. To improve the clarity of these figures, we have replaced the dot plot format with lines representing the rolling mean UMI count in 100-gene windows (Figures R14, R15; new Figure. 4d-f and Supplemental Figure. 5b-e). We are glad the reviewer found this idea fascinating, and we hope the new analysis will improve clarity for the readers.

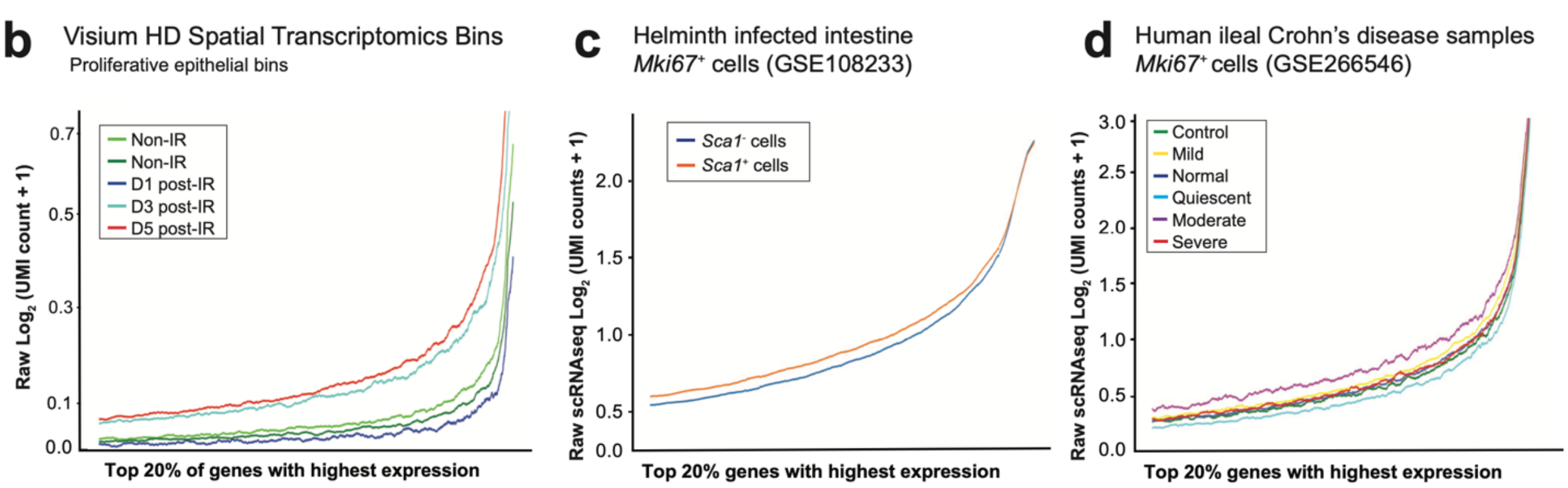

Figure R14: revised Supplemental Figure 5b-d

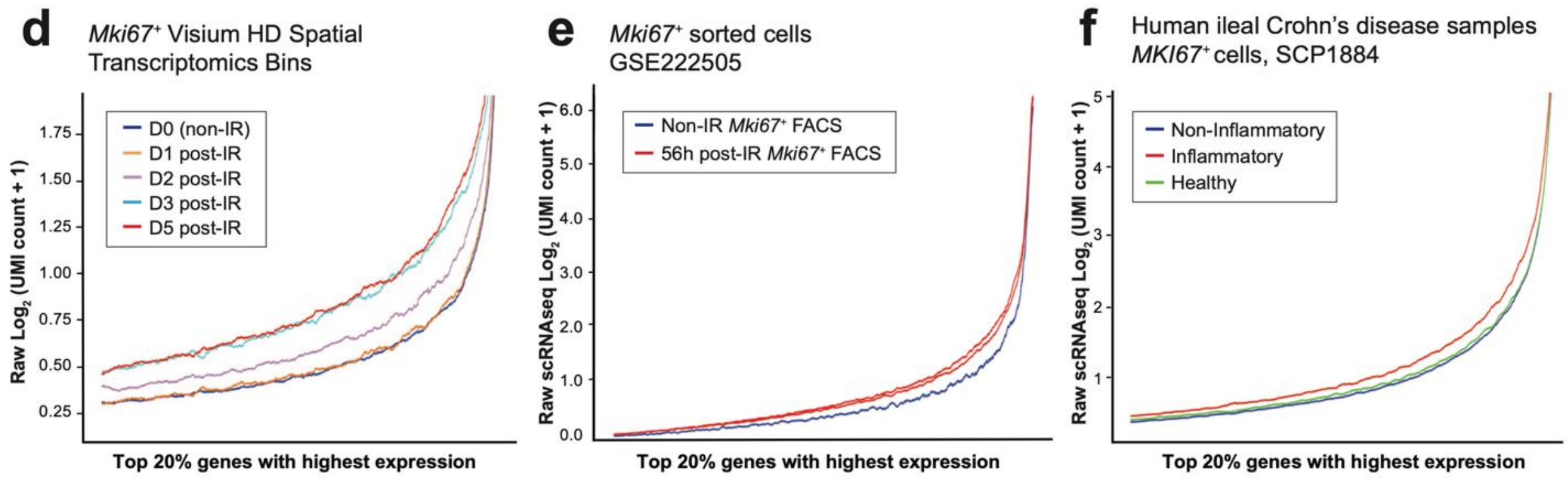

Figure R15: Revised Figure. 4d-f

7) Along these lines, are there clear tissues/situations/cell populations where one would expect hypotranscription—a suppression of transcription rates? Perhaps including the analysis of such conditions could provide additional support for the authors' ability to statistically define hypertranscription.

This is an interesting question! The reviewer's question prompted us to explore angles regarding a possible reduction in transcription across cell types and tissues within the dataset. Indeed, as the reviewer suspected, some cell populations generally show reduced UMI counts compared to others. Namely, the smooth muscle, crypt stromal populations, Peyer's patch cells, enteric neurons, and adipocytes had the lowest expression count per segmented cell, particularly compared to epithelial cell populations, as shown in the violin plot, below.

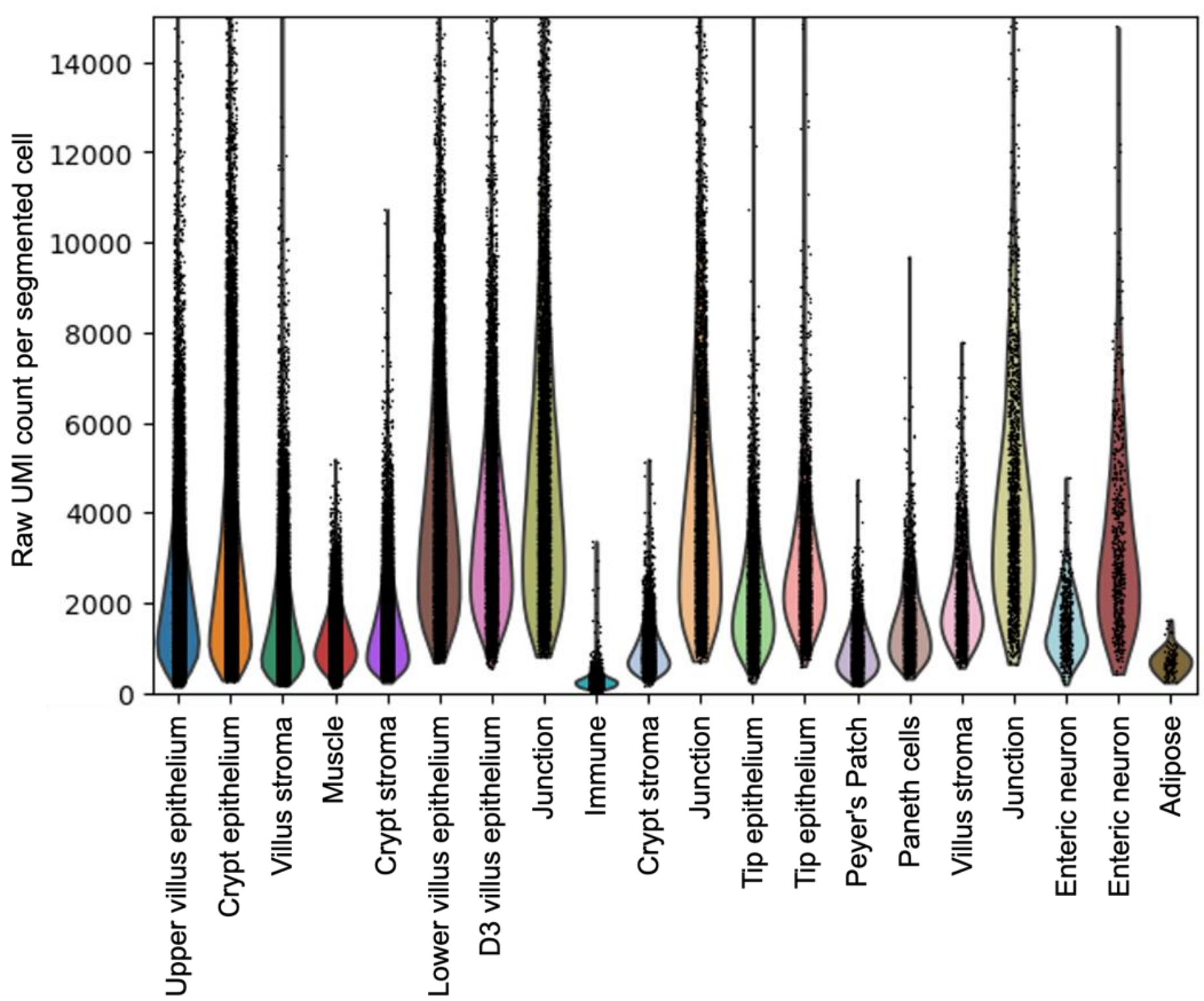

Figure R16, revised manuscript Supplemental Figure 5f: Violin plot of raw UMI counts per segmented cell across intestinal cell populations identified in the VisiumHD spatial transcriptomics dataset. Each violin plot represents the distribution of total transcript counts within a given cell cluster. Epithelial populations, including upper villus, crypt, lower villus, D3 villus, tip epithelium, and junction populations, display substantially higher UMI counts per cell relative to non-epithelial populations, including muscle, crypt stroma, villus stroma, immune cells, Peyer's patch cells, Paneth cells, enteric neurons, and adipocytes, consistent with lower transcriptional output in these non-epithelial compartments.

Next, we combined data across our 4 capture replicates (23 biological samples) for reanalysis. Raw counts were normalized per slide by computing the log2 fold-change of each timepoint's proliferative (*Ki67+)* epithelial cells relative to the proliferative epithelial cells of the day 0 non-IR control of the same slide. While a pattern of reduced transcription at day 1 was suggested in the original Figure. 4d, this new cross-replicate comparison more clearly demonstrates that proliferative epithelial cells at day 1 have reduced counts relative to their day 0 counterparts. This reduction in transcript counts corresponds with decreased expression of nucleotide biosynthesis genes at early timepoints (Figure. 4g), whose encoded enzymes increase expression during later regenerative stages. These downwards shifts in expression and transcriptional output align with a cell cycle arrest phase in the initial damage response, where the tissue loses the homeostatic crypt structure along with the stem and proliferative cell populations prior to induction of the regenerative stem cell program and subsequent hyperproliferation.

Hypertranscription thus aligns with these regenerative stem cells and their progeny. The upregulation trend across replicates at mid-to-late timepoints (day 2- 5) is further substantiated in these new plots. These data are now included in revised Supplemental Figure 5. We thank the reviewer for this suggestion and feel the manuscript is improved by these analyses.

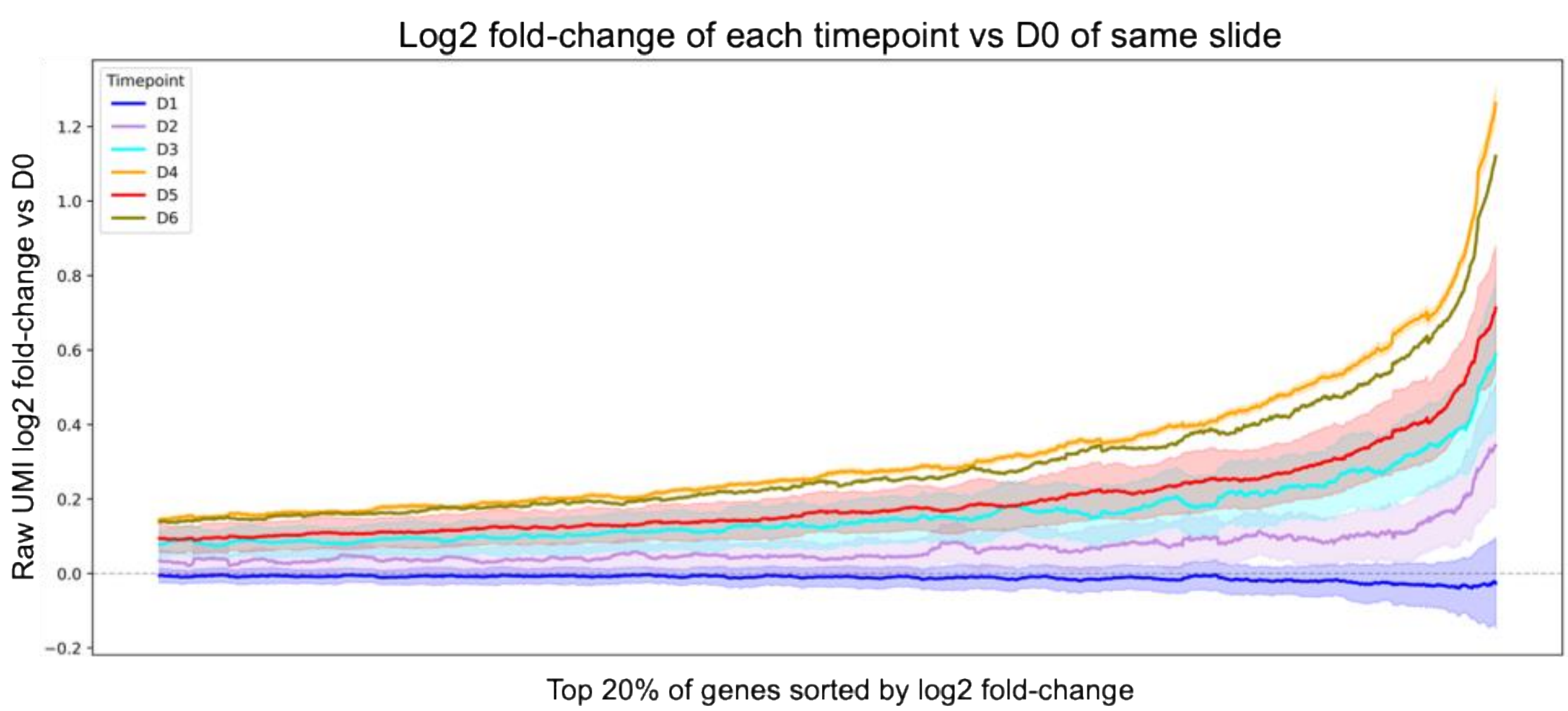

Figure R17: (revised manuscript Supplemental Figure 5e): Log2 fold-change of raw expression per bin for each timepoint relative to the day 0 tissue of the same experiment, averaged across all replicates with SEM shading. Cells were selected by co-expression of *Ki67*, *Epcam*, and *Vil1* to identify proliferative epithelial cells. The x-axis is individual genes sorted by average log2 fold-change across all replicates and timepoints.

8) As a minor point, the authors highlight the upregulation of genes associated with purine and pyrimidine biosynthesis. However, would these signatures necessarily be associated with increased transcription? Wouldn't one expect similar signatures in cells that are rapidly dividing and, thus, in need of a large of supply of DNA nucleobases? Can the authors provide some clarity on this point?

This is an important point brought up by the reviewer, and an important consideration that must be made when interpreting the data. Therefore, we have revised the text to more explicitly reflect this potential limitation and provide additional analysis to address the comment, below. In the original manuscript, we attempted to address this concern by restricting analyses to proliferating epithelial cells (*Ki67*$^{+}$) across timepoints, thereby reducing cell division-related effects in our analysis. The original Figures show that the proliferative cells in regeneration have higher mRNA levels than the proliferative cells during homeostasis. Still, as the reviewer suggests, there may be accelerated proliferation in the post-damage tissue that could explain the increase in expression of genes that promote synthesis of nucleotides.

To further explore the point raised by the reviewer about nucleotide vs deoxynucleotide synthesis, we conducted additional analyses to examine the expression of genes encoding enzymes for the rate limiting steps of purine and pyrimidine synthesis, as well as their conversion into deoxynucleotide forms (via the ribonucleotide reductase enzymes encoded by *Rrm1* and *Rrm2*). We do observe increases in expression of both total nucleotide synthesis genes and the ribonucleotide reductases that mediate deoxynucleotide conversions, likely to support the rapid cell divisions, as the reviewer suspected. Interestingly, there is potentially a temporal difference that may indicate these processes (purine and pyrimidine synthesis to support transcription of mRNA versus deoxynucleotide synthesis to support DNA replication) are distinct. While both processes are downregulated day 1 post-IR and upregulated on day 3, the deoxynucleotide-promoting *Rrm1* and *Rrm2* genes are downregulated on day 2 post-IR while nucleotide synthesis genes (*Ppat*, *Cad*, *Dhodh*, *Prps1*, *Prps2*) are upregulated (Figure R18; the first 2 colored bars represent the genes involved in deoxynucleotide conversion vs the later 4 colored bars represent nucleotide synthesis genes). This analysis suggests the possibility that mRNA synthesis - to enable the downstream hypertranscription -begins to increase prior to the cells entering their rapid division phase.

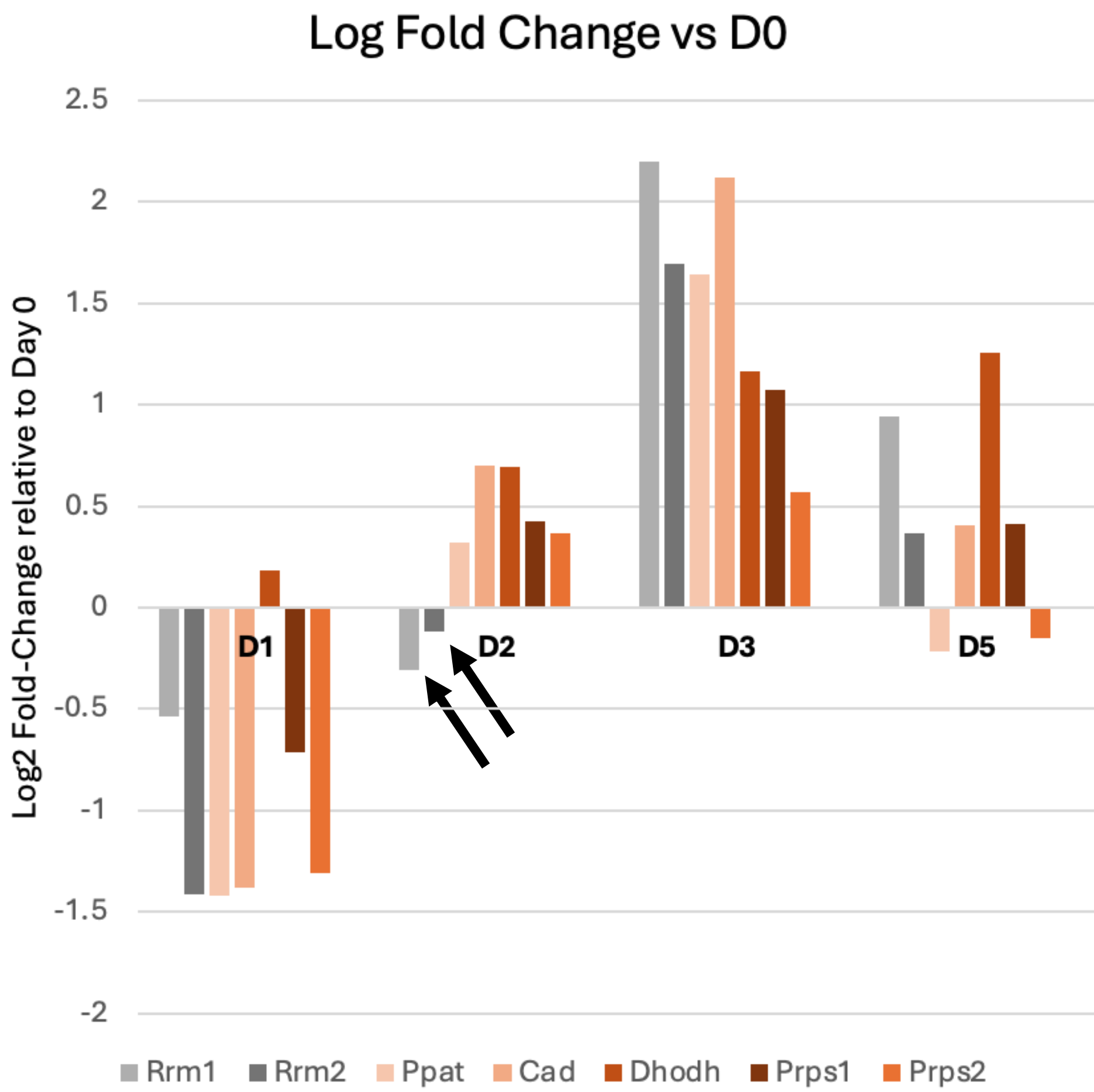

Figure R18: Log2 fold-change in expression of nucleotide synthesis genes (*Ppat*, *Cad*, *Dhodh*, *Prps1*, *Prps2*; colored bars) and deoxynucleotide synthesis genes (*Rrm1*, *Rrm2*; grey bars) in *Ki67*$^{+}$ epithelial cells across post-IR timepoints, relative to day 0. At day 2 (black arrows), nucleotide synthesis gene expression begins to increase, consistent with elevated transcriptional output, while expression of the ribonucleotide reductase genes *Rrm1* and *Rrm2*, which promote deoxynucleotide production for DNA replication, remains low until day 3.

To further decouple hypertranscription from DNA synthesis, we assessed the raw mRNA levels in non-proliferative regenerative epithelial cells (*Ki67*-negative, *Anxa1*$^{+}$) relative to proliferative homeostatic epithelial stem cells (*Ki67*$^{+,}$ *Olfm4*$^{+}$ cells at day 0). Non-proliferative epithelial regenerative cells still have a marginally elevated level of mRNA per cell, suggesting a partial decoupling of transcription increases from proliferative activity and DNA synthesis demands.

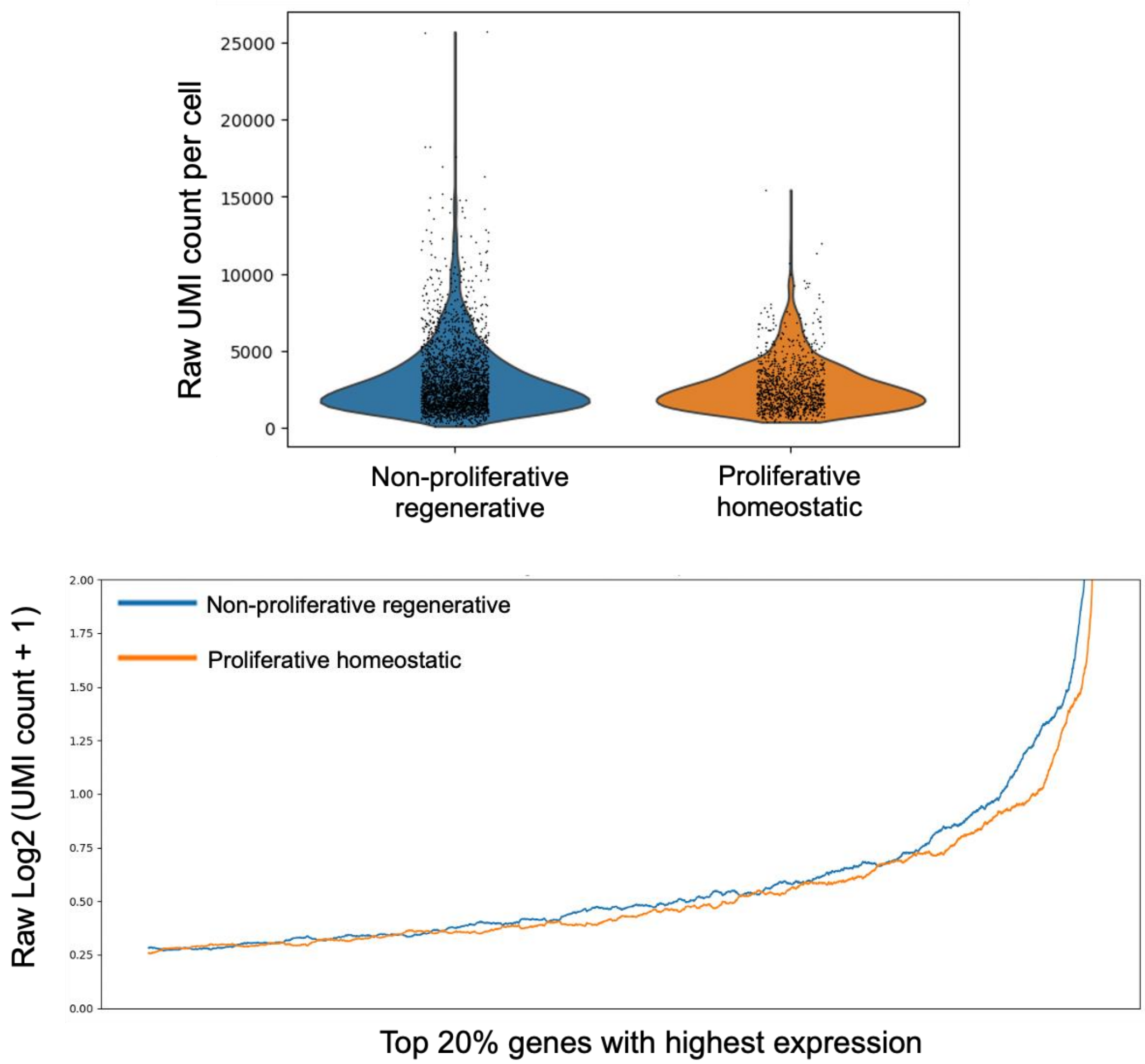

Figure R19: Raw UMI count per segmented cell (top) and raw log2(UMI count + 1) across the top 20% most highly expressed genes (bottom) comparing non-proliferative regenerative epithelial cells (Ki67^{-}, Anxa1^{+}; blue) to proliferative homeostatic epithelial stem cells (Ki67^{+}, Olfm4^{+}, day 0; orange). Even the non-proliferative regenerative cells display a consistently elevated expression across the transcriptome relative to proliferative homeostatic cells, indicating that increased mRNA content in regenerative epithelium is not simply a consequence of proliferative activity or DNA synthesis demands, and is consistent with what we might expect results from hypertranscription.

While these additional analyses support the concept of hypertranscription in intestinal regeneration, the important caveat raised by the reviewer remains, and we have added explicit discussion of this limitation to the manuscript (Results, hypertranscription section).

9) What are the colors in Supplementary Figure 2a? This was not clear to me.

The colors represent distinct clusters corresponding to epithelial zones in replicates 3 and 4; crypt epithelium, crypt-villus junction, bottom villus epithelium, mid villus epithelium and villus tip epithelium. We have made updates to the Figure to include labels of the zones. We thank the reviewer for pointing out a way to make our manuscript more clearly presented for the reader.

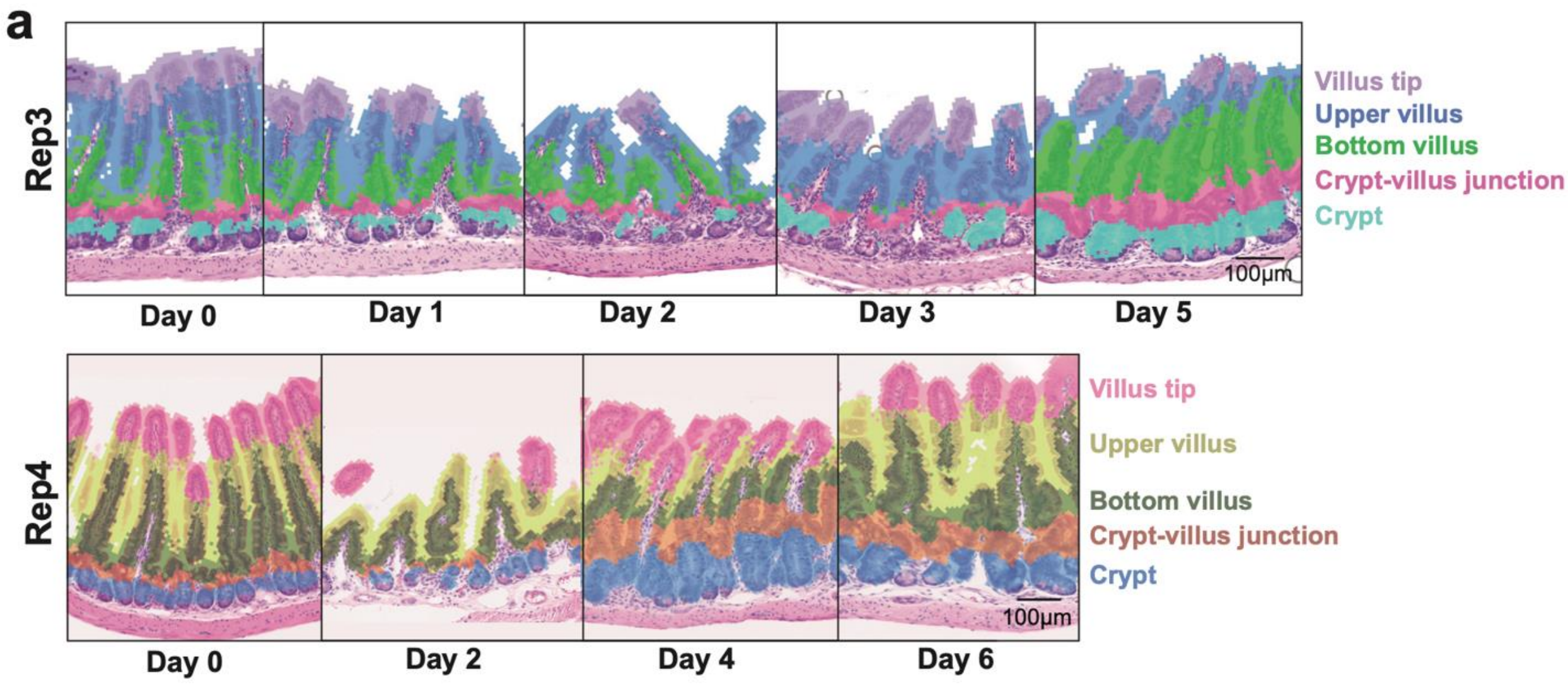

Revised Supplemental Figure 3a: epithelial regions are labeled based on position along the crypt-villus axis.

We thank Reviewer 1 for taking time to make these very helpful and interesting suggestions. We enjoyed thinking about the points the reviewer raised and conducting the new analyses that stemmed from these discussions. We hope this interchange makes a compelling point about the value of our paper as a resource for the field and that the reviewer now feels the work is suitable for publication.

Reviewer #2 (Remarks to the Author):

The intestinal epithelium is constantly exposed to toxic environmental factors, undergoing a repeated damage-repair cycle. Although ionizing radiation (IR) is widely used as cancer therapy, it causes intestinal damage, leading serious side effects. However, the spatial and temporal mechanisms in response to IR remain unclear. Performing comprehensive spatial transcriptomic analyses (23 replicates), the authors provide new mechanistic insight into intestinal regeneration, which include analyses of spatial signaling dynamics and crypt cell behavior, as well as hypertranscription during early and late stages of regeneration. As their data will be a very useful resource for the future investigations of gut regeneration, this manuscript would likely be highly cited. The following suggestions may further improve the current manuscript.

We thank the reviewer for the comments that our manuscript will be a very useful resource for the field and is likely to be highly cited, and for taking the time to make additional suggestions to further improve our work.

The authors' analysis revealed the propagation of villus cells with IFN signaling during early stages of regeneration upon IR damage (Figure 1i-k). This data is novel and informative. Could the authors perform more analysis to further define which types of IFN signaling (Type I, II III) and immune cells become activated?

We thank the reviewer for this suggestion. To further define the IFN subtypes, we performed the following additional analyses.

IFN subtype analysis. To determine which IFN subtypes are functionally engaged during intestinal regeneration, we performed GSEA and spatial analysis of IFN subtype-specific gene signatures across our Visium HD dataset (Figure R20). GSEA comparing day 3 versus day 0 villus epithelium revealed significant enrichment of Type I (HALLMARK_INTERFERON_ALPHA_RESPONSE; NES=2.47, FDR<0.0), Type II (HALLMARK_INTERFERON_GAMMA_RESPONSE; NES=2.28, FDR<0.0), and Type III IFN (derived from intestinal epithelial RNAseq of neonatal mice injected with IFN-λ3 or IFN-β;Van Winkle, *J. Virol*., 2020; NES=2.83 and 2.59, respectfully; FDR=0.0) gene signatures, suggesting possible engagement of all three IFN subtypes during regeneration.

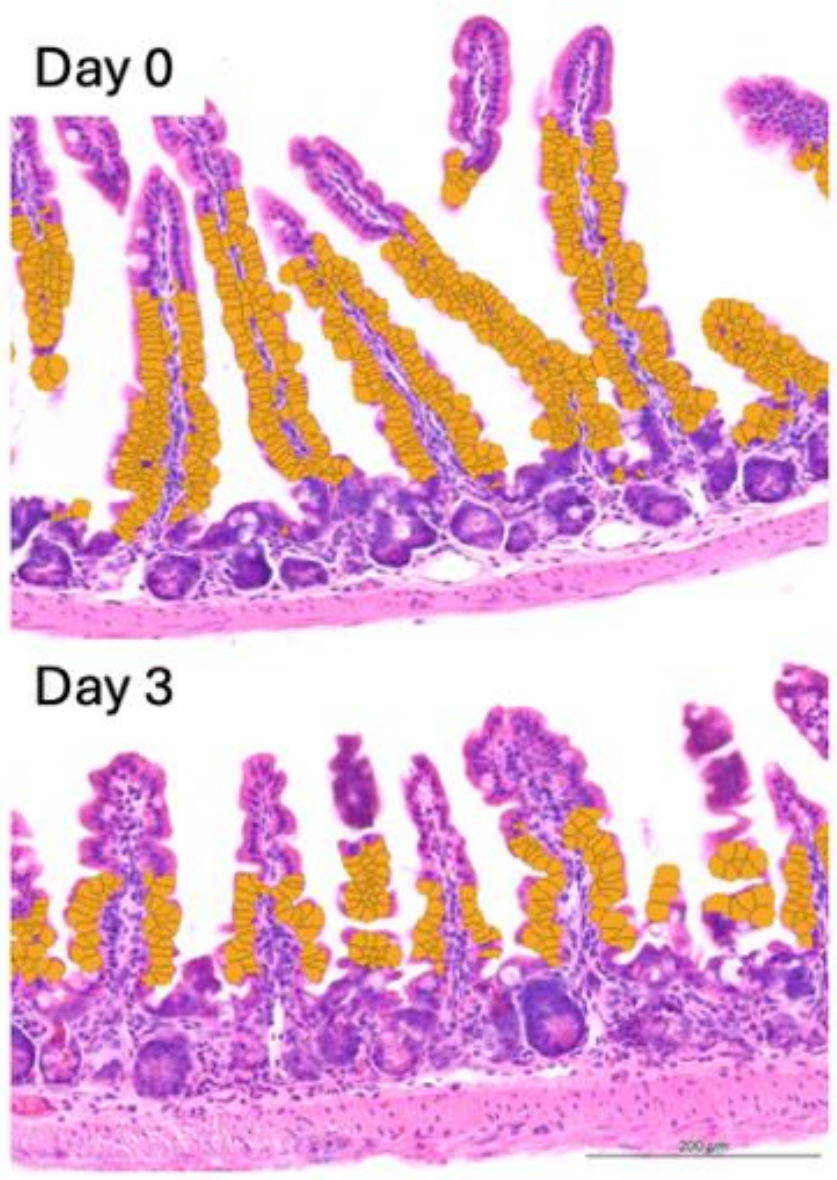

[FIGURE REDACTED]

Figure R20: (left) images of day 0 and day 3 jejunal tissues with cell segmentation overlay, showing villus epithelial clusters used for GSEA analysis. (Right) GSEA enrichment plots for Type I (HALLMARK_INTERFERON_ALPHA_RESPONSE; NES = 2.47, FDR = 0.0), Type II (HALLMARK_INTERFERON_GAMMA_RESPONSE; NES = 2.28, FDR = 0.0), Type III IFN-λ (neonateIFNL_VanWinkleMus; NES = 2.83, FDR = 0.0), and Type I IFN-β (neonateIFNB_VWMus; NES = 2.59, FDR = 0.0) gene signatures in day 3 versus day 0 villus epithelium. Gene sets for Type III IFN-λ and IFN-β signatures were derived from Van Winkle et al., *Journal of virology*, 2020.

Figure R21: Venn diagram showing overlap among the three gene signatures used in GSEA analysis: HALLMARK_INTERFERON_ALPHA_RESPONSE; HALLMARK_INTERFERON_GAMMA_RESPONSE; neonate IFNL signature (Van Winkle et al., *Journal of virology*, 2020).

[FIGURE REDACTED]

It has previously been documented that the intestinal epithelium responds to both Type I and Type III interferons, with many target genes in common (McElrath et al., *Nat Commun* 2021). Our

findings are consistent with these prior observations, and we extend them with a novel spatially resolved context.

To further characterize the observed expression patterns, we examined co-expression of all three IFN subtype signatures at days 3 and 5 (Figure R22). IFN-λ and IFN-γ induced gene signatures were broadly co-expressed across the tissue at both timepoints, extending beyond the $Isg15^{+}$/IFN-β-high regions. In contrast, the Type I IFN-β signature showed more spatially restricted expression, confined to *Isg15*-high areas, suggesting that the Type I response represents the most spatially discrete component of the IFN program during regeneration (Figure R22).

[FIGURE REDACTED]

Figure R22: spatial co-expression maps at days 0,3 and 5 post-IR showing (top) IFNL-neonate (blue) and HALLMARK_GAMMA_RESPONSE (yellow; 190 genes), and (bottom) IFNL-neonate (blue) and IFNB-neonate (yellow). Co expression is shown as a bivariate color scale (log2 average). The IFN-β signature is restricted to Isg15-high regions, while IFN-γ and IFN-λ signatures show broader tissue-wide expression at days 3 and 5. IFN-λ (210 genes) and IFN-β neonate (64 genes) gene signatures were derived from Van Winkle et al., *J. Virol.*, 2020.

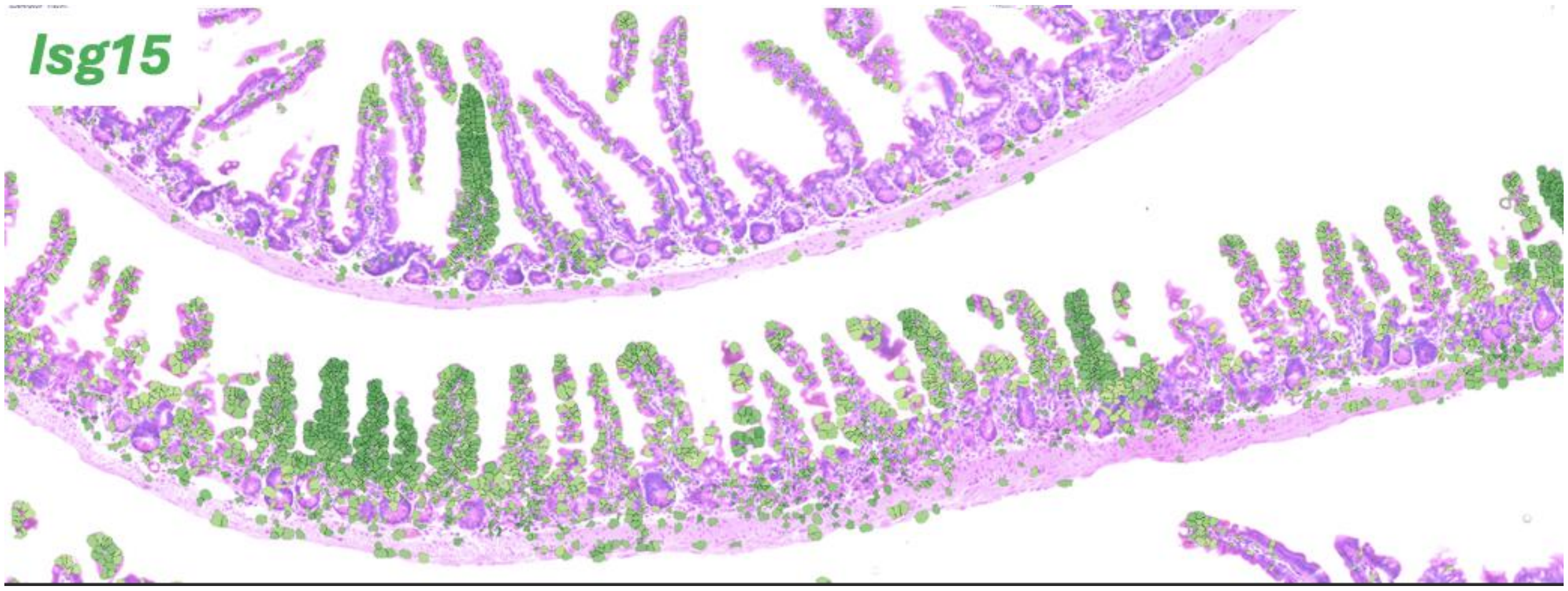

Figure R23: Log$_2$-transformed *Isg15* expression in spatial transcriptomics data at day 0 (top) and day 3 (bottom) post-irradiation, with cell segmentation overlay.

We further explore the interferon signaling pathway in response to other reviewers' questions and thank all the reviewers for their interest in this finding and chance to expand on it in the additional supplemental data panels.

In Figure 2, the authors elegantly demonstrated crypt cell behavior, leading to a pause in contribution of new villus cells during early stages of regeneration? Could this be driven by cell cycle arrest in the upper crypt cells at earlier time points (day 2)?

The reviewer raises the possibility that early regeneration involves transient cell cycle arrest in upper crypt progenitors, contributing to reduced villus cell output. This is an excellent hypothesis. We believe that there is a transient pause in cell contribution to the villus, during which dividing cells instead rebuild the crypt. BrdU staining (main text Figure 3c) shows that cells are actively proliferating at 56h post IR, and repopulate the crypts by 72 hours, without migrating up the villus (Figure R24, panel c). This proliferative behavior, combined with the absence of villus contribution at day 2 shown in main Figure 2g, supports our hypothesis.

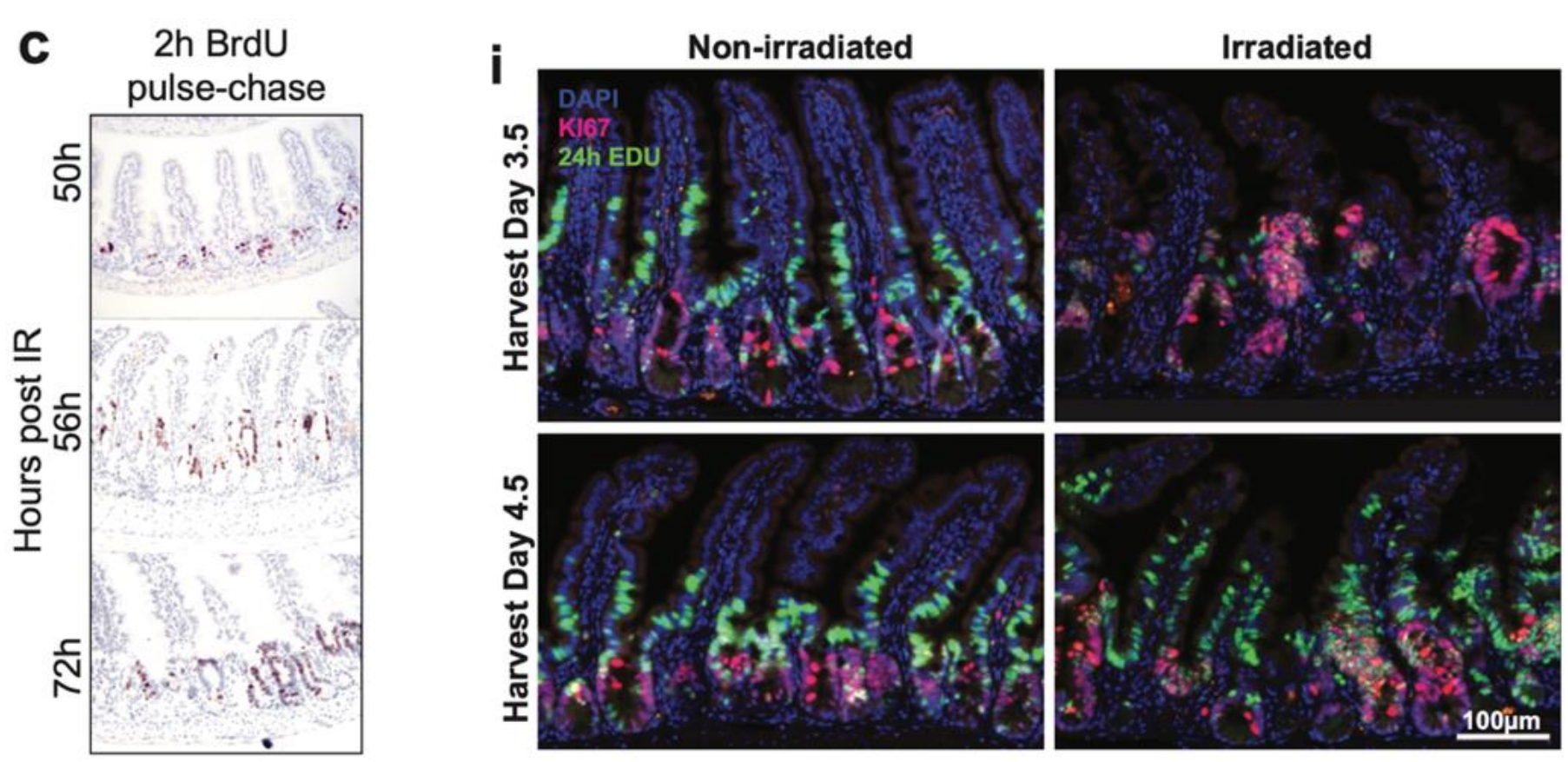

Figure R24: (main Figure 3c) BrdU labeling identifies actively proliferating cells at different timepoints following IR. At 50h, early proliferative cells appear at the crypt tops followed by crypt repopulation, while no signal is seen in the villus at these timepoints.
main Figure 2g: EdU staining reveals retention of proliferative epithelial cells in lower villus and crypt regions between days 2.5-3.5 post-IR (upper right box), followed by resumed upward migration by day 4.5 post-IR (bottom right box). This pattern suggests a delay in contribution of new cells to the villus base during regeneration. n=2.

In previous work (Chen et al., *Cell Stem Cell*), we performed a BrdU pulse-chase assay in which BrdU was injected on each day post-IR and tissues were harvested 24 hours later. As shown below (R25), day 2 and day 3 post-IR tissues show no progression of BrdU-labeled cells into the villi; instead, proliferative cells expand downward from the upper crypt.

[FIGURE REDACTED]

Figure R25: 24h BrdU pulse-chase immunostaining shows loss of proliferative marker (BrdU) incorporation on the villus between days 2-3 post-IR. On day 5 post-IR proliferation resumes at a faster rate as demonstrated by an increase in BrdU incorporation relative to day 0 control. n=3. Chen et al., *Cell Stem Cell.* 2023

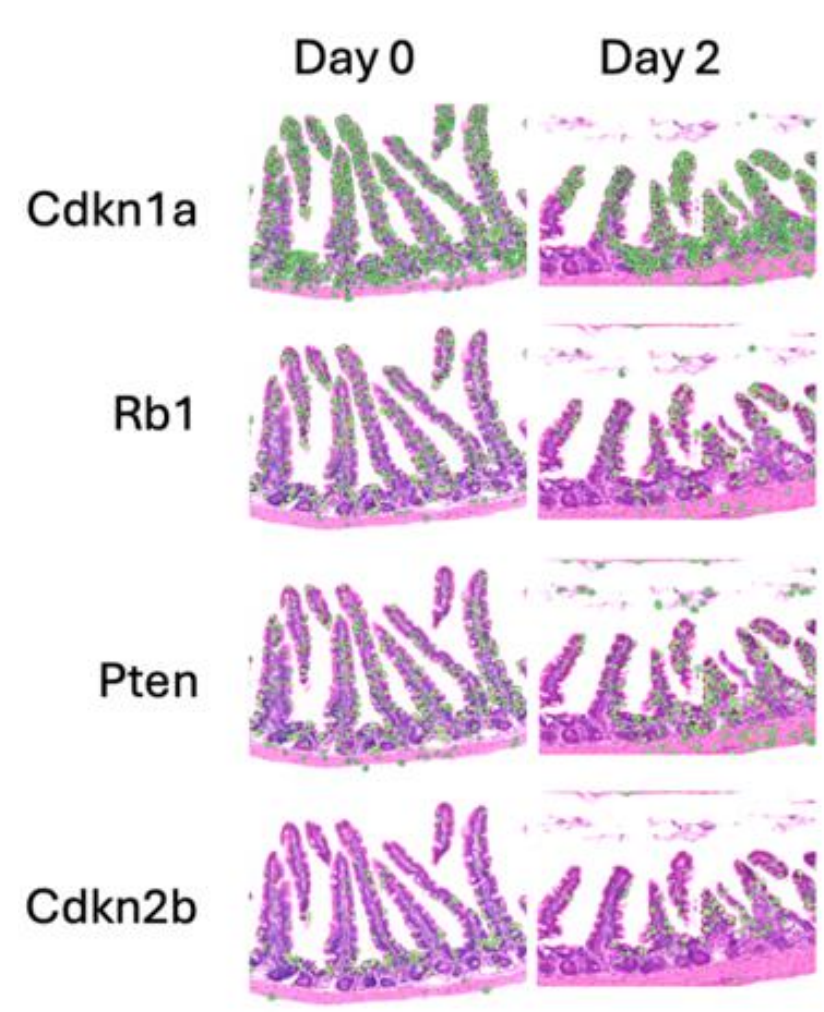

To more directly assess cell cycle arrest as the reviewer suggests, we also investigated the expression of cell cycle arrest genes in our spatial data. However, as shown below in Figure R26, there is no marked increase in cell cycle arrest gene expression on day 2. We therefore think that follow-up studies showing protein level changes may be the best way to further test the hypothesis proposed by the reviewer, and we are thankful for this suggestion.

Figure R26: expression of cell cycle arrest genes in the spatial data at days 0 and day 2 post IR. No dramatic changes observed.

In Figure 3, the authors' analysis showed normal and regenerative stem cell dynamics. Could the authors include D0 in their analysis (Figure 2d, e)? Did they observe the reduction of cell

numbers associated with homeostatic, stem cells-associated gene expression in D2 compared to D0?

This is a great suggestion; we now include day 0 timepoint in the analysis of gene signature activity (revised Figure 3e; includes days 0,1 and 5). The 'early regenerative' and 'late regenerative' clusters are absent at day 0 and emerge specifically following irradiation, supporting their injury-induced, regeneration-specific identity. Conversely, clusters associated with day 0 crypts are either completely lost or substantially reduced in day 2. We have also added a new figure panel (Revised Figure 3k) showing GSEA analysis, which demonstrates enrichment for *Lgr5*$^{+}$ stem cell gene set (Munoz et al., 2012) in the late-regenerative cluster and spheroid-enriched fetal gene set (Mustata et al., 2013) enrichement in the early-regenerative cluster. We hope this expanded presentation clarifies the relationship between homeostatic and regenerative stem cell dynamics across the injury-regeneration time course.

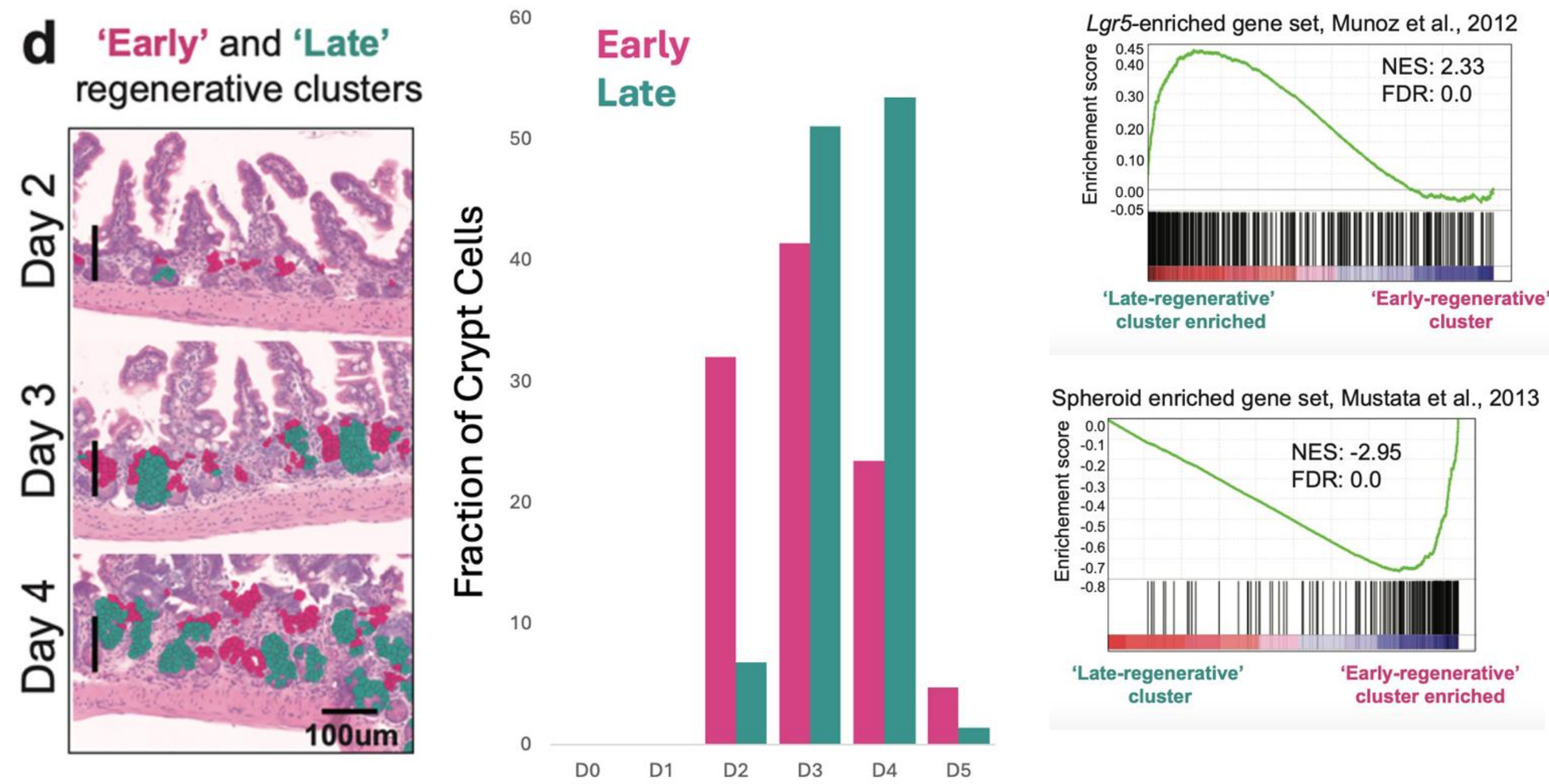

Figure R27: main Figure 3d-e and Figure 3k (GSEA analysis). Spatial distribution and temporal dynamics of early and late regenerative epithelial clusters following irradiation injury. (Left) Representative VisiumHD spatial maps overlaid on H&E-stained small intestinal sections at day 2, day 3, and day 4 post-IR, showing the localization of early regenerative and late regenerative cell clusters within the crypt compartment. (right) revised main Figure 3e: quantification of early and late cluster fractions as a percentage of total crypt cells at day 0-5. (Middle) Bar graph showing the fraction of crypt cells occupied by early and late regenerative clusters across all timepoints (D0-D5). Early cluster peaked at day 3 and largely resolved by day 5; late cluster emerges at day 2, and peaks at day 4. New Figure 3k: GSEA enrichment plots at day 3 post-IR comparing the early-regenerative and late-regenerative crypt subclusters. The *Lgr5*$^{+}$ stem cell gene set (Munoz et al., 2012) is enriched in the late-regenerative cluster (NES = 2.33, FDR = 0.0); the spheroid-enriched fetal gene set (Mustata et al., 2013) is enriched in the early-regenerative cluster (NES = -2.95, FDR = 0.0). FDR values were calculated using GSEA v4.3.3.

Hypertranscription is known to occur in adult stem cells and progenitors. Did the authors observe this in newly differentiated villus cells at later stages? If so, this would suggest that

hypertranscription might play additional roles (more than simply fueling the increased growth demands during regeneration).

This is a great suggestion. Yes! As shown below, there are increased mRNA counts across the villi in the regenerative time points compared to the control cells ranging from the lower villus and crypt-villus junction through the tip epithelial cells. The red and teal labeled data from day 3 and day 5 show higher mRNA counts than the other timepoints for these cells. However, as expected, the total count level in these cells is less than in the crypts and transit-amplifying zone where we believe hypertranscription is more prominent, and decreases as the cells approach the tip. The relative change is also lower as the cells ascend the crypt-villus axis, with the most notable difference against D0 being in the crypts and proliferative epithelial cells (as shown in main text Figures 4d-f and Supplemental Figure 5b-d compared to the ones below- R28).

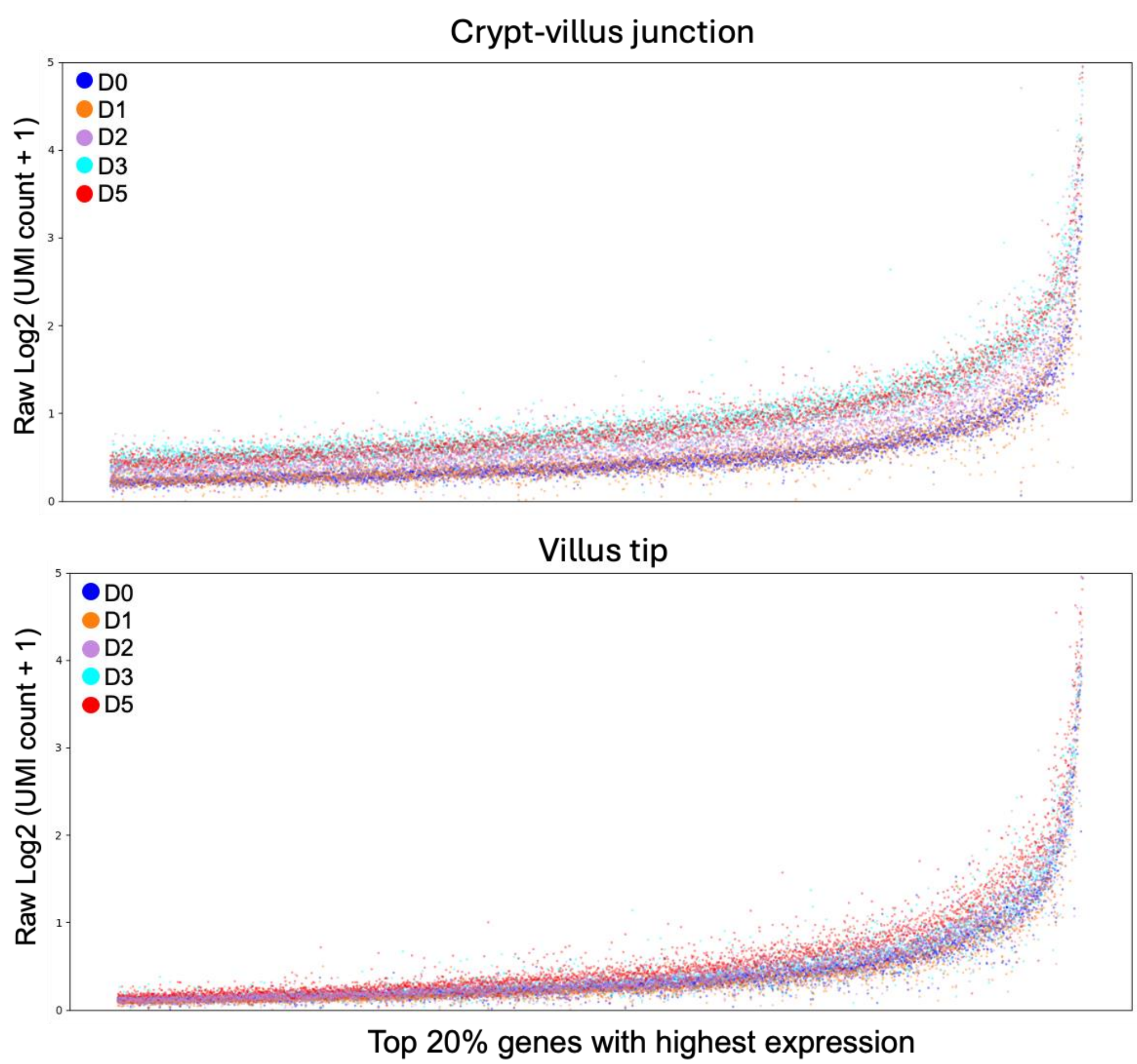

Figure R28: Crypt-villus junction cluster of replicate 2 shows global amplification of the transcriptome (top) while the phenomena is less obvious in the villus tip cluster (bottom). Amplification of the transcriptome shown as mean log_2-transformed raw UMI counts of the top 20% most highly expressed genes (individual genes are plotted on the x-axis) at the indicated timepoints in spatial data of Replicate 2 (top) crypt-villus junction or (bottom) villus tip clusters.

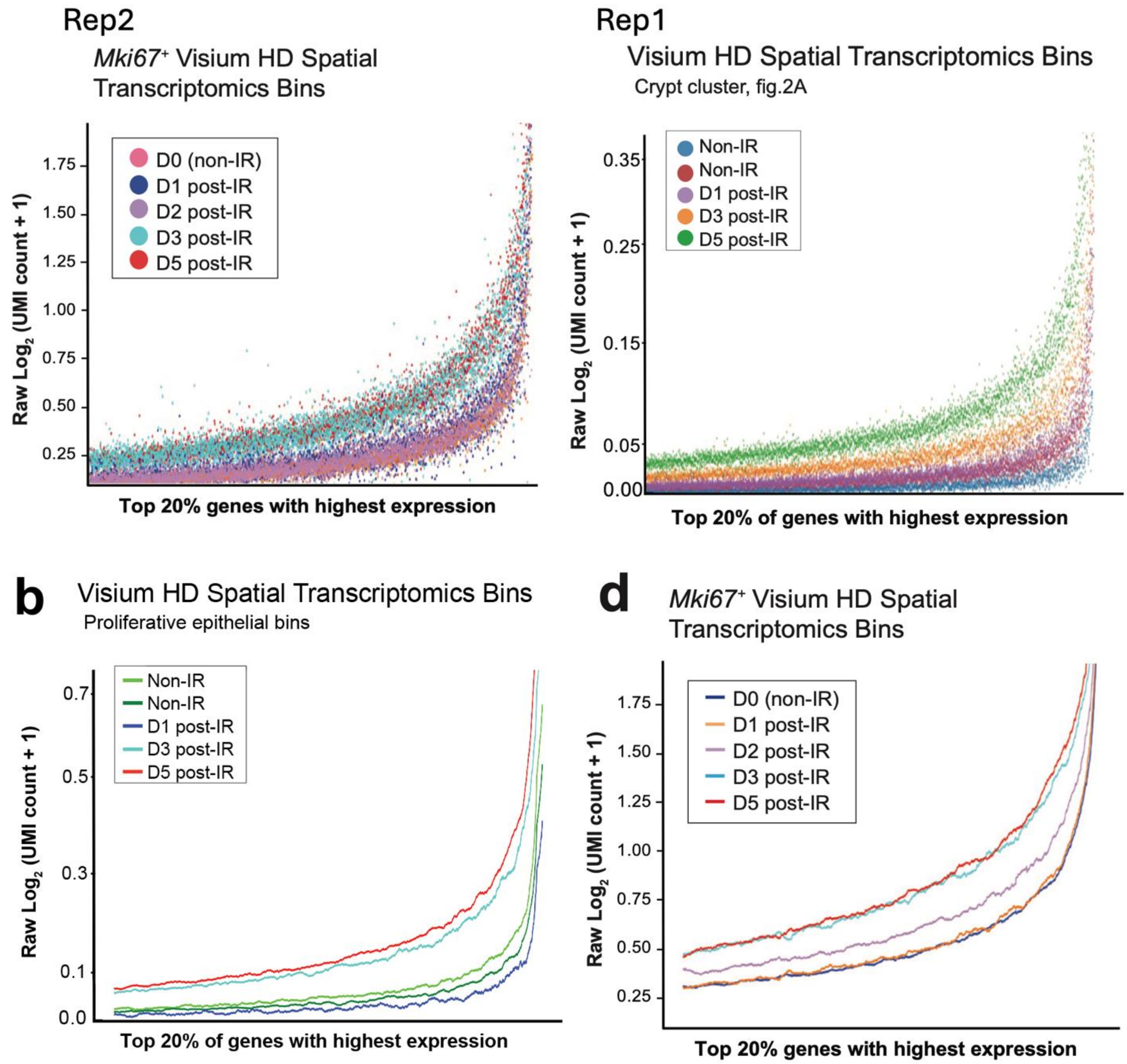

Figure R29: Global amplification of the transcriptome observed in regenerating tissues. Mean log_2-transformed raw UMI counts of the top 20% most highly expressed individual genes are plotted across timepoints in spatial data of (left) Replicate 2 filtered for proliferative epithelial bins (*Mki67*$^+$ and either *Epcam*$^+$ or *Vil1*$^+$) (revised Figure. 4d); and Replicate 1 (right). Data is limited to bins assigned to the crypt cluster based on unsupervised graph-based spatial clustering (revised Supplemental Figure 5b).

Therefore, we believe that these increases in transcripts in the villus epithelium are the result of elevated mRNA levels from their more proliferative cell progenitors. This is then exacerbated following hypertranscription, creating larger mRNA pools and increased count differences on day 3 and day 5 post-IR villi.

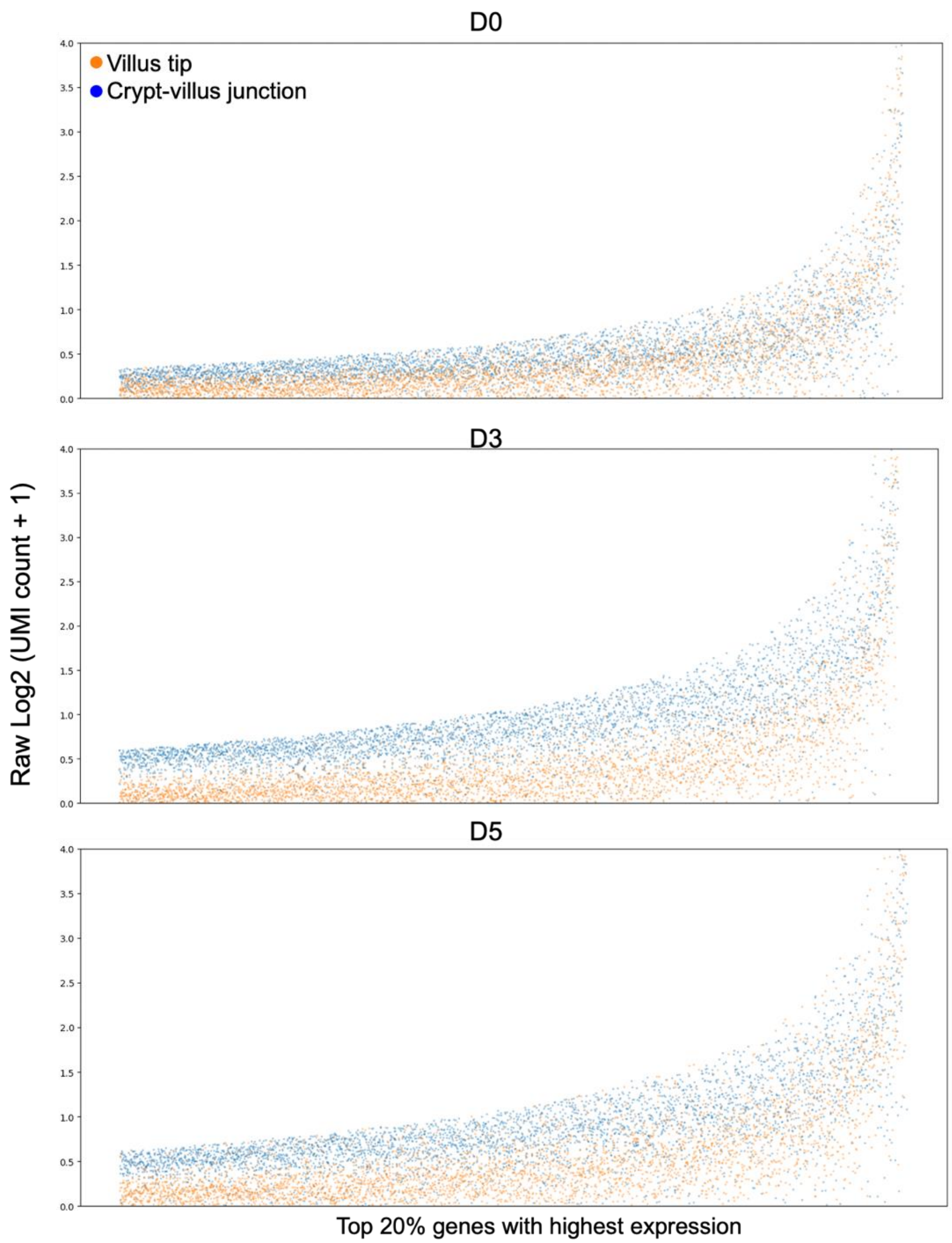

Figure R30: Broad amplification of the transcriptome shown as mean log_2-transformed raw UMI counts of the top 20% most highly expressed individual genes are plotted across timepoints in spatial data of Replicate 2 for days 0, 3 and 5, comparing villus tip cluster to crypt-villus junction cluster shows global amplification most apparent in crypt-villus junction on day 3 and 5 post-IR.

There may be additional roles for these interesting transcriptional increases in these differentiating cells in the regeneration timepoints, but we feel like we cannot make solid claims about these roles at this point of our studies and thus focused on highlighting the hypertranscriptional pattern in the regenerative cell populations closer to the crypt for our initial work. It will be exciting to see if the RNA abundance has additional roles in differentiated cells in the future.

The authors showed that TGFB expression in crypt stroma is enriched during regeneration (Figure 5i). Which stromal cell types do express TGFB? Did % of cells expressing TGFB also increase?

TGFB1 is largely expressed in stromal cell populations, in regions both surrounding the crypts and throughout the villi following IR. We have conducted additional analysis to parse this question and find there is relatively minimal expression in the epithelial populations based upon psdeudo-single cell analysis (see below). In the regenerative tissue, the proportion of stromal cells expressing *Tgfb1* increases at day 2-day 5 post-IR relative to day 0, with similar proportions between the control and day 1.

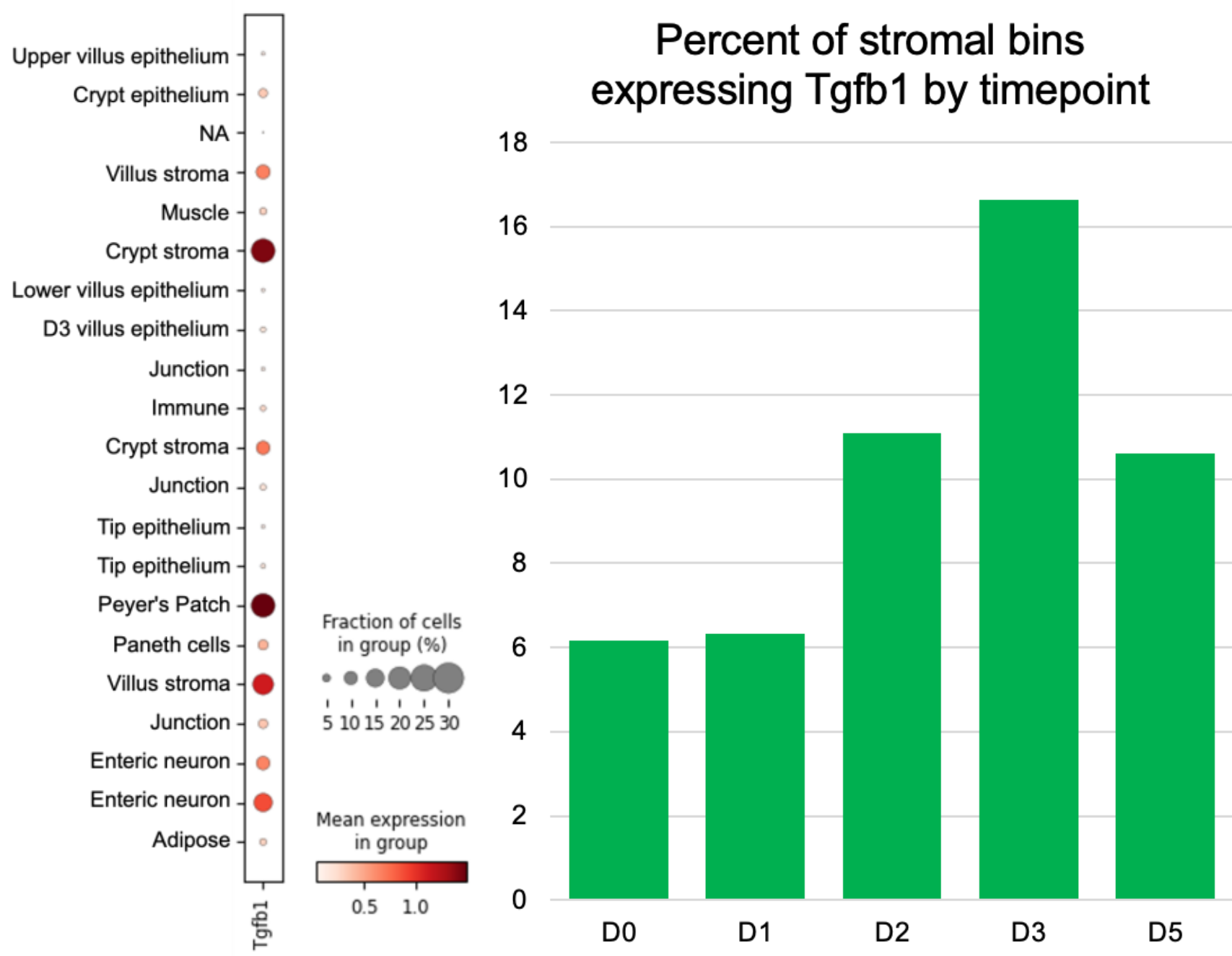

Figure R31: pseudo-single cell analysis of graph-based clusters of the spatial data shows *Tgfb1* expression high in stromal bins relative to epithelial bins (left); percent bins expressing *Tgfb1* by timepoint (right).

Below (R32), we also show that both *Tgfb1* and *Tgfbr2* expression are induced around the crypt and crypt-villus junction areas during these regenerative timepoints, aligning with main text Figure 5l. Providing an additional spatial context to the expression of these important pro-regeneration genes.

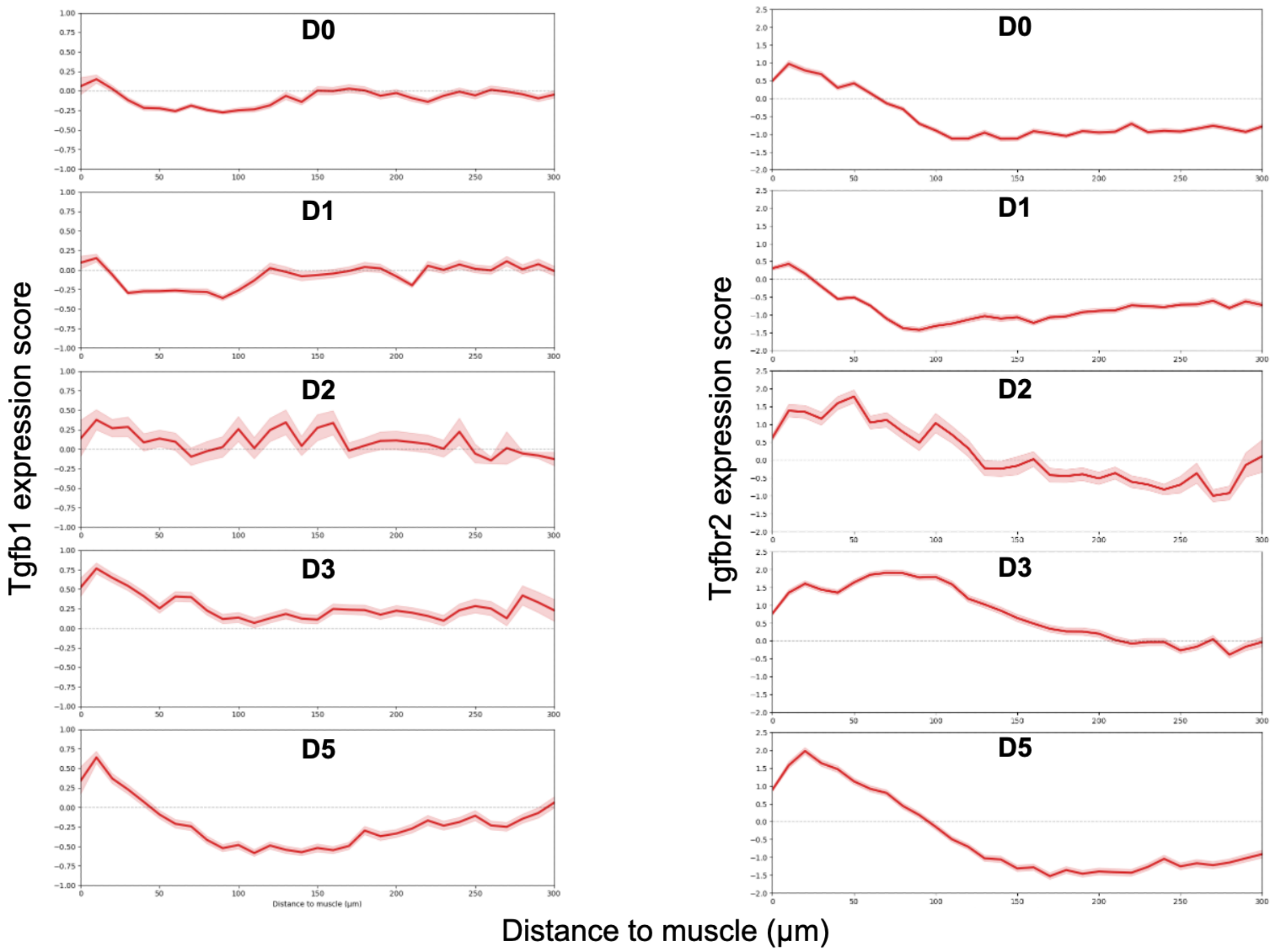

Figure R32: Scored expression of the average expression of Tgfb1 (left) and Tgfbr2 (right) in each of the timepoints (days 0,1,2,3, and 5) at distances away from the smooth muscle.

It appears that the expression may largely be driven by immune populations such as monocytes/macrophages that infiltrated the crypt stromal area. We have previously investigated TGFB’s role in the regenerative process (Chen et al., *Cell Stem Cell* 2023), where we demonstrated that *Tgfb1* is co-expressed with monocyte/macrophage markers. The UMAP of the scRNA-seq from this previous study support the conclusion that *Tgfb1* is being made by macrophages and fibroblasts is shown below. This previous study only showed limited details of

[FIGURE REDACTED]

the spatial patterning of these expression trends; the current work allows us to elaborate upon the spatial contexts of the signaling.

Figure R33: scRNA-seq UMAP of all intestinal cells identifies Tgfb1-high cluster co-expressing markers of monocytes/ macrophages. Figure extracted from Chen et al., *Cell Stem Cell* 2023, data analyzed GSE165318).

We thank the reviewer for this suggestion, as it illustrates the utility of our spatial atlas of intestinal regeneration as a resource for the field.

Reviewer #3 (Remarks to the Author):

This manuscript presents a comprehensive spatial and temporal transcriptomic analysis of intestinal regeneration following irradiation-induced injury in the mouse jejunum. Overall, the study is carefully executed, technically sound, and provides a valuable community resource. The authors make effective use of high-definition spatial transcriptomics to preserve crypt–villus architecture and to resolve dynamic transcriptional changes across epithelial compartments that would be difficult to capture with earlier Visium-based approaches. The inclusion of multiple biological replicates across time points, transparent presentation of replicate-level data, and experimental validation strengthen the robustness of the conclusions.

We sincerely appreciate that the reviewer found our work comprehensive and containing robust conclusions.

While many individual observations, such as crypt–villus zonation or interferon-responsive programs, have been reported previously using alternative methods, this work represents, to my knowledge, the first systematic description of intestinal regeneration using HD spatial transcriptomic technologies. The logical progression of the manuscript, moving from villus (Figure. 1) to crypt–villus junction (Figure. 2), to crypt (Figure. 3), followed by transcriptional amplification (Figure. 4) and cell–cell communication (Figure. 5), provides a coherent and well-structured narrative. The integration of single-cell RNA-seq datasets and extension of hypertranscriptional findings to human data (Figure. 4) is particularly compelling.

We also appreciate the kind words that the work is systematic, logical, and a coherent and well-structured narrative.

That said, there are conceptual, analytical, and presentation-related issues that should be addressed to improve clarity, coherence, and suitability for being published in Nat Comm.

We also thank the reviewer for taking the time to suggest improvements, which we hope we have adequately addressed and believe have substantially improved the revised manuscript.

Major Comments
1. Throughout the manuscript, the authors alternate between different clustering approaches (K-means vs. graph-based clustering) and spatial units (8-bin segmentation vs. cell segmentation) without clear justification. This is particularly confusing in Figure 1 (panels c–g) and Figure 5, where different strategies appear to be used within the same Figure.
o The authors should clearly explain the rationale for each clustering method, specify which approach is used for downstream analyses, and ideally standardize the strategy across Figures to improve consistency and interpretability.

This is an important point. To reduce any confusion, we have revised the manuscript to improve clarity and standardization. All primary analyses have been updated to use cell segmentation as the spatial unit where possible, and the methods has been updated specifically noting which analyses used the 8 um bins. Cell segmentation requires high-resolution imaging and sufficient sequencing depth; accordingly, replicate 2 was deliberately selected as the representative high-depth replicate to enable cell-level analysis. Replicates 1, 3, and 4 were acquired at standard sequencing depth and were analyzed using 8-bin spatial segmentation and corroborate the findings. Critically, the spatial patterns and cluster identities observed across Replicates 1, 3, and 4 were highly concordant with those from the cell-segmented Replicate 2, confirming that our conclusions are robust across both segmentation strategies and sequencing depths. A new table is also included to provide statistics on the experiment (revised Supplemental Figure 1b-d). We thank the reviewer for this important suggestion and feel the revised manuscript is improved as a result.

Cell segmentation: The table below describes the number of segmented cells for the segmented data per timepoint.

Barcode: single cells identified by 10x Genomics Space Ranger v4.0 cell segmentation

Timepoint	Day 0	Day 1	Day 2	Day 3	Day 4	Day 5
Rep2, Cell segmented	39,214	31,306	8,233	39,314	2,927	38,785

Figure R34 (new Supplemental Figure 1d): segmented cell count per timepoint for cell segmented replicate 2 data.

In the cell segmented data, a barcode represents a single cell as identified by 10x Genomics Space Ranger v4.0 cell segmentation, in which nuclei are first detected from the H&E image

using a StarDist deep learning model, and cell boundaries are then inferred by expanding outward from each nucleus to assign surrounding 2 μm bins. More on this method can be found on 10x Genomics webpage.

In the 8um binned data, a barcode represents an 8x8 um square, comprised of sixteen 2um bins. The table below describes the number of 8x8 um square barcodes per timepoint for each of the replicates for the 8um bin data:

Barcode: 8 × 8 μm square, comprised of sixteen 2 μm bins.

Timepoint	Day 0	Day 1	Day 2	Day 3	Day 4	Day 5	Day 6
Rep1	110,915*	65,580		89,965		72,168	
Rep2	82,366	70,405	26,653	84,043	7,276	85,695	
Rep3	97,387	82,219	24,661	68,233	8,436	99,601	
Rep4	91,960		68,616*		15,2811*		98,240

* 2 replicates combined

Figure R35 (new Supplemental Figure 1c): barcode count for 8um bin data per timepoint within each of the four replicates.

We also updated the previous table (New Supplemental Figure 1b):

Timepoint	Unique reads	Mean reads per 8μm bins	Mean UMI per 8μm bin	2um binned squares
Rep1	328,601,250	791.3	144.1	6,644,192
Rep2	1,126,204,763	2834.3	1189.4	6,357,536
Rep3	513,318,882	1288.5	713	6,374,112
Rep4	831,962,821	1920.2	1107.4	6,308,240

Figure R36: new Supplemental Figure 1b.

2. In Figure 2b, the authors describe the emergence of a new cluster at the crypt–villus junction at day 3 post-irradiation. However, macrophage recruitment is emphasized in Figure 2a, while macrophage or monocyte contributions to the newly emerging cluster are not clearly demonstrated.
o The biological identity and relevance of this new cluster should be clarified. Additional marker analysis or functional interpretation would strengthen the claim that this cluster is regeneration-specific and biologically meaningful.

We thank the reviewer for this observation and apologize for the confusion. The ambiguity arose from transitioning directly from macrophage recruitment data (Main Figure. 2a; Figure R37) into cluster dynamics (Main Figure. 2b; Figure R37) in the text without explicitly separating these as independent observations.

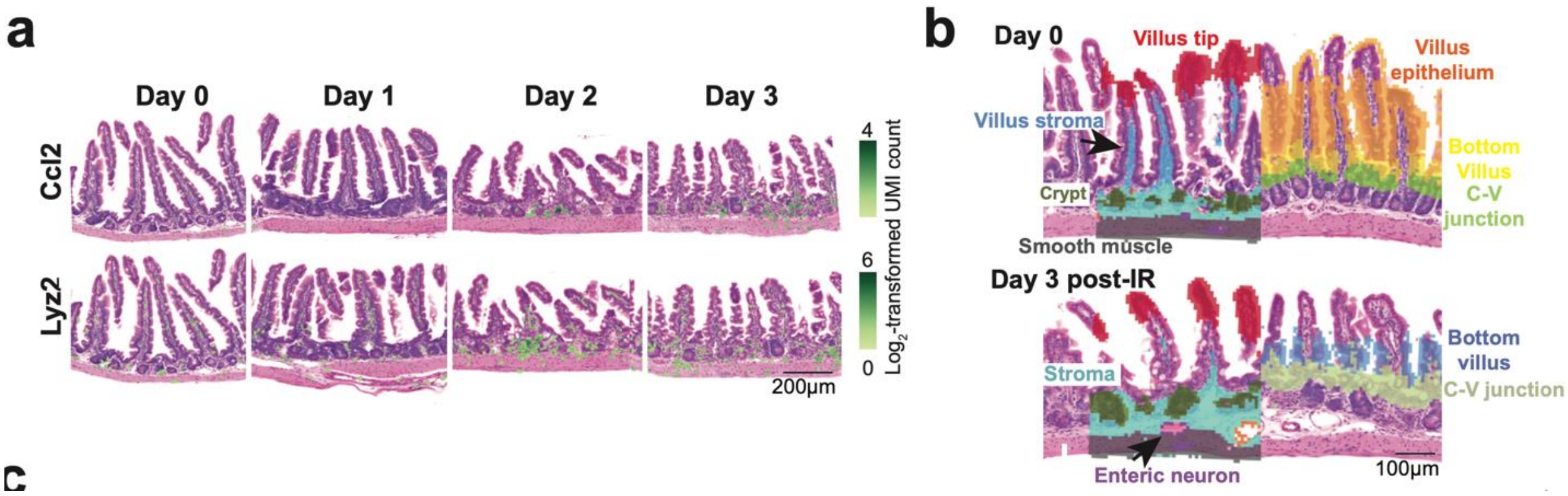

Figure R37: previously main text Figure. 2a and Figure. 2b referenced in the reviewers' comments.

To clarify what we intended to show, the emergent crypt-villus junction cluster at day 3 post-irradiation represents a transcriptionally distinct epithelial population, not a macrophage or immune cluster. We have improved the writing in the revised manuscript to clarify and hopefully our readers will now be spared this miscommunication.

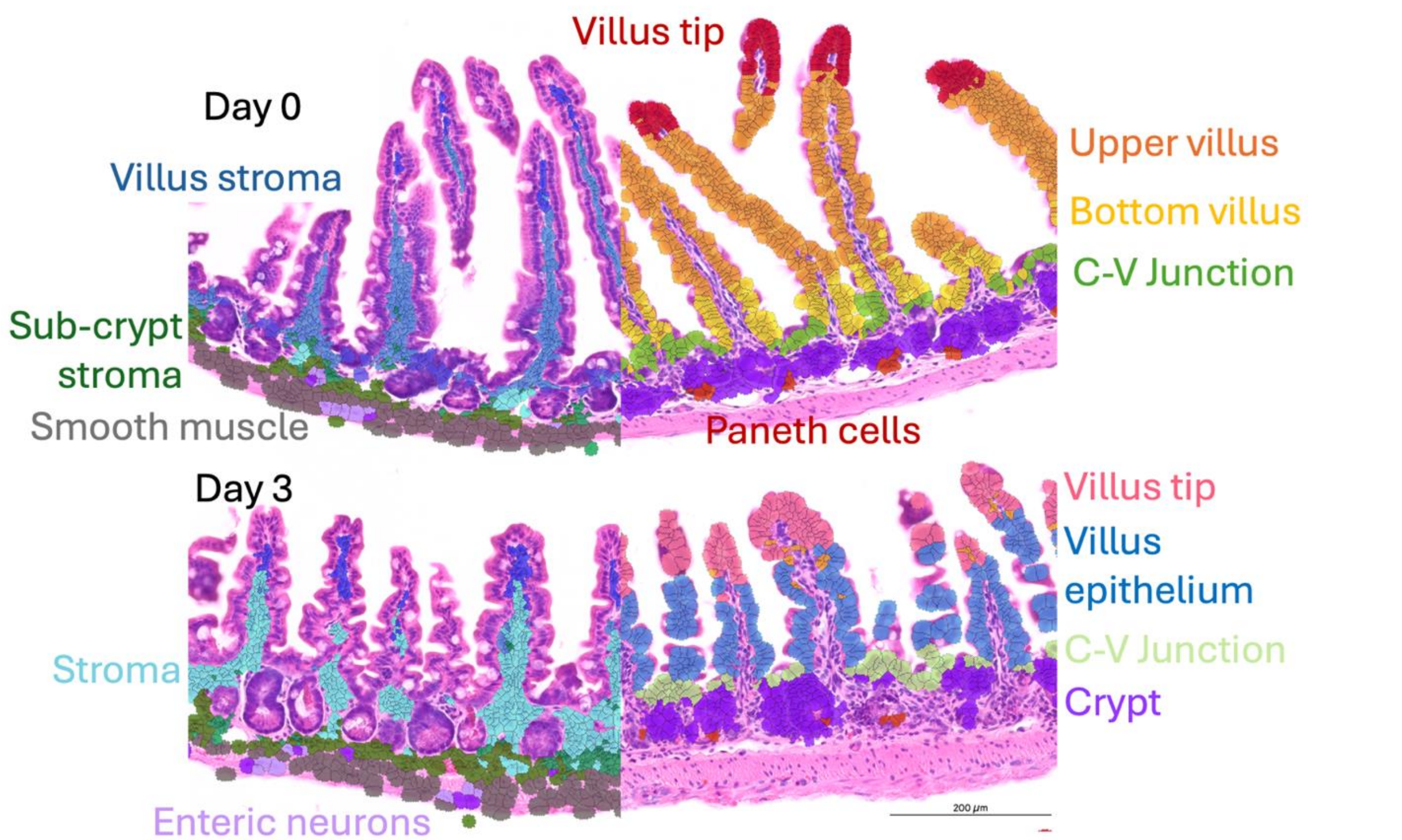

Figure R38: graph-based clusters generated in the cell segmentation data; on the left are the non-epithelial clusters and on the right are the epithelial clusters.

We also performed additional analysis as suggested by the reviewer:
"The biological identity and relevance of this new cluster should be clarified." The spatial transcriptomics data show that cells at the C-V junction epithelium undergo sufficient transcriptional changes during regeneration to be reassigned to new cluster identities (shown in the Figure below as mint colored cluster on day 3).

To characterize the junction cluster more explicitly, we performed differential gene expression analysis comparing the day 0 versus day 3 junction clusters (adj. P< 0.05, MNA > 0.03, log2FC > 1). The day 0 cluster is defined by canonical absorptive cell programs (lipid metabolism, oxidoreductase activity, and mitochondrial function), while the day 3 cluster shows strong upregulation of regeneration- and fetal-like genes, including *Areg*, *Anxa1/8/10*, *S100a6/11*, and *Duoxa2*, with GO term enrichment for innate immune response, response to bacterium, cell division, and apoptosis.

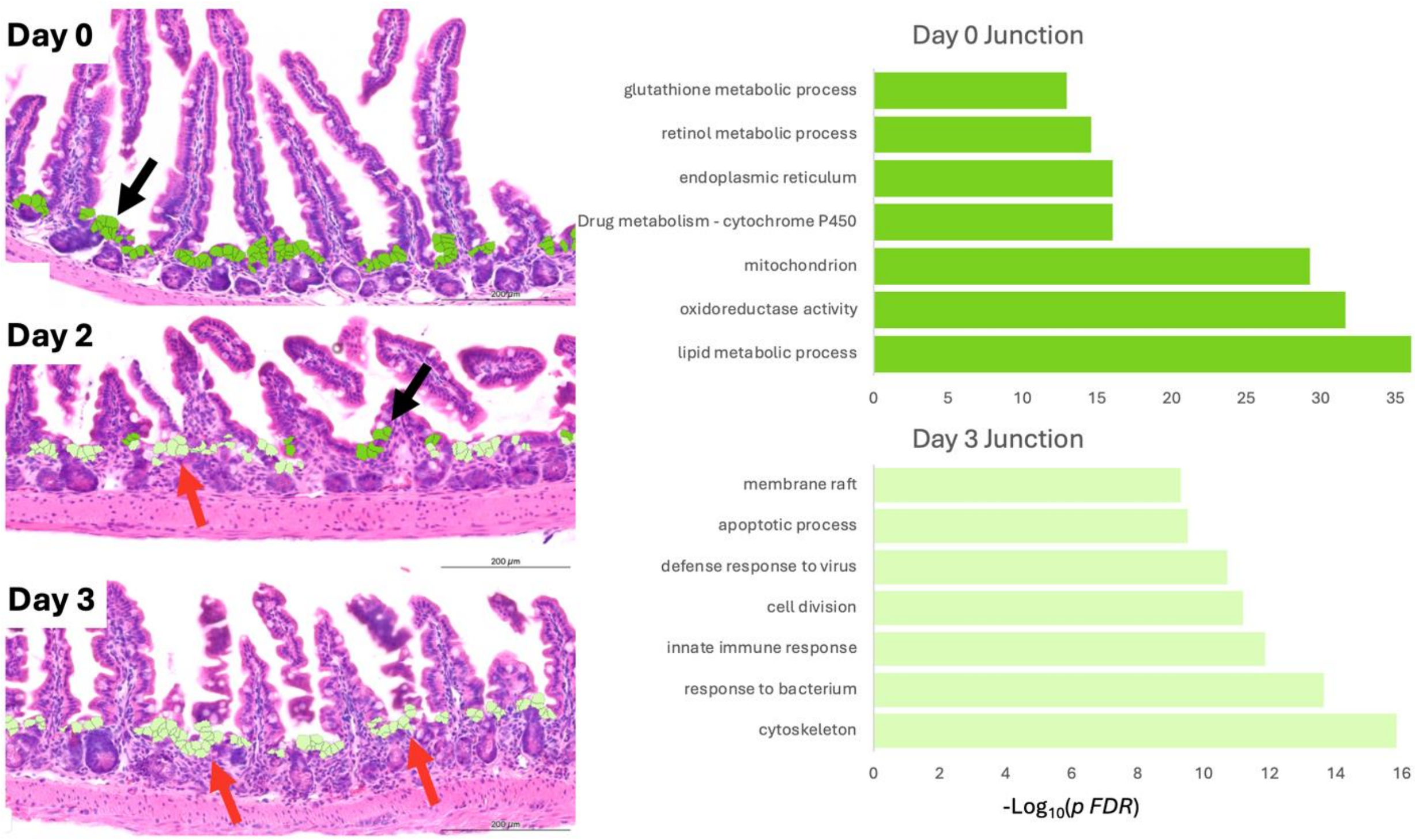

Figure R39: (left) Day 0 C-V junction is marked by a green cluster and black arrows, on day 3 we see a new cluster shown in mint and red arrows. These clusters both appear on day 2; (right) GO term enrichment analysis for both C-V junction clusters of day 0 (top) and day 3 (bottom),

As an unrelated point, macrophage markers *Ccl2* and *Lyz2* (Figure. 2a, shown above) were included in the original text as an illustrative example of cell-type-specific spatial dynamics. These genes are primarily expressed in the stromal clusters and are separate from the epithelial crypt-villus junction mentioned above. Macrophage biological relevance is supported by our own work and others demonstrating a functional role for mucosal macrophages in intestinal regeneration (Ruder & Becker, 2020; Chen et al., 2023; Moraitis et al., 2025) and was not intended to imply macrophage contribution to the newly formed epithelial cluster.

We thank the reviewer for alerting us to this confusing part of our manuscript and we have revised the manuscript text accordingly to avoid confusion about these independent points.

3. The comparison of differential gene expression between "bottom villus day 0" and "bottom villus day 3" is based on two different clusters (green vs. yellow). Since these regions are defined by distinct clusters, transcriptional differences are inherently expected, making it difficult to disentangle temporal effects from cluster identity effects.
o A more informative analysis would include comparisons of the same cluster across time points, or a direct comparison between the green and yellow clusters across all samples, followed by interpretation of their temporal replacement.

This is a valid point. Our original comparison of villus bottom cells between timepoints was intended to capture how the lower villus geographical region changes during intestinal regeneration. We agree that additional comparisons can be made and that these offer further insights, consistent with the value of these data as a resource. We thank the reviewer for the suggestions, and we have performed two complementary analyses below. Related analyses requested by Reviewer 1 are also addressed in Reviewer 1, Question 3.

"A more informative analysis would include comparisons of the same cluster across time points"

Analysis 1: same cluster compared across day 0 and day 3

Because the graph-based clustering (k=21) does not generate a shared villus cluster across all timepoints, notably, the green cluster is absent at day 3, we turned to k-means clustering generated by Space Ranger (k=10). K-means produces spatially defined clusters without prior knowledge of cell identity, making it well-suited for consistent cross-timepoint comparisons. The orange cluster (shown below, Figure R40) spans the lower villus epithelium across all time points and therefore serves as a consistent spatial reference for temporal comparison, as requested by the reviewer.

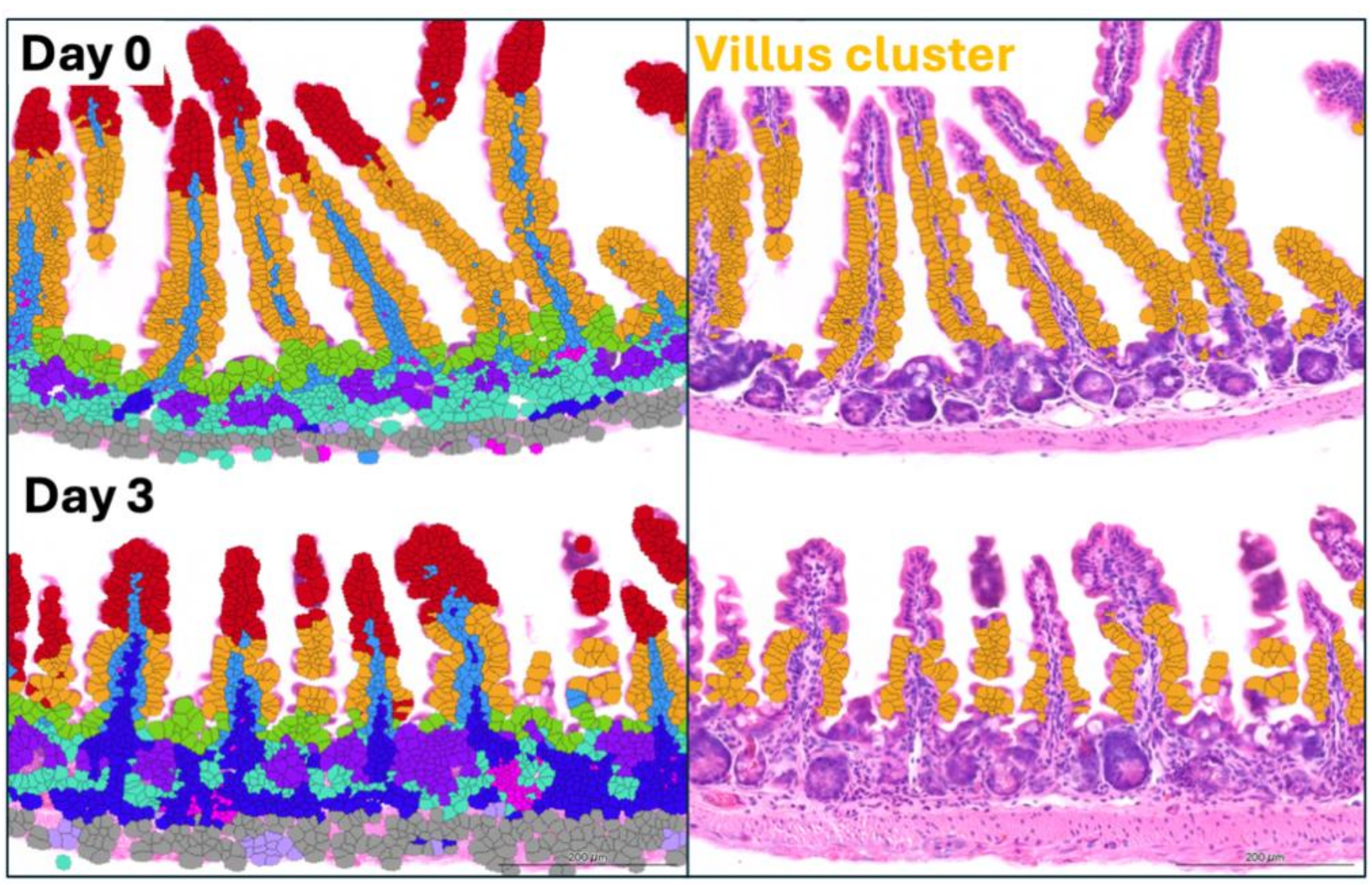

Figures R40: K-means (k = 10) clusters (left); orange villus cluster at day 0 (top) and day 3 (bottom) (right).

Differential gene expression analysis of the orange villus epithelium cluster between day 0 and day 3 identified 314 genes upregulated at day 0 and 273 genes upregulated at day 3 (adj.*P*<<0.05, MNA >0.03, log2FC>1). GO term analysis reveals distinct signatures at each timepoint.

At day 0, enriched terms include oxidoreductase activity, glutathione metabolism, and metabolic processes associated with homeostatic villus function. At day 3, enriched terms shift toward innate immune response and cell proliferation and migration, consistent with an active regenerative program. Please also see reviewer 1, question 3 for further related analysis. These results corroborate our previous findings and, following the reviewer's suggestion to examine these populations using an orthogonal approach, strengthen our confidence in the reported gene expression changes occurring in the lower villus.

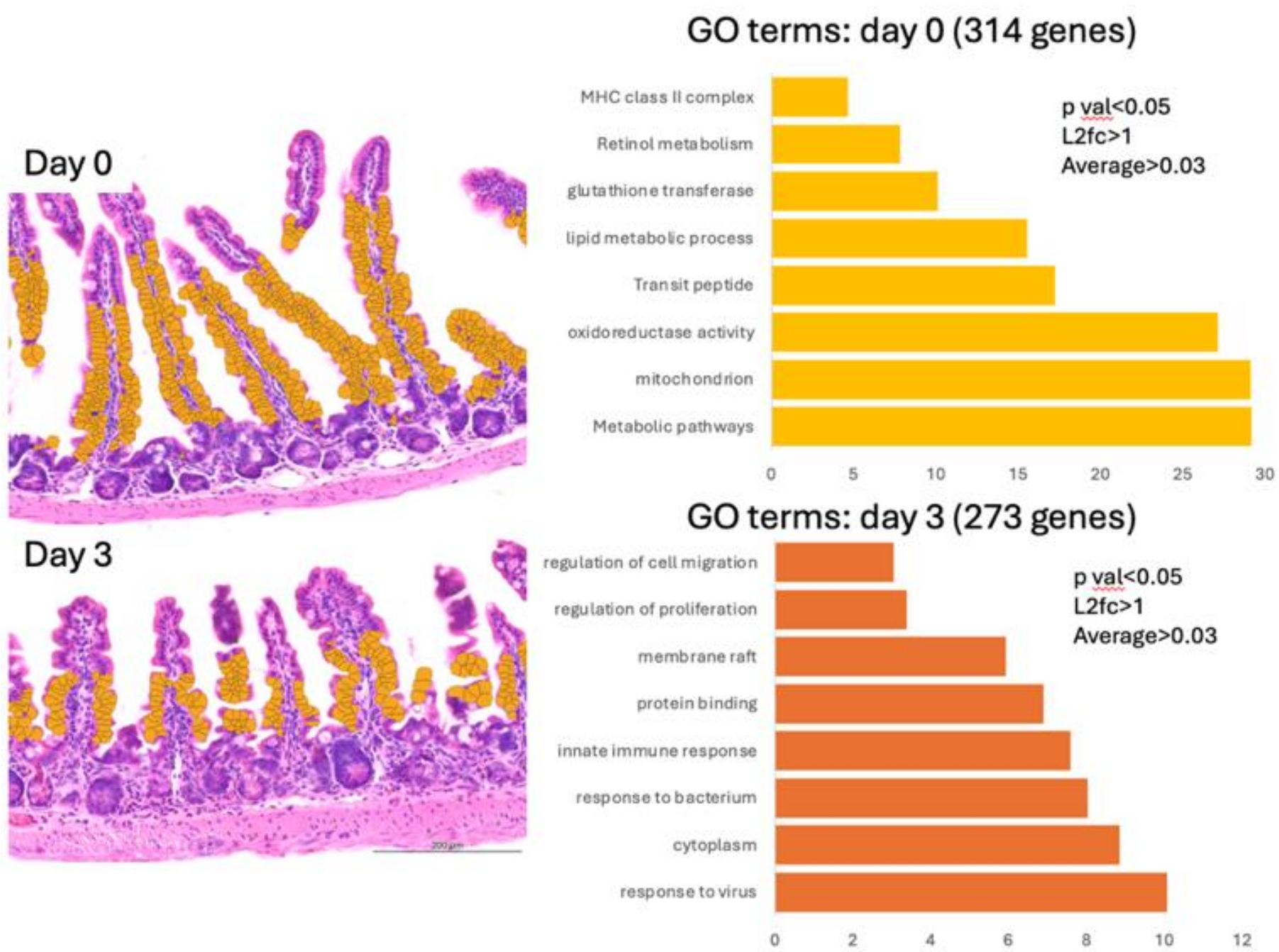

Figure R41: (left) shared villus epithelium cluster (k-means) at day 0 (top) and day 3 (bottom). (right) GO term enrichment for day 0 vs. day 3 within the shared orange villus epithelium cluster.

Analysis 2: Villus epithelium compared across all timepoints
We extended the analysis to compare the gene expression within the villus epithelium across all timepoints (days 0, 1, 2, 3, and 5), tracing the transcriptional trajectory of this region throughout the regenerative time course.

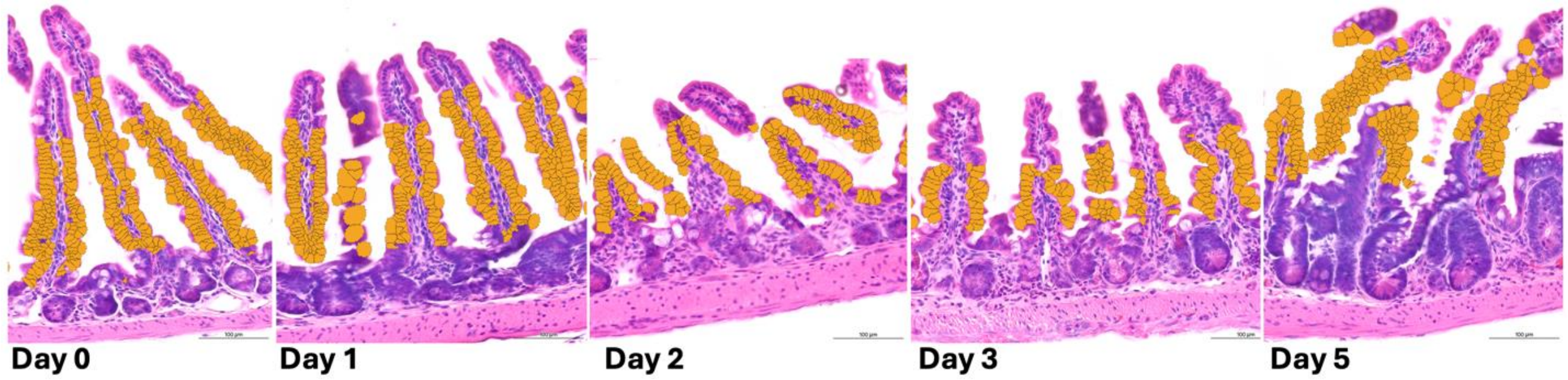

Figure R42: villus epithelial cluster (k-means) spanning across all timepoints

To characterize transcriptional changes in the villus epithelium across the injury-regeneration time course, we performed GO term enrichment analysis on differentially expressed genes at each timepoint ($log_2FC > 0.8$, MNA>0.03, adj.*P*<0.05; Figure R42 and R43. The number of differentially expressed genes was lowest at days 1 and 2, with more genes differentially expressed at days 3 and 5, reflecting the progressive transcriptional remodeling of the villus epithelium during regeneration. The top enriched GO term at Day 0 was MHC class II antigen presentation, consistent with homeostatic immune surveillance activity in the villus. At day 1, enrichment for chemical carcinogen and DNA adduct response terms reflects the acute genotoxic burden imposed by irradiation. Day 2 was characterized by N-linked asparagine biosynthesis, suggestive of early glycoprotein production associated with epithelial restitution. By day 3, defense response to virus was the most significantly enriched term, consistent with robust interferon-stimulated gene activity previously identified in spatially restricted villus bins. Day 5 showed enrichment for broad metabolic pathways, possibly reflecting a return toward biosynthetically active, mature villus epithelium.

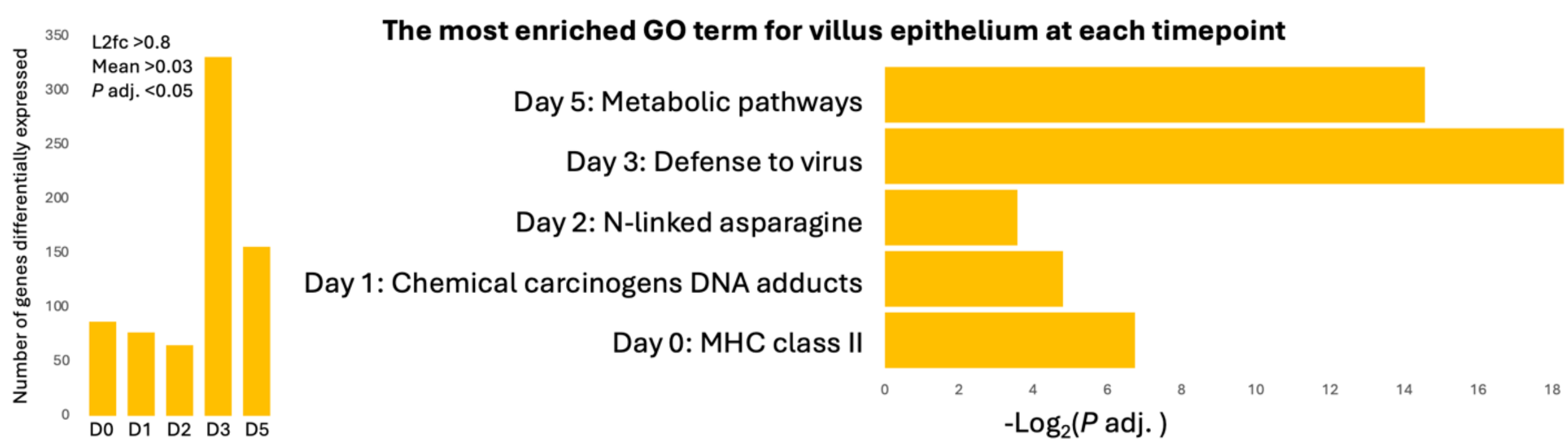

Figure R43: GO term enrichment analysis of differentially expressed genes in the villus epithelium across the injury-regeneration time course. (Left) Number of differentially expressed genes per timepoint ($log_2FC > 0.8$, MNA >0.03, adj.P<0.05). (Right) the most enriched GO term at each timepoint shown as $-log_2$(adj.*P*).

We thank the reviewer for prompting us to conduct these additional analyses which we think substantiate our original interpretation of the regenerative program and provide additional resolution on the dynamics of villi during recovery from IR-induced damage.

4. The study is restricted to the jejunum, yet the title refers broadly to “mouse intestinal regeneration.” Given known regional differences across the small intestine and colon, this title overgeneralizes the scope of the findings.

o The title and conclusions should be adjusted to reflect the jejunal focus, or additional regions should be included to support broader claims.

We thank the editor for this suggestion that will improve clarity about our study for readers. We have revised the title : **A Spatial and Temporal Transcriptomic Atlas of Mouse Intestinal Regeneration in the Jejunum.**

5. While the dataset is valuable, parts of the manuscript remain largely descriptive, particularly the discussion of Isg15^{+} villi (Figure. 1i). The proposed villus–villus communication is intriguing but underdeveloped, lacking mechanistic hypotheses or exploratory analyses (e.g., differences in cell-type composition or signaling states).
o Some deeper analytical insight or hypothesis-driven exploration would strengthen the manuscript's impact.

This is an important critique and reflects the interest in the observation about interferon signaling and the request for more analysis of this phenomenon. In the revised manuscript, we dig deeper into the potential of IFN signals spreading to adjacent villi at day 3. We hypothesized that spreading to adjacent villi could occur through the crypt-villus junction epithelium or through the crypt epithelium.

We focused on genes that were unique to regions associated with IFN-villus spreading vs. non-spreading and identified *Ifit2* as a gene with striking spatial restriction- present almost exclusively at the crypt-villus junctions of clustered *Isg15*$^{+}$ villi at day 3 and absent at day 0- suggesting it marks a damage-induced, junction-specific activation state correlating with signaling spreading rather than a general ISG response. Analysis comparing *Ifit2*$^{+}$ to *Ifit2*$^{-}$ junction bins at day 3 identified 42 differentially expressed genes enriched in the *Ifit2* spatially restricted population, making them candidates for functional involvement in the local IFN spreading response.

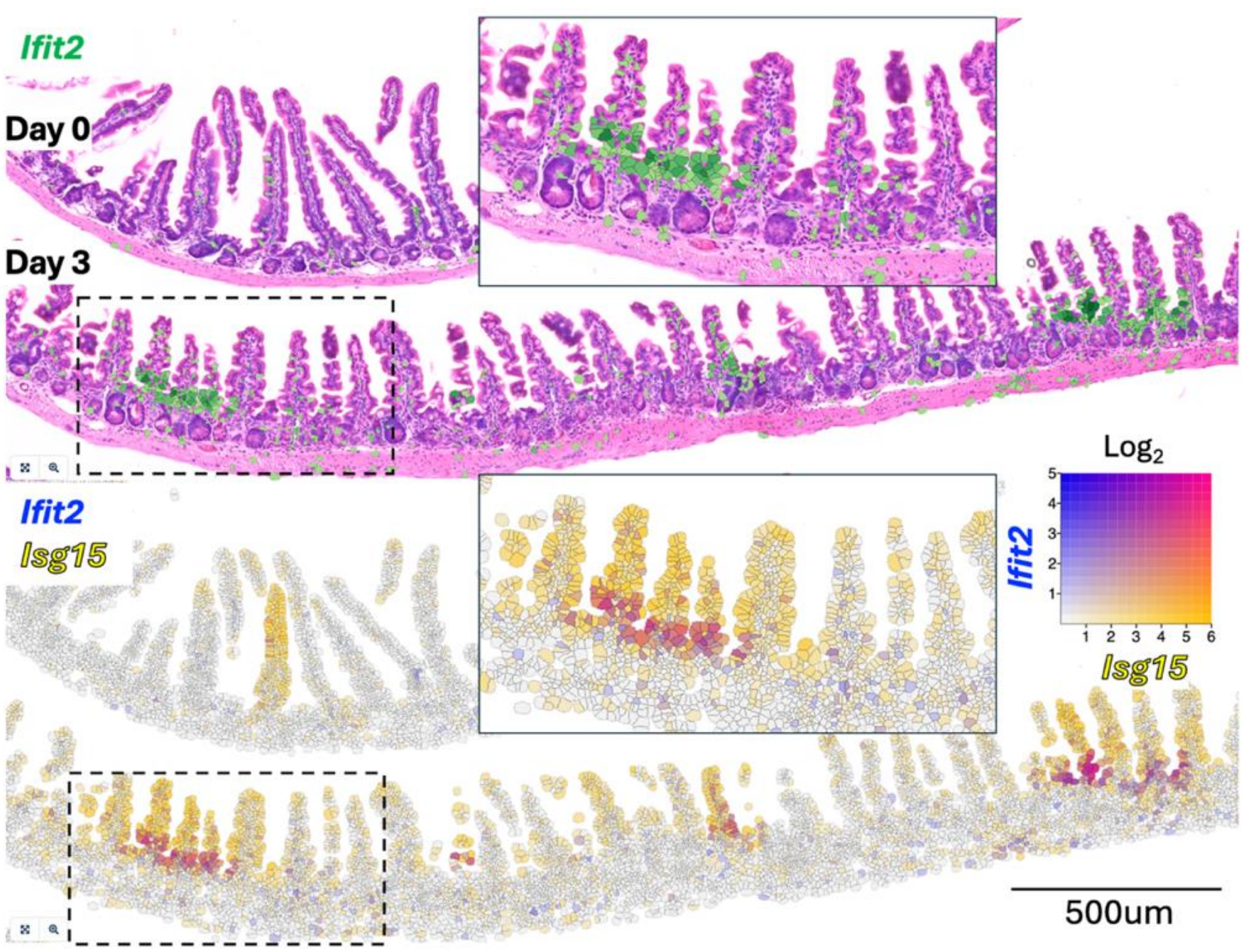

Figure R44 (new Supplemental Figures 2e and f): *Ifit2* active junction regions in rep2 day 0 and day 3 (top); *Ifit2* and *Isg15* co-expression (bottom).

Beyond the canonical ISG signature, the *Ifit2*$^{+}$ cell gene list includes Schlafen family members (*Slfn2*, *Slfn4*, *Slfn5*), known mediators of IFN-driven growth inhibition; *Usp18*, a negative regulator of Type I IFN signaling that may indicate a local feedback mechanism; *Duoxa2*, which has roles in oxidative antimicrobial defense; and *Cxcl10*, raising the possibility of paracrine immune recruitment. The full gene list is provided in Supplementary Table 3. Together, these genes represent spatially informed hypotheses for the functional state of junction-restricted IFN-active cells during regeneration, and we identify this population as a compelling candidate for future mechanistic studies.

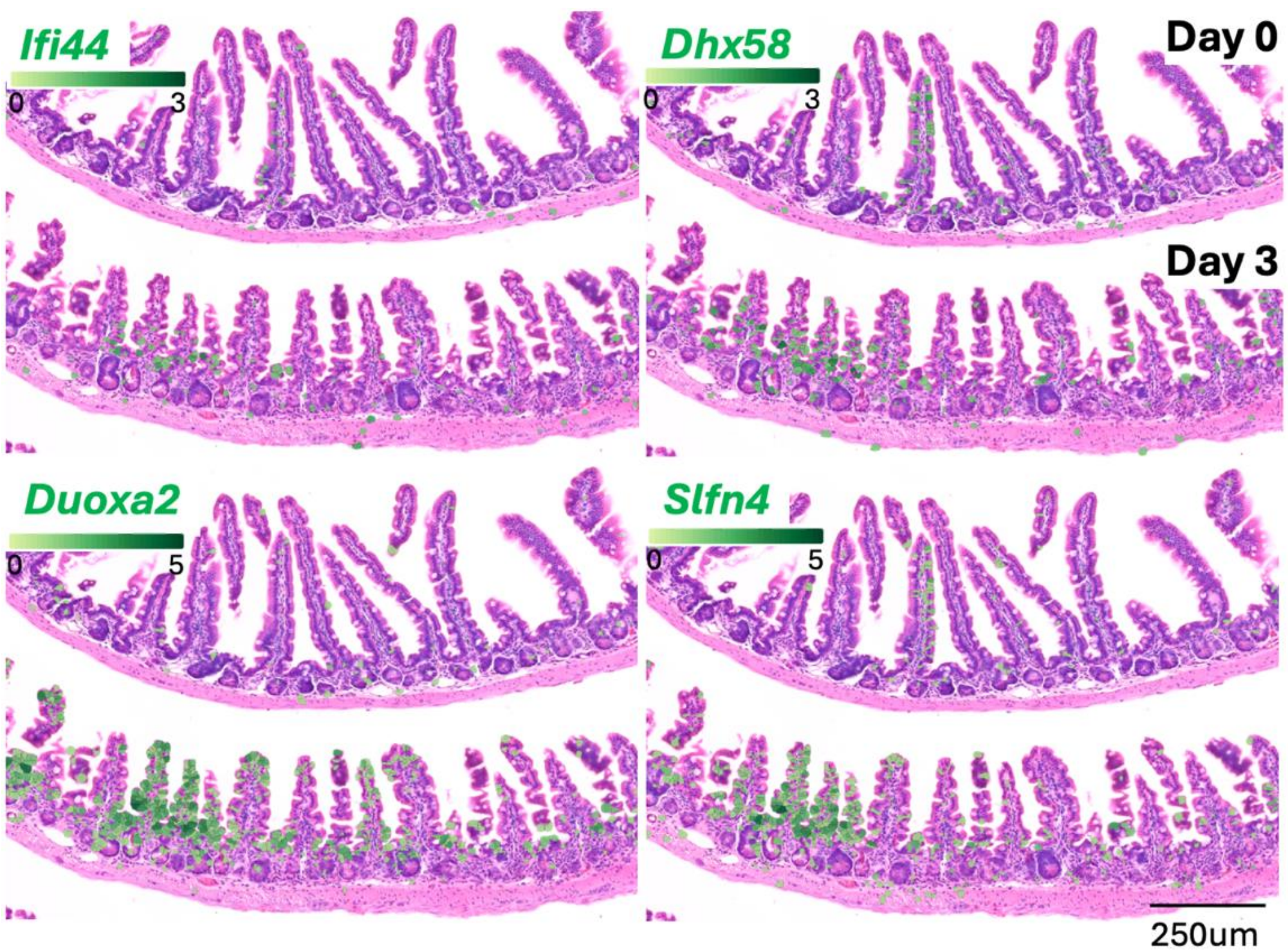

Figure R45 (Supplemental Figure 2g): Representative genes enriched in *Ifit2*$^{+}$ bins: *Ifi44, Dhx58, Duoxa2,* and *Slfn4*

We also looked more carefully at the spatial distribution of interferon signaling target genes, hoping to provide some potential insights on how the signal could be spreading between adjacent villi. We noted a large increase in *Isg15* bins within villus epithelial clusters at day 3 compared to day 0 (70% vs 18%), consistent with the idea that the villi are more responsive to this pathway. However, we see an even larger fold change in the number of cells at the villus-crypt junction domain expressing *Isg15* at day 3 (36% vs 5%), raising the possibility that the gain in signal at this villus junction region could be important in facilitating the spread of the IFN signal between adjacent villi. We provide new analysis, along with the gene list specific to the villus junction of adjacent IFN-active villi in a new Supplemental Figure 2. We thank the reviewers for finding this phenomenon exciting and hope our readers will be able to build upon this spatial analysis to provide new mechanisms of IFN-signaling spread in the intestine.

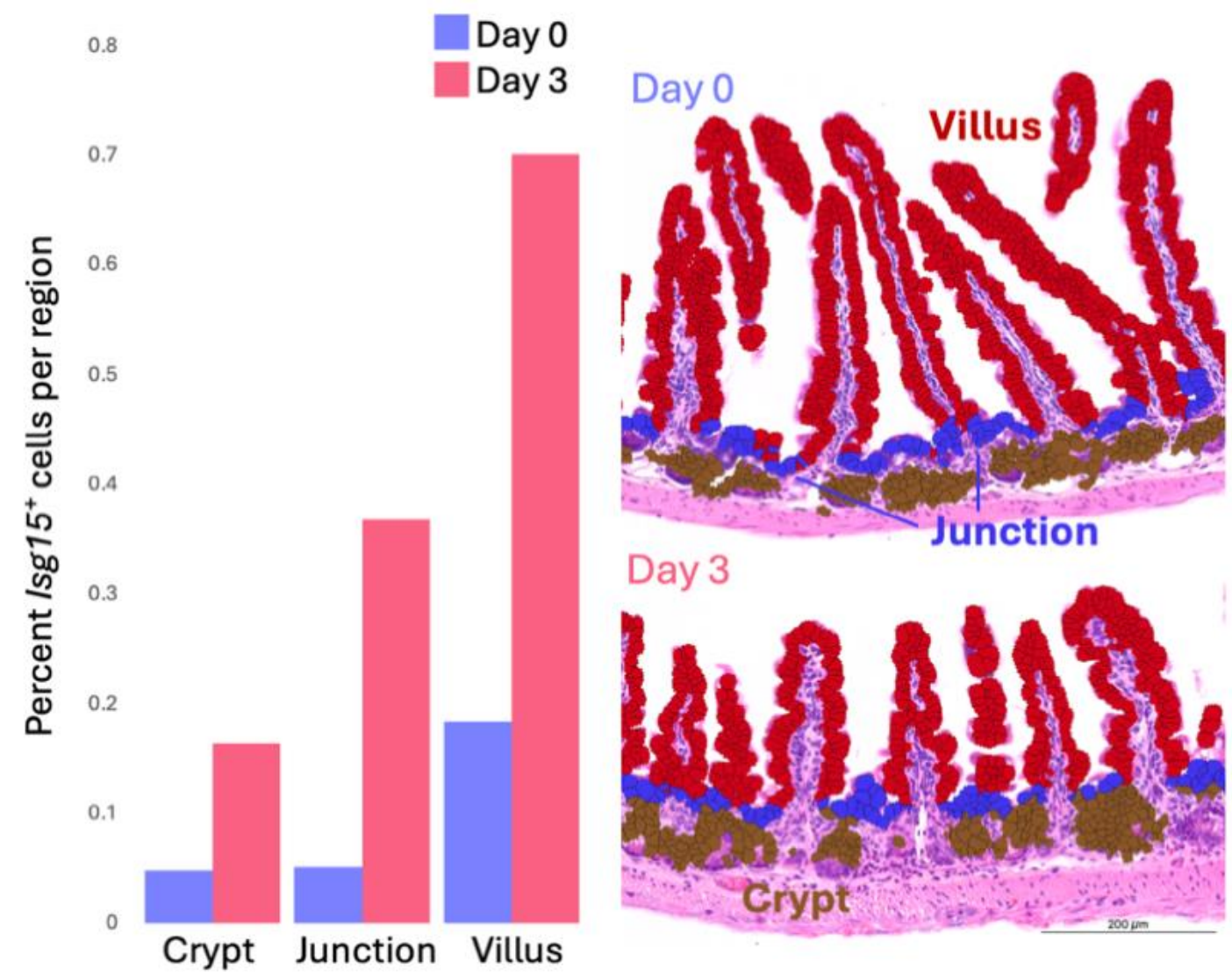

Figure R46 (Supplemental Figure 2d percent *Isg15*$^{+}$ cells within each of the indicated epithelial regions (villus epithelium, C-V junction and crypts) relative to all bins with that same region calculated for days 0 and 3. Results suggest, for example, on day 3, 70% of the villus epithelial bins are *Isg15*$^{+}$ (30% are *Isg15*$^{-}$), and 36% of the C-V junction epithelial bins are *Isg15*$^{+}$ (64% are *Isg15*$^{-}$).

We note that these findings represent spatially informed hypotheses generated from transcriptional co-enrichment within defined tissue compartments, and that dedicated experimental validation will be required to establish functional roles for these candidate genes during intestinal regeneration. We envision this dataset as a discovery resource for the field-enabling rapid spatial surveying of thousands of genes simultaneously within the native tissue context, allowing prioritization of candidates with spatially restricted or unexpected expression patterns before committing to downstream validation. We identify the junction-restricted, damage-induced *Ifit2*$^{+}$ population and its transcriptional signature as one such candidate and hope this dataset will similarly facilitate mechanistic exploration of other spatially defined programs during intestinal regeneration.

Minor Comments
1. The analysis code does not appear to be publicly available in the provided materials. While this may be addressed upon publication, explicit clarification is recommended.

We agree that this is an important suggestion. We have created a GitHub repository here https://github.com/tht38/intestinalspatialregen, with text updated to address it in the added "Code Availability" section.

2. Canonical jejunal markers should be shown to clearly confirm tissue identity.

This is a great suggestion. Here was show jejunal markers (*Gata4* and *Fabp1*) are expressed, while duodenal (*Pdx1*) and ileal (*Folh1*) markers are not expressed:

[FIGURE REDACTED]

Figure R47: day 0 timepoint expression of jejunal markers *Gata4* and *Fabp1* (top); duodenal marker *Pdx1* and ileal marker *Folh*1 (bottom). Markers from: Zwick et al. *Nat Cell Biol*, 2024.

3. In Supplementary Figure 1b, QC metrics are shown per chip rather than per sample. Given that multiple samples are processed on the same chip, per-sample QC metrics should be provided to exclude sample-specific biases.

We have added a new table showing this important information and pasted it here above (comment 1) for your reference. We thank the reviewer for this important suggestion.

4. The labels “Early” and “Late” are potentially misleading, as “Early” populations persist at later time points. Consider alternative terminology.

We thank the reviewer for this helpful suggestion. We agree that the terms "early" and "late" require clarification, as both populations persist and coexist across days 2–5 post-IR. We have revised the manuscript to more explicitly describe what "early-regenerative" and "late-regenerative" refer to: the "early" regenerative population first appears at day 2 post-IR and is enriched in the upper crypt, while the "late" regenerative population emerges beginning at day 3 and is enriched in the lower crypt. Both populations coexist at days 3-4 and are replaced by a distinct crypt cluster by day 5. We feel that retaining these terms with this added clarification best reflects the temporal emergence and spatial positioning of these two populations, and we have updated the text accordingly (Figure 3).

5. Cell segmentation is used in some panels (e.g., Figure. 5a–f) and 8-bin segmentation in others (Figure. 5g). A consistent approach would improve clarity.
We thank the reviewer for this important comment and have addressed it at multiple levels, including noting this caveat in the revised manuscript and providing a revised description of our analysis strategies in the methods section clarifying where cell segmentation was used and where

the 8-bin data was used. Specifically, all analysis shown in Figure 5 have been updated to use the cell segmentation (Figure 5a-f and k-l) or 2 µm bin data (Figure 5g-i and m)

Cell segmentation using the 2 µm bin framework constrains signal capture to areas approximating cell boundaries, reducing, though not eliminating, contributions from neighboring cells. The revised manuscript prioritizes 2µm bin analysis over the 8µm bin analysis featured in the previous version. Differential expression analysis applied a minimum average expression threshold per bin, excluding lowly expressed genes susceptible to noise or contamination from adjacent cells. Finally, we identify cells using clusters rather than single genes, such that cell type annotation is based on coherent co-expression of multiple established markers rather than any individual gene, making spurious annotation from a single contaminating cell type less likely. We believe these changes substantially improve the revised manuscript and thank the reviewer for raising this point.

We thank reviewer 3 and all the reviewers for their valuable contributions to improving this manuscript, and we hope it is now found suitable for publication as a resource for the research community.

References

1. PMC3916642; Fagerberg L, Hallström BM, Oksvold P, et al. Analysis of the human tissue-specific expression by genome-wide integration of transcriptomics and antibody-based proteomics. Mol Cell Proteomics. 2014;13(2):397-406. doi:10.1074/mcp.M113.035600
2. Van Winkle JA, Peterson ST, Kennedy EA, et al. Homeostatic interferon-lambda response to bacterial microbiota stimulates preemptive antiviral defense within discrete pockets of intestinal epithelium. Elife. 2022;11:e74072. Published 2022 Feb 9. doi:10.7554/eLife.74072
3. Constant, D. A., Van Winkle, J. A., Meyer, K. A., Madden, S. R., Reyes, B. R., & Nice, T. J. (2025). Homeostatic pockets of interferon lambda-stimulated gene production in the intestine are associated with localized exposure to bacterial microbiota. Gut microbes, 17(1), 2447830. https://doi.org/10.1080/19490976.2024.2447830
4. Moor AE, Harnik Y, Ben-Moshe S, et al. Spatial Reconstruction of Single Enterocytes Uncovers Broad Zonation along the Intestinal Villus Axis. Cell. 2018;175(4):1156-1167.e15. doi:10.1016/j.cell.2018.08.063Moor 2018
5. Ayyaz, A. *et al.* Single-cell transcriptomes of the regenerating intestine reveal a revival stem cell. *Nature* **569**, 121–125 (2019). https://doi.org/10.1038/s41586-019-1154-y
6. Yui, S. *et al.* YAP/TAZ-Dependent Reprogramming of Colonic Epithelium Links ECM Remodeling to Tissue Regeneration. *Cell Stem Cell* **22**, 35–49 e37 (2018). https://doi.org/10.1016/j.stem.2017.11.001
7. Chen, L. *et al.* TGFB1 induces fetal reprogramming and enhances intestinal regeneration. *Cell Stem Cell* **30**, 1520–1537.e1528 (2023). https://doi.org/10.1016/j.stem.2023.09.015

8. Mustata RC, Vasile G, Fernandez-Vallone V, et al. Identification of Lgr5-independent spheroid-generating progenitors of the mouse fetal intestinal epithelium. Cell Rep. 2013;5(2):421-432. doi:10.1016/j.celrep.2013.09.005
9. Ohara, T. E., Colonna, M., & Stappenbeck, T. S. (2022). Adaptive differentiation promotes intestinal villus recovery. Developmental cell, 57(2), 166–179.e6. https://doi.org/10.1016/j.devcel.2021.12.012
10. Gregorieff, A., Liu, Y., Inanlou, M. et al. Yap-dependent reprogramming of Lgr5+ stem cells drives intestinal regeneration and cancer. Nature 526, 715–718 (2015). https://doi.org/10.1038/nature15382
11. Kirino, S., Uefune, F., Miyake, K. et al. Fetal reversion from diverse lineages sustains the intestinal stem cell pool and confers stress resilience. Commun Biol 9, 255 (2026). https://doi.org/10.1038/s42003-026-09533-x
12. Van Winkle, J. A., Constant, D. A., Li, L., & Nice, T. J. (2020). Selective Interferon Responses of Intestinal Epithelial Cells Minimize Tumor Necrosis Factor Alpha Cytotoxicity. Journal of virology, 94(21), e00603-20.
13. McElrath, C., Espinosa, V., Lin, JD. et al. Critical role of interferons in gastrointestinal injury repair. Nat Commun 12, 2624 (2021). https://doi.org/10.1038/s41467-021-22928-0
14. Ruder B, Becker C. At the Forefront of the Mucosal Barrier: The Role of Macrophages in the Intestine. Cells. 2020;9(10):2162. Published 2020 Sep 24. doi:10.3390/cells9102162
15. Moraitis I, Taelman J, Arozamena B, et al. Mucosal Macrophages Govern Intestinal Regeneration in Response to Injury. Gastroenterology. 2025;169(1):119-135.e26. doi:10.1053/j.gastro.2025.01.252
16. Zwick, R.K., Kasparek, P., Palikuqi, B. et al. Epithelial zonation along the mouse and human small intestine defines five discrete metabolic domains. Nat Cell Biol 26, 250–262 (2024). https://doi.org/10.1038/s41556-023-01337-z
17. Munoz, J. *et al.* The Lgr5 intestinal stem cell signature: robust expression of proposed quiescent '+4' cell markers. *EMBO J* **31**, 3079–3091 (2012). https://doi.org/10.1038/emboj.2012.166
