# Supplementary material for: A spatial and temporal transcriptomic atlas of mouse intestinal regeneration in the jejunum
Source: Nat Commun. 2026 Sep 3;17:10484. doi: 10.1038/s41467-026-77292-8 (PMC13635233; doi:10.1038/s41467-026-77292-8)
Supplement: Supplementary file 3 [file 41467_2026_77292_MOESM1_ESM.pdf]

Supplementary Information for "**A Spatial and Temporal Transcriptomic Atlas of Mouse Intestinal Regeneration in the Jejunum**"

Rachel Ofer[1,2,3], Tyler Tran[1,2], Ping He[1-4], Xia Qiu[1,2], Xinyu Hu[1,2], Emmanuel Zachariah[3,5], Curtis Krier[3,5], Ankit Saxena[3,5], Lei Chen[6,7], Jiekun Yang[1,2,3], Michael P. Verzi [1,2,3,8,9*]

[1]Department of Genetics, Rutgers University, Piscataway, NJ, USA.
[2]Human Genetics Institute of New Jersey, Rutgers University, New Brunswick NJ, USA.
[3]Rutgers Cancer Institute of New Jersey, Rutgers University, New Brunswick NJ, USA.
[4]Department of Medicine, Rutgers University Robert Wood Johnson Medical School, New Brunswick, NJ, USA
[5]Center for Immune Monitoring and Flow Cytometry, Rutgers Cancer Institute of New Jersey, New Brunswick, NJ, USA
[6]School of Life Science and Technology, Key Laboratory of Developmental Genes and Human Disease, Southeast University, Nanjing 210031, China
[7]Institute of Microphysiological Systems, Southeast University, Nanjing 211189, China
[8]Rutgers Center for Lipid Research, New Jersey Institute for Food, Nutrition & Health, Rutgers University, New Brunswick, NJ, USA
[9]NIEHS Center for Environmental Exposures and Disease (CEED), Rutgers EOHSI, Piscataway, NJ, USA
[*]Corresponding author: Michael Verzi, 145 Bevier Rd. Piscataway, NJ 08854, mv347@hginj.rutgers.edu, 848-445-9578

Table of content

Supplementary Note 1

Spatial Data Guide for downloading and accessing spatial transcriptomic datasets.

Supplementary Figures 1- 6

Supplementary References

Spatial Data Guide

A quick guide to getting started with spatial transcriptomics datasets.

Introduction

- Spatial transcriptomics data were processed using 10x Genomics Space Ranger[1] (v3.1.3 and v4.0) and interactively visualized and explored using 10x Genomics Loupe Browser[2] (v8.0 and v9.0).
- LoupeR can be used to generate CLOUPE files from Seurat Objects.
- CLOUPE file output contains the gene expression matrices, spatial barcode information and image overlays. This file is designed to be opened using Loupe Browser.

Data & Code Availability

- All raw and processed data is deposited in GEO[3] under accession GSE303705[4] (https://www.ncbi.nlm.nih.gov/geo/query/acc.cgi?acc=GSE303705), including a CLOUPE files for each of the 4 Visium HD datasets.

- Code used is available at GitHub[5] (https://github.com/tht38/intestinalspatialregen) and Zenodo[6] https://doi.org/10.5281/zenodo.21348331.

Installation and setup

To view/access the spatial transcriptomics datasets, you will need to:

1. Download and install the latest version of Loupe browser from 10x Genomics.
2. Download the dataset (GSE303705[4]; CLOUPE file) from GEO database.
3. Open the CLOUPE file with Loupe browser- the dataset is ready to explore!

Available datasets

4 biological replicates are available as 4 Visium HD datasets. We recommend starting with Rep2_segmented_outputs_cloupe.cloupe or Rep2_square_008um_cloupe.cloupe.

Cloupe file name	Number of 2µm bins	Mean UMIs per 8µm bin	Genes detected	Post-IR Timepoints Included
Rep1_square_008um_cloupe.cloupe	6,644,192	144.1	19,035	D0, D1, D3, D5, D0
Rep2_square_008um_cloupe.cloupe	6,357,536	1189.4	19,037	D4, D2, D1, D5, D3, D0
Rep3_square_008um_cloupe.cloupe	6,374,112	713	19,029	D4, D2, D1, D0, D5, D3
Rep4_square_008um_cloupe.cloupe	6,308,240	1107.4	19,042	D2, D6, D2 D0, D4, D4

Cell segmentation output for rep2 is also available:

Cloupe file name	Number of cells	Mean reads per cell	Median genes per cell	Median UMIs per cell
Rep2_segmented_outputs_cloupe.cloupe	182,983	4,527.3	999	1502

<u>Example of 8x8um vs Cell Segmentation:</u>

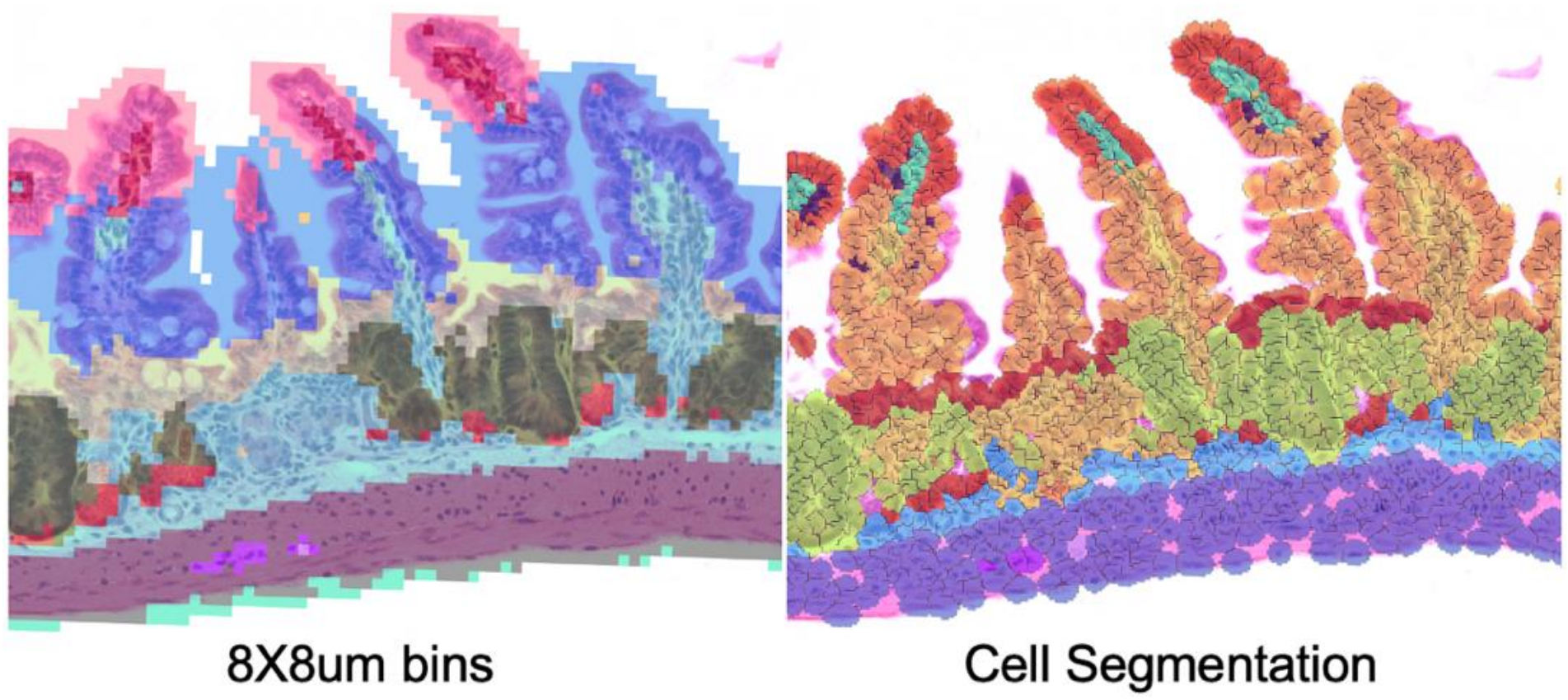

Treatment description per dataset with figures and timepoints post-irradiation

Each Visium HD capture area contains multiple mice, each representing a different post-irradiation timepoint. The images below show the timepoint of the tissues in each replicate.

Rep1 — capture area tissue order: Day 0, Day 1, Day 3, Day 5, Day 0.

Rep2 — capture area tissue order: Day 4, Day 2, Day 1, Day 5, Day 3, Day 0.

Rep3 — capture area tissue order: Day 4, Day 2, Day 1, Day 0, Day 5, Day 3.

Rep4 — capture area tissue order: Day 2, Day 6, Day 2, Day 0, Day 4, Day 4.

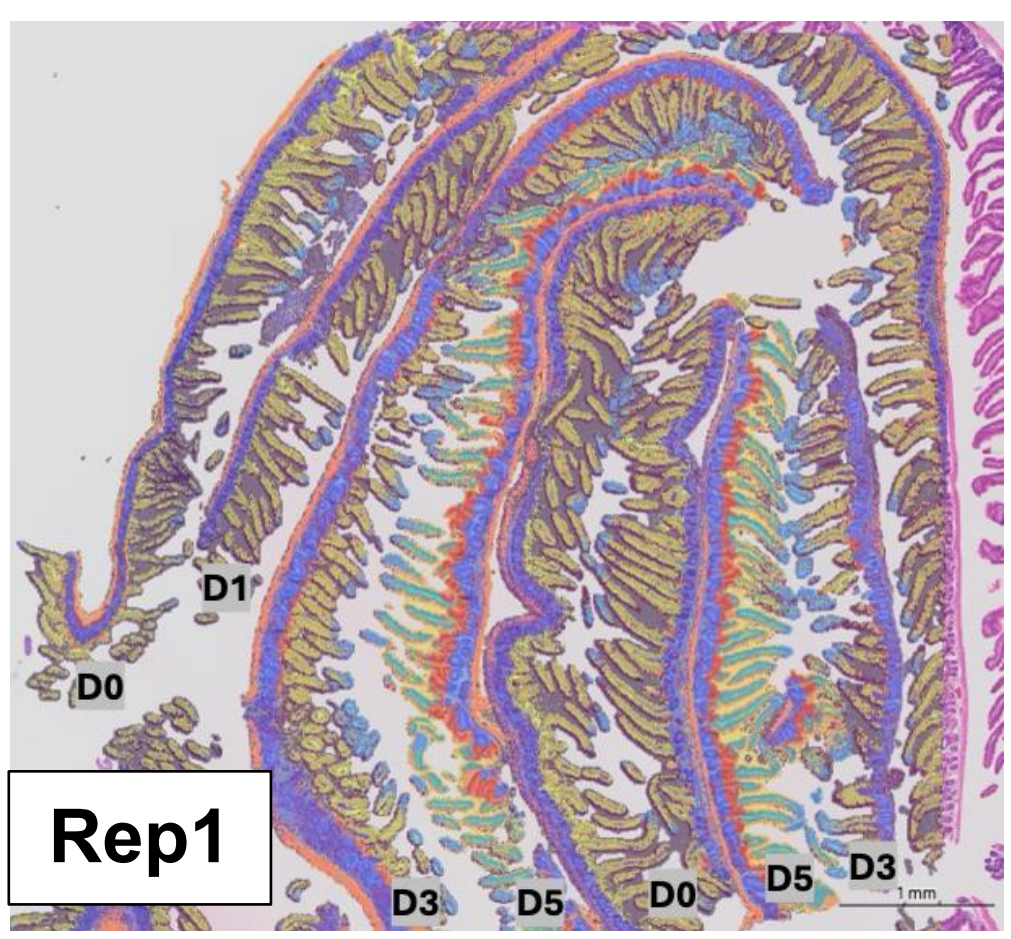

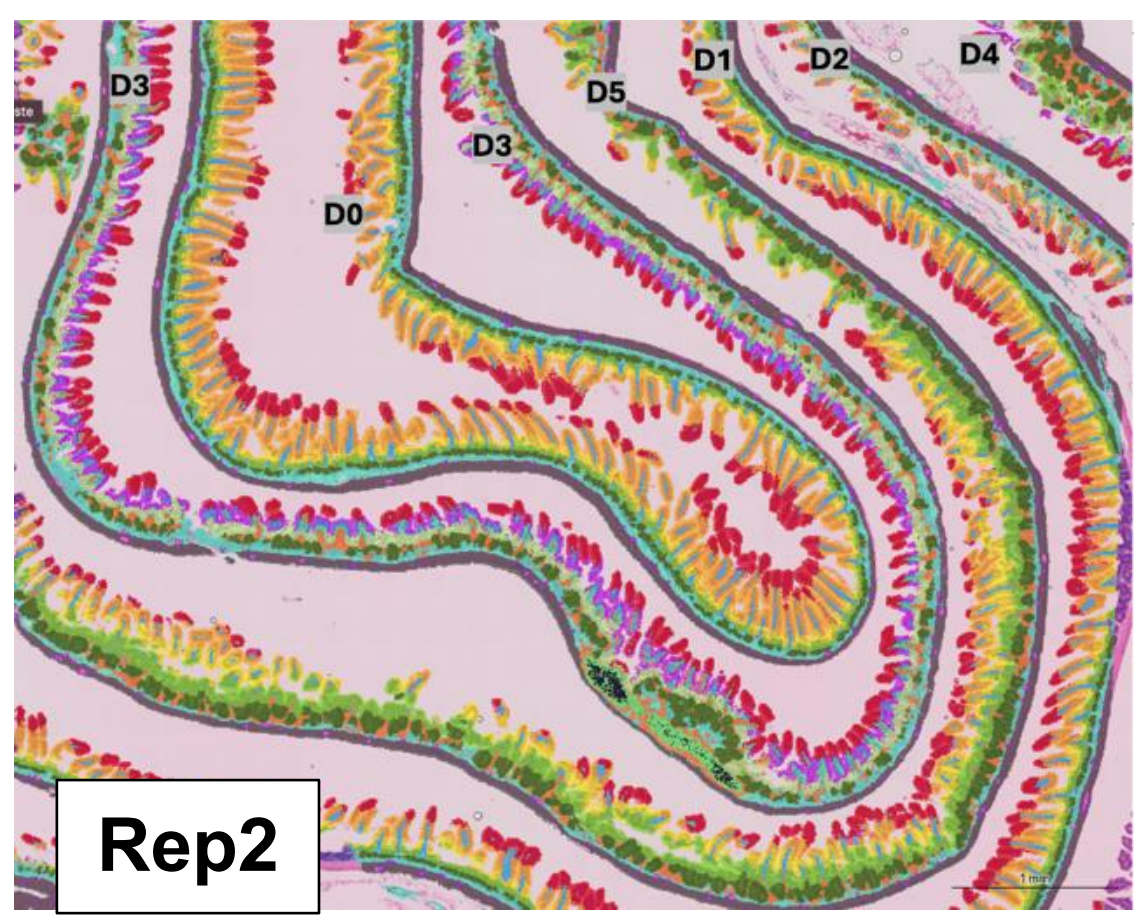

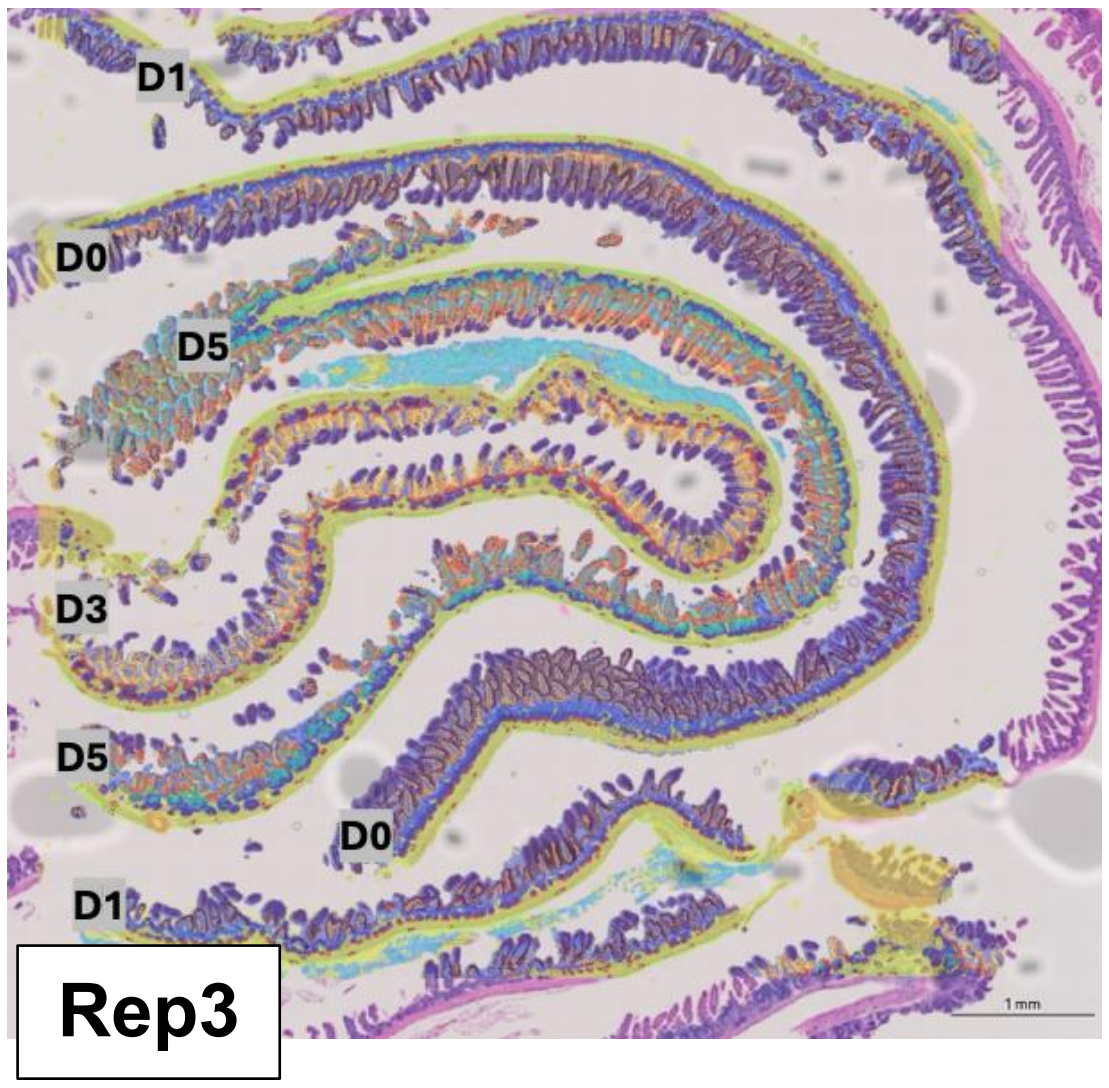

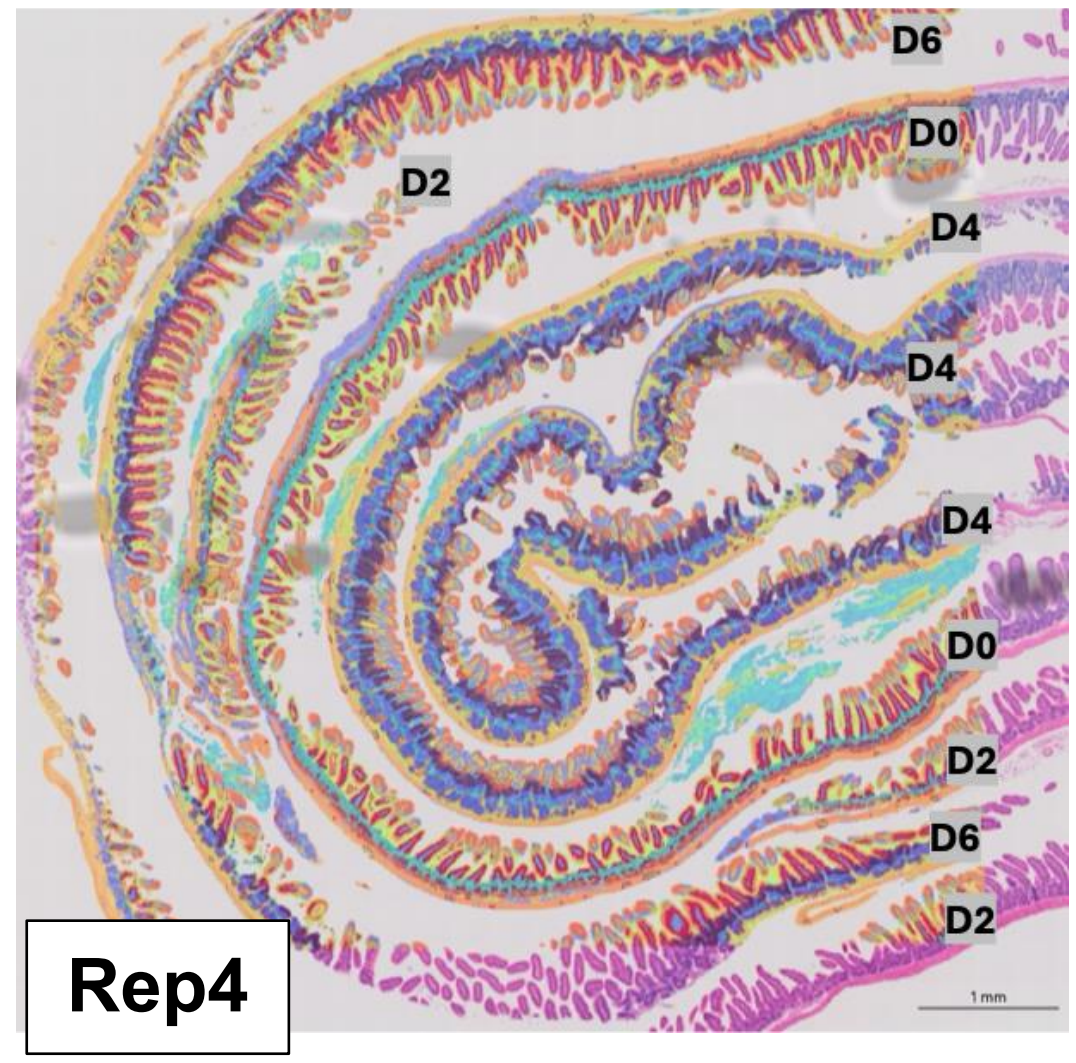

Loupe Browser Spatial Navigation Tutorial is a useful tool for further analysis strategies.

Source Data Fig. 1c contains a CSV file listing the barcodes assigned to each timepoint in the Visium dataset (Rep2). Uploading this file into Rep2 will regenerate the per-timepoint clusters shown in the figure.

Supplementary Figures

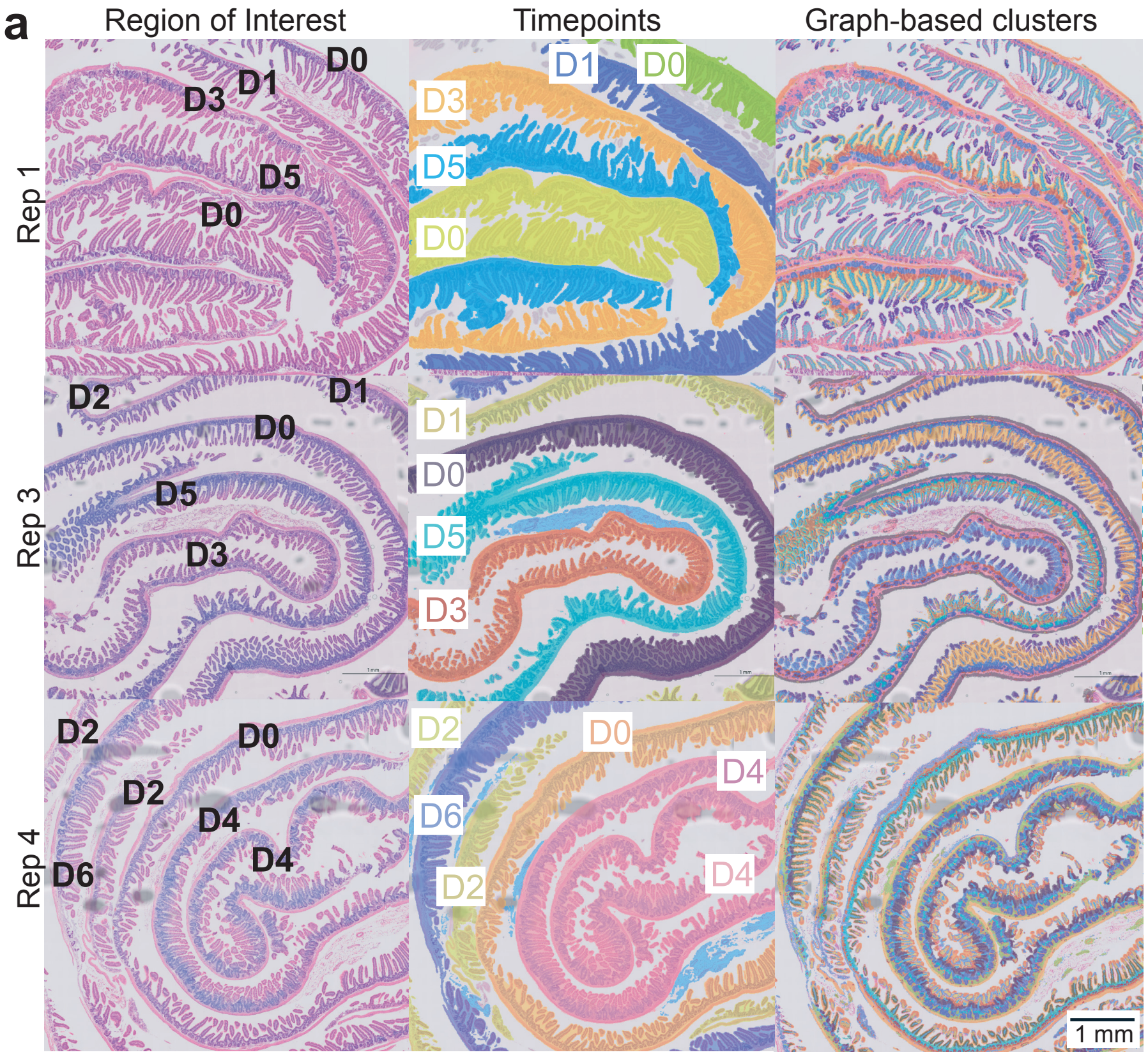

b Sequencing statistics and bin counts per Visium HD run

Timepoint	Unique reads	Mean reads per 8μm bins	Mean UMI per 8μm bins	2μm binned squares
Rep1	328,601,250	791.3	144.1	6,644,192
Rep2	1,126,204,763	2,834.3	1,189.4	6,357,536
Rep3	513,318,882	1,288.5	713	6,374,112
Rep4	831,962,821	1,920.2	1,107.4	6,308,240

c Number of 8-μm bins per timepoint, per replicate
*2 replicates combined

Timepoint	Day 0	Day 1	Day 2	Day 3	Day 4	Day 5	Day 6
Rep1	110,915*	65,580		89,965		72,168	
Rep2	82,366	70,405	26,653	84,043	7,276	85,695	
Rep3	97,387	82,219	24,661	68,233	8,436	99,601	
Rep4	91,960		68,616*		152,811*		98,240

d Number of segmented cells using Space Ranger analysis 2-μm bin-based cell segmented data

Timepoint	Day 0	Day 1	Day 2	Day 3	Day 4	Day 5
Rep2, Cell segmented	39,214	31,306	8,233	39,314	2,927	38,785

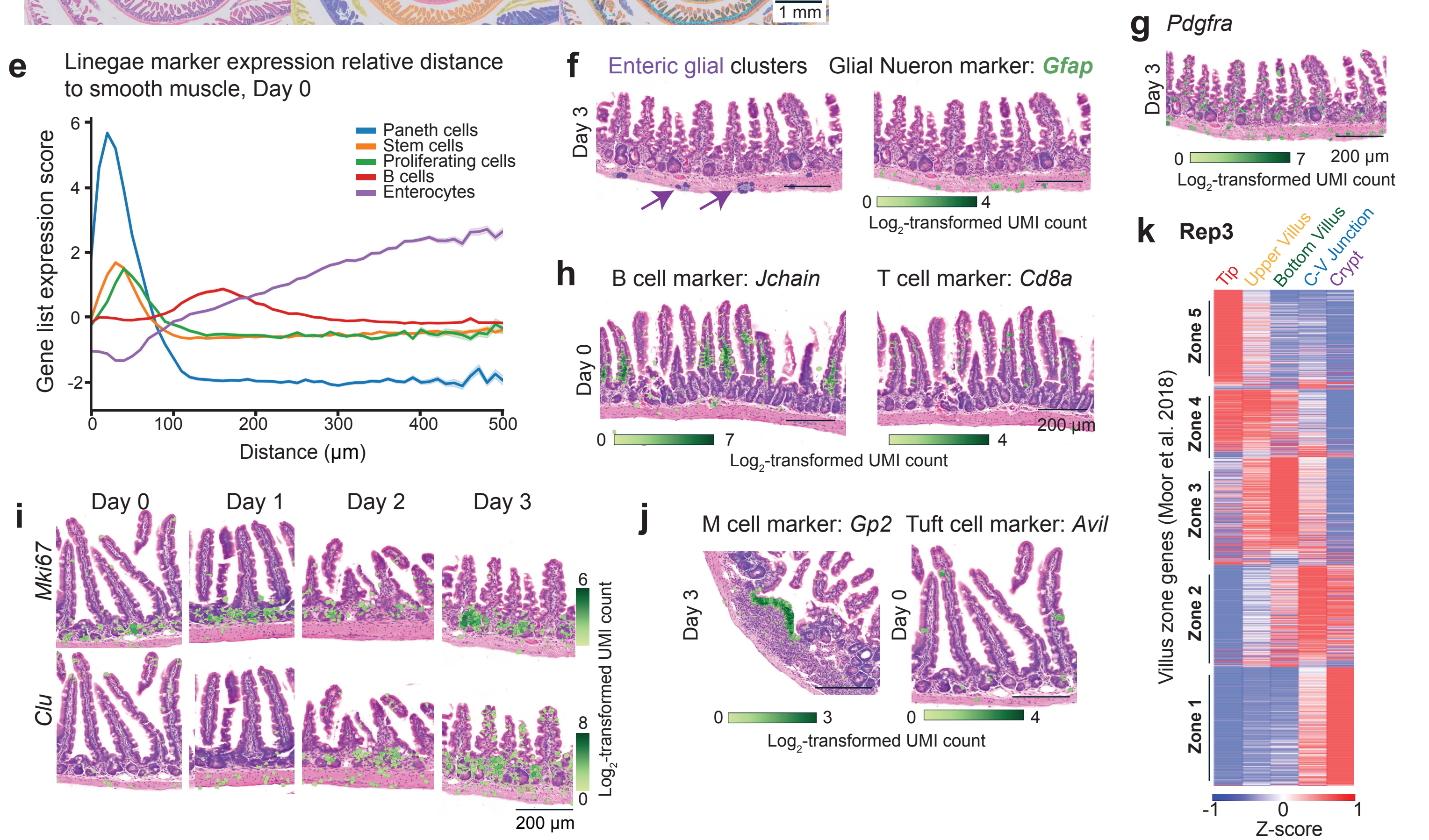

Supplementary Fig. 1. Data from multiple replicates exhibits consistent clustering and region-specific gene expression.

a, Spatial capture regions from replicates 1, 3, and 4; H&E, manually annotated timepoints, and unsupervised graph-based clustering of 8 µm bins. Rep1 *n* = 5; Rep3 *n* = 6; Rep4 *n* = 6 independent samples of the indicated timepoints post-IR.

b, Visium HD sequencing statistics and bin count for each replicate.

c, Number of 8 µm bins per timepoint, per replicate.

d, Number of segmented cells using 10x Genomics Space Ranger analysis of 2 µm bin data for rep2.

e, Relative expression of cell lineage marker gene panels across the crypt-villus axis, rep2. Gene lists are composed of Paneth cells (*Lyz1, Mmp7, Cd24a*); stem cells (*Olfm4, Lgr5*); proliferating cells (*Mki67, Pcna);* B cells (*Jchain, Cd19, Ms4a1*); and enterocytes (*Alpi, Krt20, Slc10a2*).

f, Graph-based spatial cluster annotated as enteric glial, and enteric glial marker *Gfap* (rep2). Representative image shown for illustration of the technology.

g–j, Spatiotemporal expression (log2-transformed UMI) of representative markers: **(g)** fibroblasts (*Pdgfra*), day 3; **(h)** B cell (*Jchain*) and T cell (*Cd8a*), day 0; **(i)** proliferation (*Mki67*) and regeneration marker (*Clu*), days 0–3; **(j)** M cell (*Gp2*), day 3; tuft cell (*Avil*), day 3.

k, Heatmap displaying Z-score of the five crypt-to-villus epithelial zone gene sets[7] 1,854 genes; tip, upper villus, bottom villus, crypt-villus junction, and crypt) across the five rep3 epithelial clusters. Z-scores were calculated from log2 fold change values from differential expression between the five rep3 epithelial clusters, computed in Loupe Browser[2] using a two-sided negative binomial-based test with Benjamini–Hochberg correction.

Scale bar: 1 mm **(a)**; 200 µm **(f–j)**

Source data are provided as a Source Data file for figure panel **(k)**

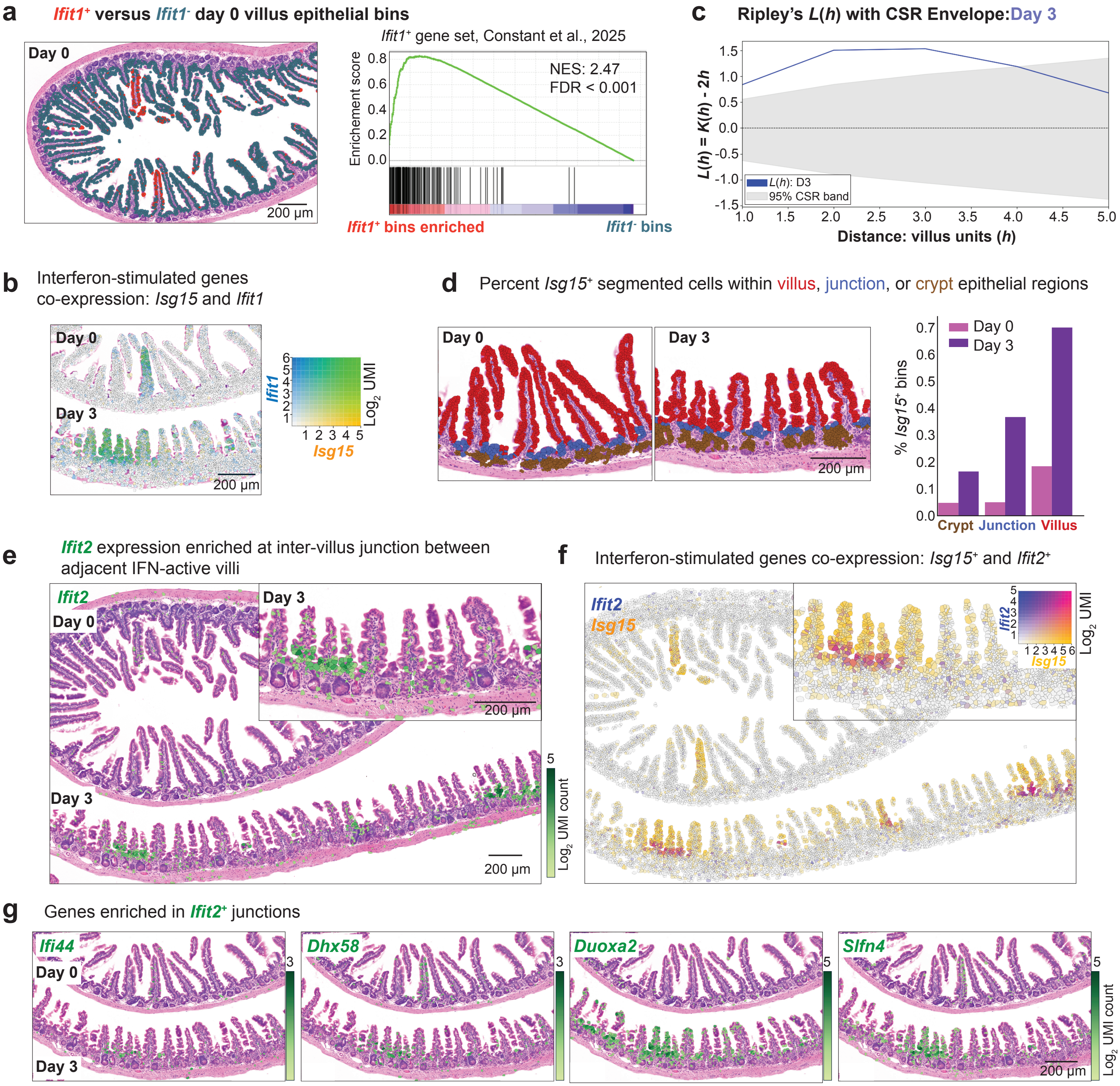

a
Ifit1+ versus Ifit1- day 0 villus epithelial bins
Day 0
200 µm
Ifit1+ gene set, Constant et al., 2025
Enrichement score
0.8
0.6
0.4
0.2
0.0
NES: 2.47
FDR < 0.001
Ifit1+ bins enriched
Ifit1- bins
c
Ripley's L(h) with CSR Envelope: Day 3
L(h) = K(h) - 2h
1.5
1.0
0.5
0.0
-0.5
-1.0
-1.5
L(h): D3
95% CSR band
1.0 1.5 2.0 2.5 3.0 3.5 4.0 4.5 5.0
Distance: villus units (h)
b
Interferon-stimulated genes co-expression: Isg15 and Ifit1
Day 0
Day 3
200 µm
Ifit1
Isg15
Log2 UMI
1 2 3 4 5
1 2 3 4 5 6
d
Percent Isg15+ segmented cells within villus, junction, or crypt epithelial regions
Day 0
Day 3
200 µm
% Isg15+ bins
0.7
0.6
0.5
0.4
0.3
0.2
0.1
0.0
Crypt
Junction
Villus
e
Ifit2 expression enriched at inter-villus junction between adjacent IFN-active villi
Ifit2
Day 0
Day 3
200 µm
5
Log2 UMI count
f
Interferon-stimulated genes co-expression: Isg15+ and Ifit2+
Ifit2
Isg15
Log2 UMI
1 2 3 4 5 6
1 2 3 4 5
g
Genes enriched in Ifit2+ junctions
Ifi44
Dhx58
Duoxa2
Slfn4
Day 0
Day 3
3
3
5
5
Log2 UMI count
200 µm

Supplementary Fig. 2. Interferon-stimulated genes exhibit spatially clustered expression in the intestinal epithelium that expands following irradiation.

a, Spatial transcriptomics profiling identifies discrete *Ifit1*$^{+}$ (red) and *Ifit1*$^{-}$ (blue) villus epithelial bins at day 0; rep2. Gene set enrichment analysis[8,9] (GSEA) shows enrichment of the *Ifit1*$^{+}$ gene set[10] in spatially defined *Ifit1*$^{+}$ bins (NES = 2.47, FDR < 0.001), demonstrating that spatial transcriptomic data recapitulate the transcriptional identity of *Ifit1*$^{+}$ epithelial cells.

b, Spatial co-expression of *Isg15*$^{+}$ and *Ifit1*$^{+}$ at day 0 reveals region-specific interferon-stimulated gene activation co-localizing on sparse villi, rep2.

c, Ripley's *L*-function (1D formulation) is used to assess spatial clustering of *Isg15*$^{+}$ villi, revealing significant clustering at day 3 post-IR at distances corresponding to multiple surrounding villi. Significance is assessed against a 95% simulation envelope (gray shaded area) derived from 1,000 Monte Carlo permutations assuming a random point pattern with the same number of *Isg15*$^{+}$ villi as observed.

d, Percentage of *Isg15*$^{+}$ bins within crypt, crypt-villus junction, and villus epithelial regions at day 0 and day 3 (rep2), relative to all bins within each respective region.

e–f, *Ifit2* expression at crypt-villus junction regions at day 0 and day 3 **(e)**, and co-expression of *Ifit2* and *Isg15* **(f)**; *Ifit2* expression is enriched specifically at the inter-villus junction between adjacent IFN-active villi at day 3.

g, Representative genes enriched in *Ifit2*$^{+}$ junction bins: *Ifi44*, *Dhx58*, *Duoxa2* and *Slfn4*. Differential expression between *Ifit2*$^{+}$ and *Ifit2*$^{-}$ day-3 crypt–villus junction bins, computed in Loupe Browser using a two-sided negative binomial-based test with Benjamini–Hochberg correction, identified 23 enriched genes (adj. $P < 0.05$, MNA > 0.05, $\log_2$FC > 1); the full gene list is provided in Supplementary Data 3.

Full gene list and GO results for **(a,e–g)** in Supplementary Data 3.

Scale bar: 200 µm

Source data are provided as a Source Data file for figure panel **(a)**

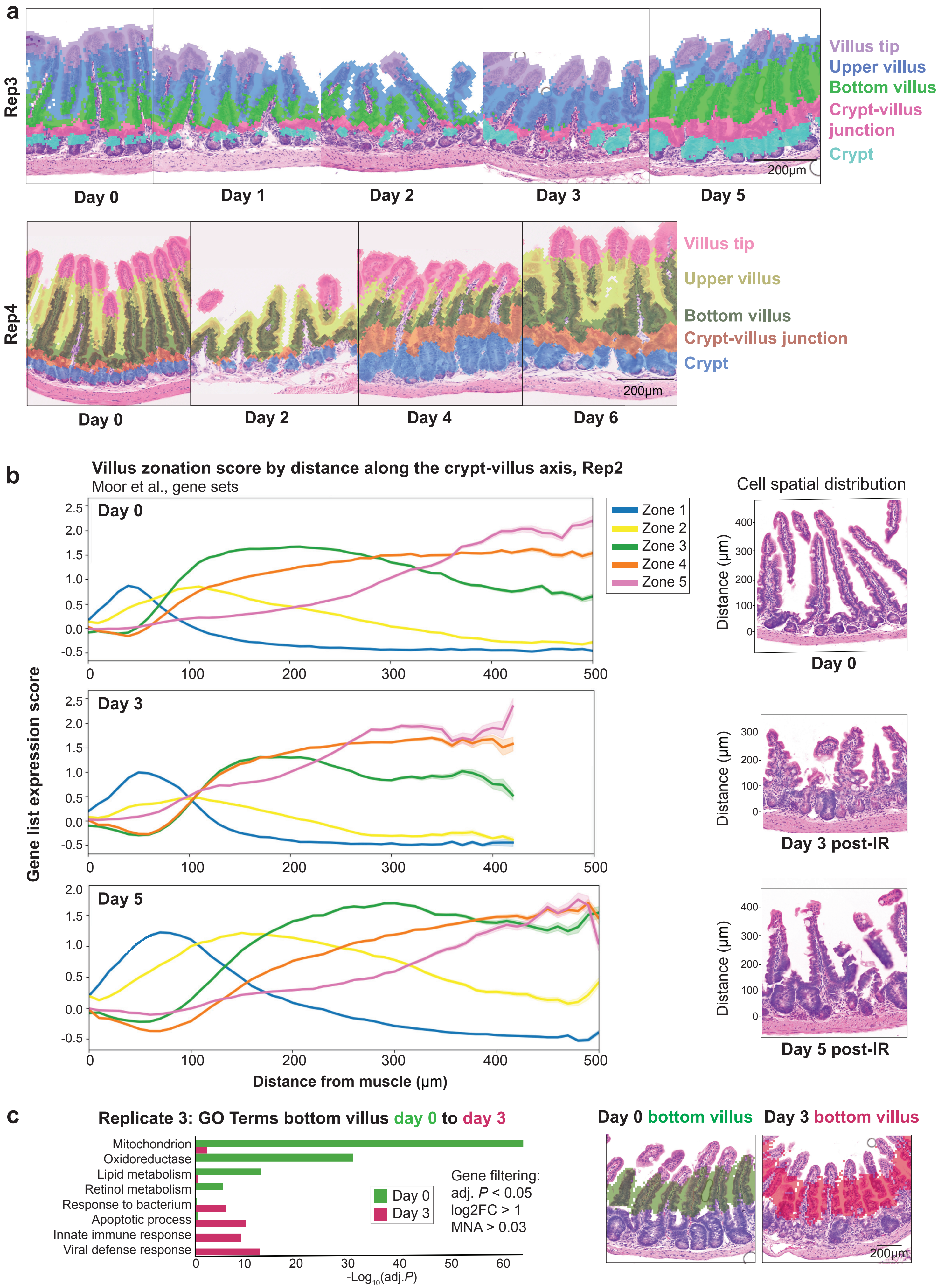

a
Rep3
Villus tip
Upper villus
Bottom villus
Crypt-villus junction
Crypt
200µm
Day 0
Day 1
Day 2
Day 3
Day 5
Rep4
Villus tip
Upper villus
Bottom villus
Crypt-villus junction
Crypt
200µm
Day 0
Day 2
Day 4
Day 6
b
Villus zonation score by distance along the crypt-villus axis, Rep2
Moor et al., gene sets
Zone 1
Zone 2
Zone 3
Zone 4
Zone 5
Day 0
Day 3
Day 5
Gene list expression score
Distance from muscle (µm)
Cell spatial distribution
Distance (µm)
Day 0
Day 3 post-IR
Day 5 post-IR
c
Replicate 3: GO Terms bottom villus day 0 to day 3
Mitochondrion
Oxidoreductase
Lipid metabolism
Retinol metabolism
Response to bacterium
Apoptotic process
Innate immune response
Viral defense response
Day 0
Day 3
Gene filtering:
adj. P < 0.05
log2FC > 1
MNA > 0.03
-Log10(adj.P)
Day 0 bottom villus
Day 3 bottom villus
200µm

Supplementary Fig. 3. Alterations in villus patterning appear during regeneration across biological replicates.

a, Unsupervised clustering identifies five transcriptionally distinct epithelial clusters (villus tip, upper villus, bottom villus, crypt-villus junction, and crypt), revealing a shift in zonation patterns that is consistent across rep3 and rep4 (see Supplementary Fig. 1a for *n* per replicate).

b, Gene list expression score of villus epithelial zonation genes[7] (Moor et al., 2018) along the crypt-villus axis on days 0, 3, and 5 post-IR, rep2. Shown as the average of 10 μm windows along the crypt-villus axis as measured by distance from the smooth muscle cluster, with SEM shaded.

c, Differential gene expression between the bottom villus cluster of day 0 and the bottom villus cluster of day 3 (rep3), computed in Loupe Browser using a two-sided negative binomial-based test with Benjamini–Hochberg correction (adj. $P < 0.05$, log2FC > 1, MNA > 0.03); GO terms[11,12] analysis of data from rep3 confirms the reproducibility of the findings shown in Fig. 2h. Full gene list and GO results in Supplementary Data 6.

Scale bar: 200 μm

Source data are provided as a Source Data file for figure panel **(c)**.

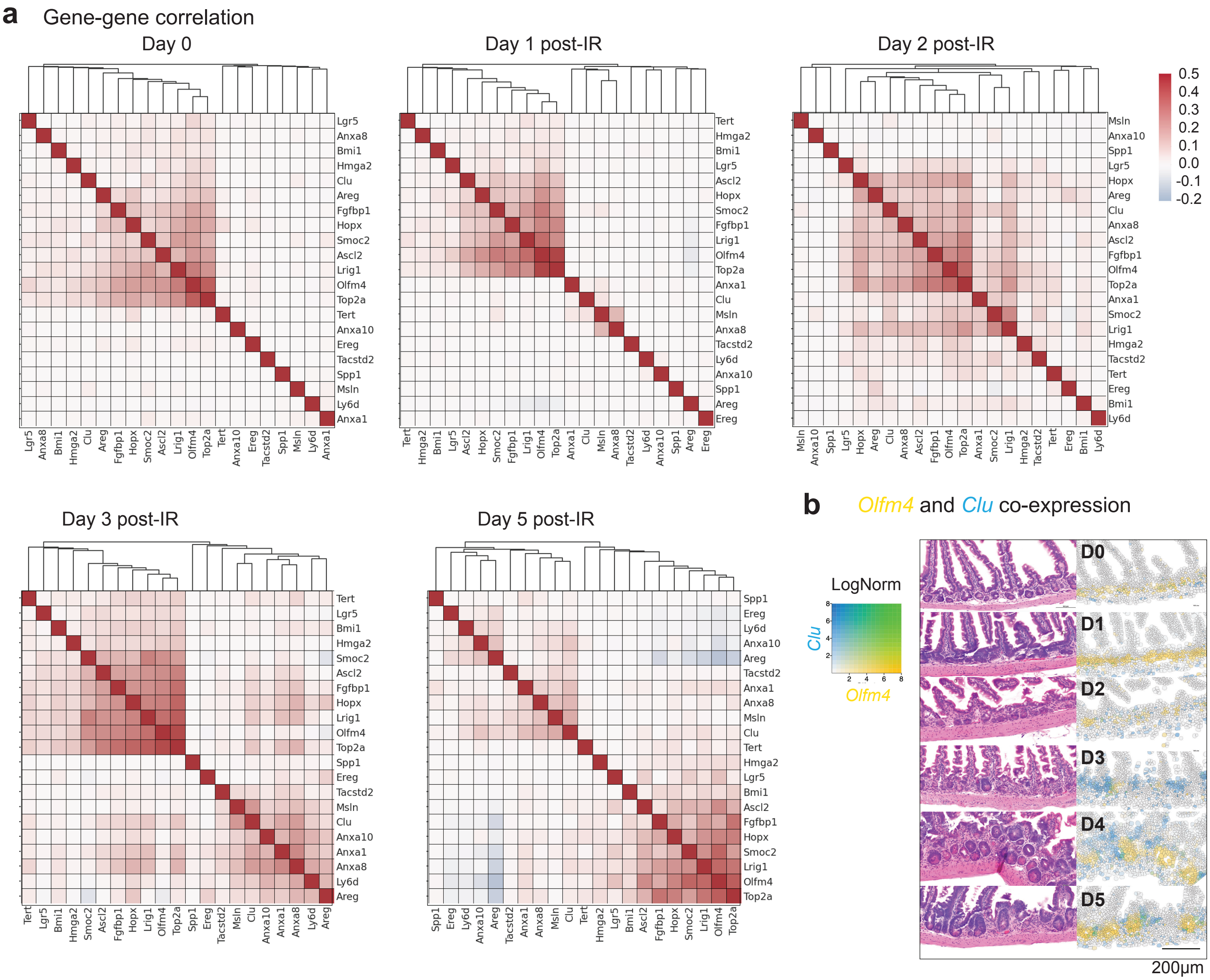
a
Gene-gene correlation
Day 0
Lgr5
Anxa8
Bmi1
Hmga2
Clu
Areg
Fgfbp1
Hopx
Smoc2
Ascl2
Lrig1
Olfm4
Top2a
Tert
Anxa10
Ereg
Tacstd2
Spp1
Msln
Ly6d
Anxa1
Day 1 post-IR
Tert
Hmga2
Bmi1
Lgr5
Ascl2
Hopx
Smoc2
Fgfbp1
Lrig1
Olfm4
Top2a
Anxa1
Clu
Msln
Anxa8
Tacstd2
Ly6d
Anxa10
Spp1
Areg
Ereg
Day 2 post-IR
Msln
Anxa10
Spp1
Lgr5
Hopx
Areg
Clu
Anxa8
Ascl2
Fgfbp1
Olfm4
Top2a
Anxa1
Smoc2
Lrig1
Hmga2
Tacstd2
Tert
Ereg
Bmi1
Ly6d
0.5
0.4
0.3
0.2
0.1
0.0
-0.1
-0.2
Day 3 post-IR
Tert
Lgr5
Bmi1
Hmga2
Smoc2
Ascl2
Fgfbp1
Hopx
Lrig1
Olfm4
Top2a
Spp1
Ereg
Tacstd2
Msln
Clu
Anxa10
Anxa1
Anxa8
Ly6d
Areg
Day 5 post-IR
Spp1
Ereg
Ly6d
Anxa10
Areg
Tacstd2
Anxa1
Anxa8
Msln
Clu
Tert
Hmga2
Lgr5
Bmi1
Ascl2
Fgfbp1
Hopx
Smoc2
Lrig1
Olfm4
Top2a
b
Olfm4 and Clu co-expression
LogNorm
Clu
Olfm4
D0
D1
D2
D3
D4
D5
200µm

Supplementary Fig. 4. Pairwise co-clustering of regenerative and homeostatic stem cell marker genes suggests distinct cell states in the regenerating crypts.

a, Gene-gene co-expression correlation analysis of genes previously associated with homeostatic and regenerative stem cells reveals that co-expression relationships changed from homeostasis (day 0) to regeneration (days 1–5 post-IR), rep2.

b, Co-expression of *Olfm4* and *Clu* as representative markers of these homeostatic and regenerative cell populations, respectively, rep2.

Scale bar: 100 μm

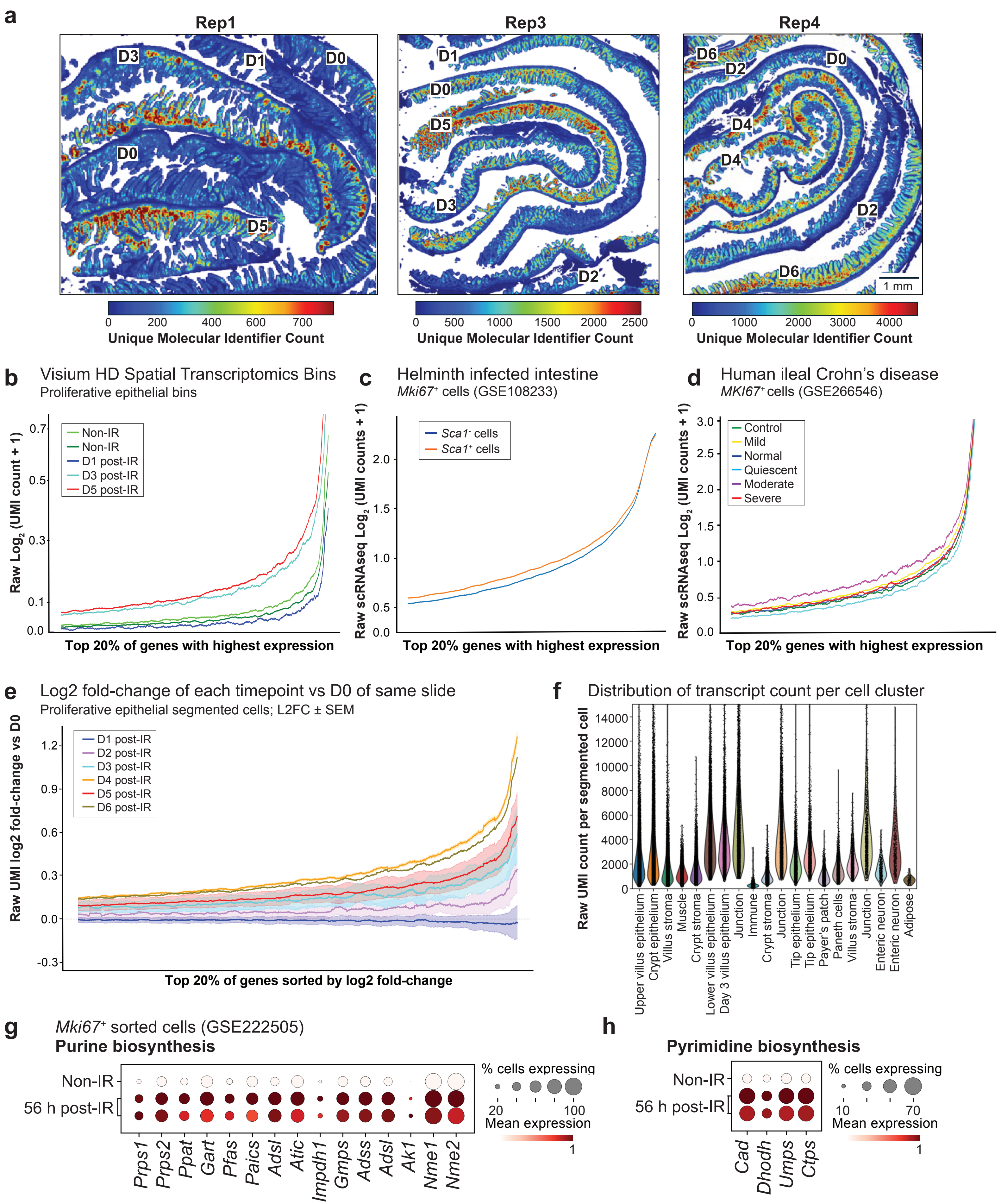

a
Rep1
Rep3
Rep4
D3
D1
D0
D0
D5
0
200
400
600
700
Unique Molecular Identifier Count
D1
D0
D5
D3
D2
0
500
1000
1500
2000
2500
Unique Molecular Identifier Count
D6
D2
D0
D4
D4
D2
D6
1 mm
0
1000
2000
3000
4000
Unique Molecular Identifier Count
b
Visium HD Spatial Transcriptomics Bins
Proliferative epithelial bins
Non-IR
Non-IR
D1 post-IR
D3 post-IR
D5 post-IR
Raw Log2 (UMI count + 1)
Top 20% of genes with highest expression
c
Helminth infected intestine
Mki67+ cells (GSE108233)
Sca1- cells
Sca1+ cells
Raw scRNAseq Log2 (UMI counts + 1)
Top 20% genes with highest expression
d
Human ileal Crohn's disease
MKI67+ cells (GSE266546)
Control
Mild
Normal
Quiescent
Moderate
Severe
Raw scRNAseq Log2 (UMI counts + 1)
Top 20% genes with highest expression
e
Log2 fold-change of each timepoint vs D0 of same slide
Proliferative epithelial segmented cells; L2FC ± SEM
D1 post-IR
D2 post-IR
D3 post-IR
D4 post-IR
D5 post-IR
D6 post-IR
Raw UMI log2 fold-change vs D0
Top 20% of genes sorted by log2 fold-change
f
Distribution of transcript count per cell cluster
Raw UMI count per segmented cell
Upper villus epithelium
Crypt epithelium
Villus stroma
Muscle
Crypt stroma
Lower villus epithelium
Day 3 villus epithelium
Junction
Immune
Crypt stroma
Junction
Tip epithelium
Tip epithelium
Payer's patch
Paneth cells
Villus stroma
Junction
Enteric neuron
Enteric neuron
Adipose
g
Mki67+ sorted cells (GSE222505)
Purine biosynthesis
Non-IR
56 h post-IR
Prps1
Prps2
Ppat
Gart
Pfas
Paics
Adsl
Atic
Impdh1
Gmps
Adss
Adsl
Ak1
Nme1
Nme2
% cells expressing
20
100
Mean expression
1
h
Pyrimidine biosynthesis
Non-IR
56 h post-IR
Cad
Dhodh
Umps
Ctps
% cells expressing
10
70
Mean expression
1

Supplementary Fig. 5. Hypertranscription in regenerating intestinal tissues across multiple replicates.

a, Heatmaps of total raw UMI counts per barcode in spatial replicates 1, 3, and 4 show global elevated transcriptional upregulation at regenerative timepoints post-IR.

b–d, Global amplification of the transcriptome in regenerating tissues. (**b**) Mean log2-transformed raw UMI counts of the top 20% most highly expressed genes, plotted across timepoints in rep1 spatial data; bins are limited to the crypt cluster defined by unsupervised graph-based spatial clustering (*n* stated on panel). The same analysis was applied to (**c**) *Mki67*$^{+}$ cells subset from sorted *Sca-1*$^{+}$ and *Sca-1*$^{-}$ epithelial populations after helminth infection (Nusse et al., 2018; GSE108233[13]; *n* = 70,000 cells each) and (**d**) human Crohn's disease ileal data (Li et al., 2024; GSE266546[14]; *n* = 82 patients). In each case, the regenerative condition shows higher raw RNA counts per cell or per spatial bin, indicative of hypertranscription.

e, Log2FC of each gene's raw expression per bin for each timepoint relative to day 0 of the same experiment, averaged across all 4 biological replicates and all timepoints (*n* = 23 mice), with SEM shading. Cells were selected by co-expression of *Mki67, Epcam* and *Vil1* to identify proliferative epithelial cells. The x-axis shows individual genes sorted by average log2 fold change across all replicates and timepoints.

f, Violin plot of raw UMI counts per segmented cell across intestinal cell populations identified by Visium HD spatial transcriptomics clustering, rep2. Epithelial populations display substantially higher UMI counts per cell than non-epithelial populations.

g–h, Genes with nucleotide anabolic activity are elevated at day 3 post-IR. Normalized mean-expression dot plots show increased de novo purine (**g**) and pyrimidine (**h**) biosynthesis gene expression in FACS-sorted *Mki67*$^{+}$ epithelial cells at 56 h post-IR (*n* = 2 mice) relative to non-IR controls (*n* = 1 mouse); scRNA-seq (Chen et al., 2023; GSE222505[15]).

Scale bar: 1 mm **(a)**

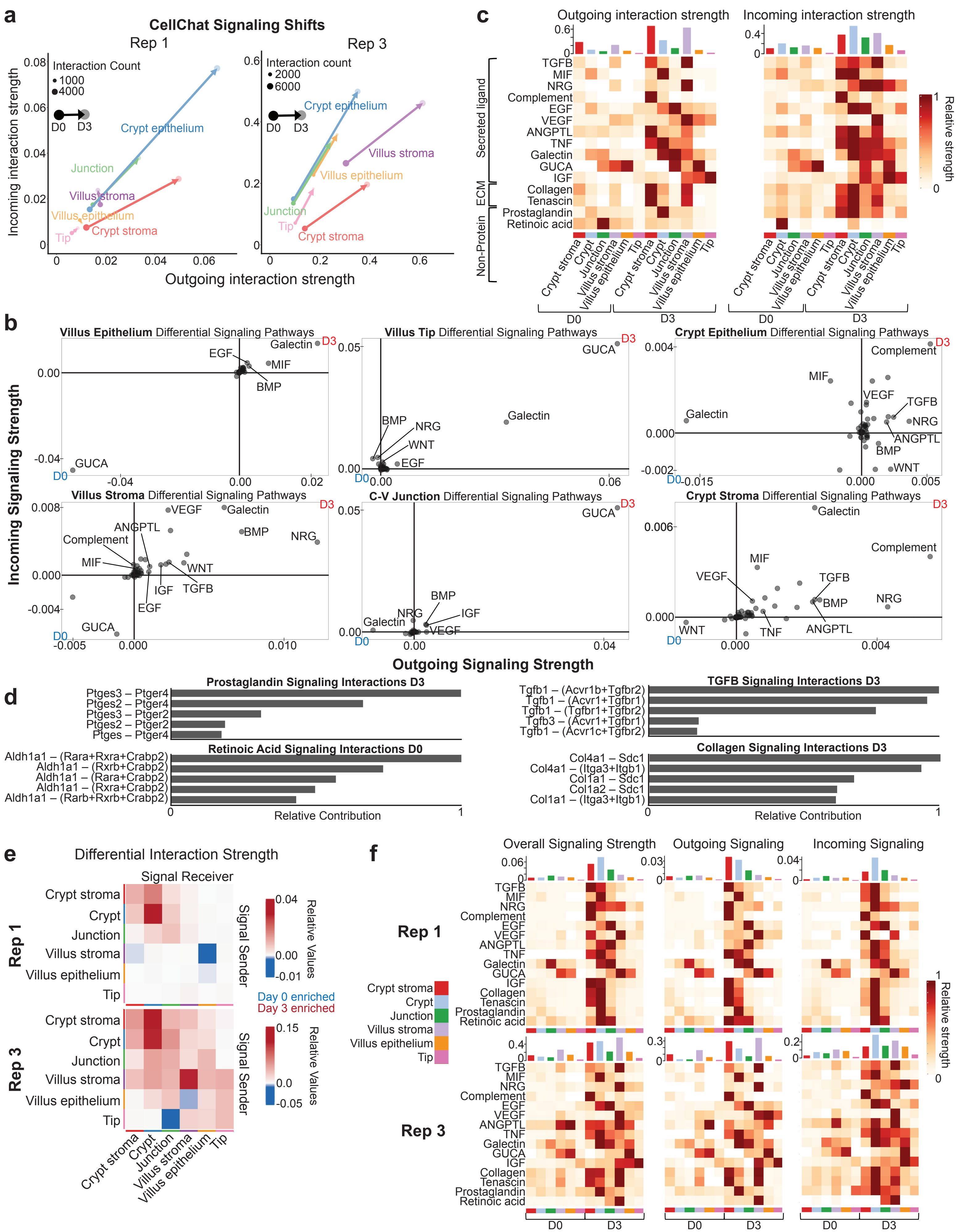
a
CellChat Signaling Shifts
Rep 1
Rep 3
Incoming interaction strength
Outgoing interaction strength
Interaction Count
1000
4000
D0 D3
Crypt epithelium
Junction
Villus stroma
Villus epithelium
Tip
Crypt stroma
Interaction count
2000
6000
c
Outgoing interaction strength
Incoming interaction strength
Secreted ligand
ECM
Non-Protein
TGFB
MIF
NRG
Complement
EGF
VEGF
ANGPTL
TNF
Galectin
GUCA
IGF
Collagen
Tenascin
Prostaglandin
Retinoic acid
Relative strength
Crypt stroma
Crypt
Junction
Villus stroma
Villus epithelium
Tip
D0
D3
b
Villus Epithelium Differential Signaling Pathways
Villus Tip Differential Signaling Pathways
Crypt Epithelium Differential Signaling Pathways
Villus Stroma Differential Signaling Pathways
C-V Junction Differential Signaling Pathways
Crypt Stroma Differential Signaling Pathways
Incoming Signaling Strength
Outgoing Signaling Strength
d
Prostaglandin Signaling Interactions D3
Ptges3 – Ptger4
Ptges2 – Ptger4
Ptges3 – Ptger2
Ptges2 – Ptger2
Ptges – Ptger4
Retinoic Acid Signaling Interactions D0
Aldh1a1 – (Rara+Rxra+Crabp2)
Aldh1a1 – (Rxrb+Crabp2)
Aldh1a1 – (Rara+Crabp2)
Aldh1a1 – (Rxra+Crabp2)
Aldh1a1 – (Rarb+Rxrb+Crabp2)
TGFB Signaling Interactions D3
Tgfb1 – (Acvr1b+Tgfbr2)
Tgfb1 – (Acvr1+Tgfbr1)
Tgfb1 – (Tgfbr1+Tgfbr2)
Tgfb3 – (Acvr1+Tgfbr1)
Tgfb1 – (Acvr1c+Tgfbr2)
Collagen Signaling Interactions D3
Col4a1 – Sdc1
Col4a1 – (Itga3+Itgb1)
Col1a1 – Sdc1
Col1a2 – Sdc1
Col1a1 – (Itga3+Itgb1)
Relative Contribution
e
Differential Interaction Strength
Signal Receiver
Signal Sender
Rep 1
Rep 3
Relative Values
Day 0 enriched
Day 3 enriched
f
Overall Signaling Strength
Outgoing Signaling
Incoming Signaling
Rep 1
Rep 3

Supplementary Fig. 6. Replicate analysis confirms regional stromal signaling changes during intestinal regeneration.

CellChat[16]-inferred signaling analyses in this figure were performed in replicates 1 (*n* = 5 mice) and 3 (*n* = 6 mice), in addition to replicate 2 (*n* = 6 mice; Fig. 5), for a total of 3 biological replicates demonstrating reproducibility of stromal signaling changes. As in Fig. 5, all values represent relative predicted interaction scores from CellChatDB; no statistical test was applied.

a, Differences in outgoing and incoming signaling strength between day 0 and day 3 post-IR for each stromal region, based on CellChat-inferred signaling interactions in replicates 1 and 3.

b, Outgoing and incoming signaling strength by region at day 0 and day 3 post-IR, highlighting pathways previously implicated in epithelial regeneration, including TGFβ, complement, and MIF. Results indicate region-specific changes in secretion and reception roles in replicate 2.

c, Differential pathway-level signaling strength between day 0 and day 3 for each stromal domain, based on CellChatDB's secreted ligand signaling database. Signaling pathways previously associated with regeneration are annotated.

d, Relative contribution of the top 5 specific genes within the predicted cell-cell signaling pathways of prostaglandin, TGFβ, collagen, and retinoic acid signaling, scaled to the strongest interaction per signaling pathway; values represent relative pathway contribution scores from CellChatDB, no statistical test applied.

e, Comparison of CellChat-inferred interaction networks at day 0 (blue) and day 3 (red) for replicates 1 and 3. A consistent regeneration-associated increase in stromal signaling activity is observed across replicates.

f, Outgoing, incoming, and total signaling activity by region for select regeneration-relevant pathways across replicates 1 and 3.

References

1 Space Ranger Analysis Pipelines v. 3.0 (10x Genomics, https://www.10xgenomics.com/support/software/space-ranger/latest, 2024).

2 Loupe Browser v. 8.0 (10x Genomics, https://www.10xgenomics.com/support/software/loupe-browser/latest 2024).

3 Edgar, R., Domrachev, M. & Lash, A. E. Gene Expression Omnibus: NCBI gene expression and hybridization array data repository. *Nucleic Acids Res* **30**, 207–210 (2002). https://doi.org/10.1093/nar/30.1.207

4 Ofer R *et al.* in *A Spatial and Temporal Transcriptomic Atlas of Mouse Intestinal Regeneration in the Jejunum* (https://www.ncbi.nlm.nih.gov/geo/query/acc.cgi?acc=GSE303705, Gene Expression Omnibus GSE303705, 2025).

5 Ofer, R., Tran, T. & Verzi, M. P. in *A Spatial and Temporal Transcriptomic Atlas of Mouse Intestinal Regeneration in the Jejunum.* (GitHub, https://github.com/tht38/intestinalspatialregen., 2025).

6 Ofer, R., Tran, T. & Verzi, M. P. *A Spatial and Temporal Transcriptomic Atlas of Mouse Intestinal Regeneration in the Jejunum* (https://doi.org/10.5281/zenodo.21348331, Zenodo, 2025).

7 Moor, A. E. *et al.* Spatial Reconstruction of Single Enterocytes Uncovers Broad Zonation along the Intestinal Villus Axis. *Cell* **175**, 1156–1167 e1115 (2018). https://doi.org/10.1016/j.cell.2018.08.063

8 Mootha, V. K. *et al.* PGC-1alpha-responsive genes involved in oxidative phosphorylation are coordinately downregulated in human diabetes. *Nat Genet* **34**, 267–273 (2003). https://doi.org/10.1038/ng1180

9 Subramanian, A. *et al.* Gene set enrichment analysis: a knowledge-based approach for interpreting genome-wide expression profiles. *Proc Natl Acad Sci U S A* **102**, 15545–15550 (2005). https://doi.org/10.1073/pnas.0506580102

10 Constant, D. A. *et al.* Homeostatic pockets of interferon lambda-stimulated gene production in the intestine are associated with localized exposure to bacterial microbiota. *Gut Microbes* **17**, 2447830 (2025). https://doi.org/10.1080/19490976.2024.2447830

11 Huang da, W., Sherman, B. T. & Lempicki, R. A. Systematic and integrative analysis of large gene lists using DAVID bioinformatics resources. *Nat Protoc* **4**, 44–57 (2009). https://doi.org/10.1038/nprot.2008.211

12 Sherman, B. T. *et al.* DAVID: a web server for functional enrichment analysis and functional annotation of gene lists (2021 update). *Nucleic Acids Res* **50**, W216–W221 (2022). https://doi.org/10.1093/nar/gkac194

13 Nusse, Y. M. *et al.* Parasitic helminths induce fetal-like reversion in the intestinal stem cell niche. *Nature* **559**, 109–113 (2018). https://doi.org/10.1038/s41586-018-0257-1

14 Li, J. *et al.* Identification and multimodal characterization of a specialized epithelial cell type associated with Crohn's disease. *Nat Commun* **15**, 7204 (2024). https://doi.org/10.1038/s41467-024-51580-7

15 Chen, L. *et al.* TGFB1 induces fetal reprogramming and enhances intestinal regeneration. *Cell Stem Cell* **30**, 1520–1537.e1528 (2023). https://doi.org/10.1016/j.stem.2023.09.015

16 Jin, S., Plikus, M. V. & Nie, Q. CellChat for systematic analysis of cell-cell communication from single-cell transcriptomics. *Nat Protoc* **20**, 180–219 (2025). https://doi.org/10.1038/s41596-024-01045-4

Methods related:

- Visium HD FFPE Tissue Preparation Handbook, Document Number CG000684 Rev D, 10x Genomics.
- Visium CytAssist Spatial Gene Expression for FFPE – Tissue Preparation Guide, Document Number CG000518 Rev C, 10x Genomics.
- Visium HD Spatial Applications Imaging Guidelines, Document Number CG000688 Rev C, 10x Genomics.
- Visium HD Spatial Gene Expression Reagent Kits User Guide, Document Number CG000685 Rev D, 10x Genomics.
