# Supplementary material for: A spatial and temporal transcriptomic atlas of mouse intestinal regeneration in the jejunum
Source: Nat Commun. 2026 Sep 3;17:10484. doi: 10.1038/s41467-026-77292-8 (PMC13635233; doi:10.1038/s41467-026-77292-8)
Supplement: Supplementary file 4 [file 41467_2026_77292_MOESM2_ESM.pdf]

Description of Supplementary Data

Supplementary Data 1 (Fig. 1e, f, and g):
Gene lists for each of the five epithelial zones (villus tip, upper villus, bottom villus, crypt-villus junction, and crypt) at Day 0, identified via differential gene expression (DEG) analysis between the epithelial zones. Includes gene ontology (GO) terms for each zone. Data shown from replicate 2, cell segmentation.

Supplementary Data 2 (Fig. 1j):
GO term enrichment analysis comparing *Isg15*$^{+}$ versus *Isg15*$^{-}$ cells on day 0 (Rep2).

Supplementary Data 3 (Supplementary Fig. 2a, e, f and g):
DEG analysis comparing *Ifit1*$^{+}$ versus *Ifit1*$^{-}$ bins in the junction to tip epithelial bins of day 0 (Supplementary Fig. 2a), and DEG analysis comparing *Ifit2*$^{+}$ versus *Ifit2*$^{-}$ bins in the junction epithelial bins of day 3 post-IR, highlighting genes with potential role in interferon signaling propagation (Supplementary Fig. 2e, f and g).

Supplementary Data 4: Gene sets used throughout the paper
Gene sets used in the paper, including epithelial enriched gene sets (Ayyaz et al., 2019; Yui et al., 2018; Chen et al., 2023; Mustata et al., 2013; Munoz et al., 2012; Wang et al., 2019; whole epithelium bulk-RNAseq (methods)) and GSEA analysis (Moor et al., 2018, Constant et al., 2025, Munoz et al., 2012, Mustata et al., 2013).

Supplementary Data 5 (Fig. 2h):
DEG and GO term analysis comparing bottom villus at Day 0 versus Day 3 (Rep2).

Supplementary Data 6 (Supplementary Fig. 3c):
DEG and GO term analysis comparing bottom villus at Day 0 versus bottom villus Day 3 (Rep3).

Supplementary Data 7 (Fig. 3i, j and k):
DEG and GO term analysis comparing the early regenerative population to the late regenerative population (Fig. 3i and j) and gene set used for GSEA analysis (Fig. 3k)

Supplementary Data 8 (Fig. 4i):
DEG analysis comparing cells with high- versus low-UMI (Unique Molecular Identifier) counts (Rep2).

Supplementary Data 9 (Fig. 5b):
DEG and GO term enrichment for stromal subpopulations (villus, inter-villus and sub-crypt) at Day 0 (Rep2).

Supplementary Data 10 (Fig. 5e):
DEG and GO term analysis comparing villus stromal populations across Day 0, Day 3 and Day 5 (Rep2).

Supplementary Data 11 (Fig. 5f):
DEG and GO term analysis comparing stromal populations (inter-crypt) across Day 0, Day 3 and Day 5 (Rep2).

Supplementary Data 12 (extension to Fig. 5f):
DEG and GO term analysis comparing stromal populations (sub-crypt) across Day 0, Day 3 and Day 5 (Rep2).

Supplementary Data 13 (Fig. 5g):
Predicted cell–cell interaction data.
